# Complete organelle genomes of Korean fir, *Abies koreana* and phylogenomics of the gymnosperm genus *Abies* using nuclear and cytoplasmic DNA sequence data

**DOI:** 10.1038/s41598-024-58253-x

**Published:** 2024-04-01

**Authors:** Seongjun Park, Myounghai Kwak, SeonJoo Park

**Affiliations:** 1 Institute of Natural Science, Yeungnam University, Gyeongsan, Gyeongbuk 38541 South Korea; 2 National Institute of Biological Resources, Incheon, 22689 South Korea; 3 Department of Life Sciences, Yeungnam University, Gyeongsan, Gyeongbuk 38541 South Korea

**Keywords:** Plant sciences, Plant evolution, Mitochondrial genome, Phylogenetics

## Abstract

*Abies koreana* E.H.Wilson is an endangered evergreen coniferous tree that is native to high altitudes in South Korea and susceptible to the effects of climate change. Hybridization and reticulate evolution have been reported in the genus; therefore, multigene datasets from nuclear and cytoplasmic genomes are needed to better understand its evolutionary history. Using the Illumina NovaSeq 6000 and Oxford Nanopore Technologies (ONT) PromethION platforms, we generated complete mitochondrial (1,174,803 bp) and plastid (121,341 bp) genomes from *A. koreana*. The mitochondrial genome is highly dynamic, transitioning from *cis*- to *trans*-splicing and breaking conserved gene clusters. In the plastome, the ONT reads revealed two structural conformations of *A. koreana*. The short inverted repeats (1186 bp) of the *A. koreana* plastome are associated with different structural types. Transcriptomic sequencing revealed 1356 sites of C-to-U RNA editing in the 41 mitochondrial genes. Using *A. koreana* as a reference, we additionally produced nuclear and organelle genomic sequences from eight *Abies* species and generated multiple datasets for maximum likelihood and network analyses. Three sections (*Balsamea*, *Momi*, and *Pseudopicea*) were well grouped in the nuclear phylogeny, but the phylogenomic relationships showed conflicting signals in the mitochondrial and plastid genomes, indicating a complicated evolutionary history that may have included introgressive hybridization. The obtained data illustrate that phylogenomic analyses based on sequences from differently inherited organelle genomes have resulted in conflicting trees. Organelle capture, organelle genome recombination, and incomplete lineage sorting in an ancestral heteroplasmic individual can contribute to phylogenomic discordance. We provide strong support for the relationships within *Abies* and new insights into the phylogenomic complexity of this genus.

## Introduction

*Abies* Mill. (Fir) is a genus of 48 evergreen conifers in the family Pinaceae that is native to North and Central America, Europe, Asia, and North Africa^1^. The genus *Abies* is distinguished from the other ten Pinaceae genera by morphological traits, including erect seed cones and deciduous seed scales at maturity with a persistent cone rachis^1^. Based on analyses of the internal transcribed spacer (ITS) region of nuclear ribosomal DNA (nrDNA), single-copy nuclear genes, plastid DNA, and mitochondrial DNA sequences^2–6^, *Abies* is a monophyletic group sister to the genus *Keteleeria*. The monophyly of sections is well supported, but the evolutionary relationships within the sections are not fully resolved due to low bootstrap values or polytomies. Recurrent hybridization influences the critical evolutionary processes within the genus *Abies*^2,7,8^. Genomic resources can provide the opportunity for a comprehensive understanding of the origin and evolution of this hierarchical complexity. Recent research has examined the genome-scale biogeography of the genus *Abies*; however, only the mitogenome has been used^9^.

Gymnosperms comprise 12 families and 83 genera containing approximately 1079 species of Cycadophyta (cycads), Ginkgophyta, Pinophyta, and Gnetophyta^10^. These groups show dynamic modes of inheritance in organelle genomes. For example, both organelle genomes of cycads, *Ginkgo*, and gnetophytes, are maternally inherited^11,12^. However, most species of Pinaceae and Taxaceae (Pinophyta) show maternal inheritance of the mitogenome but paternal inheritance of the plastome^11^. Because of this, it is challenging to infer the evolutionary history of Pinaceae and Taxaceae using only one genome. Therefore, three genomic sequences are required to pinpoint maternal, paternal, and biparental inheritance patterns to fully understand their evolutionary histories.

Next-generation sequencing (NGS) techniques and bioinformatic tools provide genomic-scale data for genomic and phylogenomic studies at a wide range of taxonomic levels. At least 200 gymnosperm plastomes have been sequenced, but only a few mitogenomes are available yet according to the NCBI Genome database, which was accessed on December 11, 2023. These genomic data show that gymnosperm plastomes and mitogenomes are diverse in their structural evolution. The plastomes of cycads, *Ginkgo*, and gnetophytes contain a typical inverted region (IR), whereas those of Pinophyta lacks a large IR^13–15^. However, the plastomes of Pinophyta have highly reduced repeats that generate substoichiometric isomers by homologous recombination^13^. Gymnosperm plastomes contain 66–87 protein-coding genes, 28–35 tRNA genes, and four rRNA genes, ranging from 85.3 kb (*Parasitaxu*s in Pinophyta) to 166.3 kb (*Macrozamia* in cycads) in size, and exhibit inversion, relocation, and losses of genes^16–18^. In contrast, only five gymnosperm mitogenomes have been assembled into circular structures (*Cycas taitungensis*^19^, *Cycas debaoensis*^20^, *Ginkgo biloba*^21^,* Welwitschia mirabilis*^21^, and *Taxus cuspidate*^22^), and eight have been assembled into linear scaffolds or both linear and circular structures (three *Abies* [see below], *Larix sibirica*^23^, *Picea abies*^24^, *Picea sitchensis*^25^, *Picea glauca*^26^, and *Pinus taeda*^27^). The sizes of the mitogenomes are also variable, ranging from 346.5 kb in *G. biloba*^21^ to 11.69 Mb in *L. sibirica*^23^ from Pinophyta and contain 29–41 protein-coding genes, 28–35 tRNA genes, and three rRNA genes. They show various features, including mitochondrial DNA of plastid (MIPTs) and nuclear (MINCs) origin, repeats, and RNA editing^19,22,28,29^.

To date, 43 plastomes from 25 *Abies* taxa have been sequenced based on the NCBI Genome database, which was accessed on December 11, 2023, ranging from 119.4 kb (*A. religiosa*) to 121.8 kb (*A. fargessii*) and containing 72 protein-coding genes with 16 introns. All 11 *ndh* genes have been lost in these plastomes. However, draft mitogenome sequences of only three species of *Abies* have been reported: *A. alba* assembled into 11 scaffolds (1.43 Mb)^30^, *A. firma* assembled into 172 scaffolds (1.33 Mb)^28^, and *A. sibirica* assembled into 237 scaffolds (1.49 Mb)^31^. These genomic data suggest that the ancestral *Abies* mitogenome contains 41 protein genes with 26 introns.

PacBio SMRT or Oxford Nanopore Technologies (ONT) sequencing provides opportunities to assemble large and complex genomes with long reads^25^, but it is still challenging to complete. To improve our understanding of the evolution of organelle genomes in the genus *Abies*, we sequenced the complete mitochondrial and plastid genomes from Korean fir, *Abies koreana* and characterized MIPTs and repeats in the complete mitogenome and RNA editing in 41 mitochondrial genes*.* To reconstruct a genome-scale phylogeny for the *Abies*, we sequenced complete plastomes and extracted these mitochondrial genes from Illumina assemblies of eight additional species. In addition, we analyzed the nuclear single-copy genes as well as ribosomal DNA (rDNA) clusters, which include loci for the 5' external transcribed spacer (5'-ETS), 18S, ITS1, 5.8S, ITS2, and 26S rDNA regions. Finally, we used these data to explore the phylogenomic evidence for the selected *Abies* relationships.

## Results

### Mitochondrial and plastid genome organization of *A. koreana*

Using Illumina NovaSeq 6000 and ONT PromethION sequencing, we obtained a total of 551,969,293 paired-end reads (100 bp × 2) and 1,865,257 long reads (average length 14,207 bp, range 1–3,387,768 bp) for *A. koreana*. The mitochondrial and plastid genomes of *A. koreana* were assembled by Velvet, Spades, and MaSuRCA using Illumina and Oxford reads with deep coverage (PE/ONT; 418/224-fold for mitogenome, PE/ONT; 1950/543-fold for plastome).

**Figure 1:**
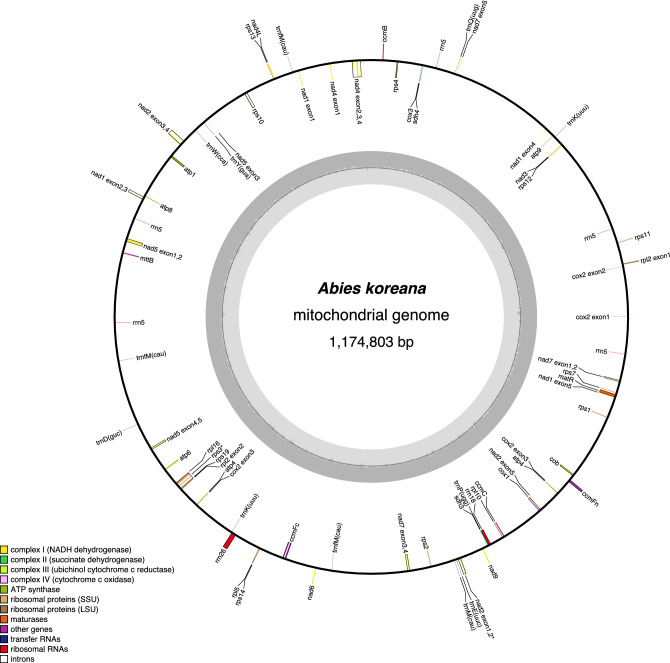
Circle map of the mitochondrial genome of *Abies koreana*. Genes on the inside and outside the map are transcribed in the clockwise and counterclockwise directions, respectively.

**Figure 2:**
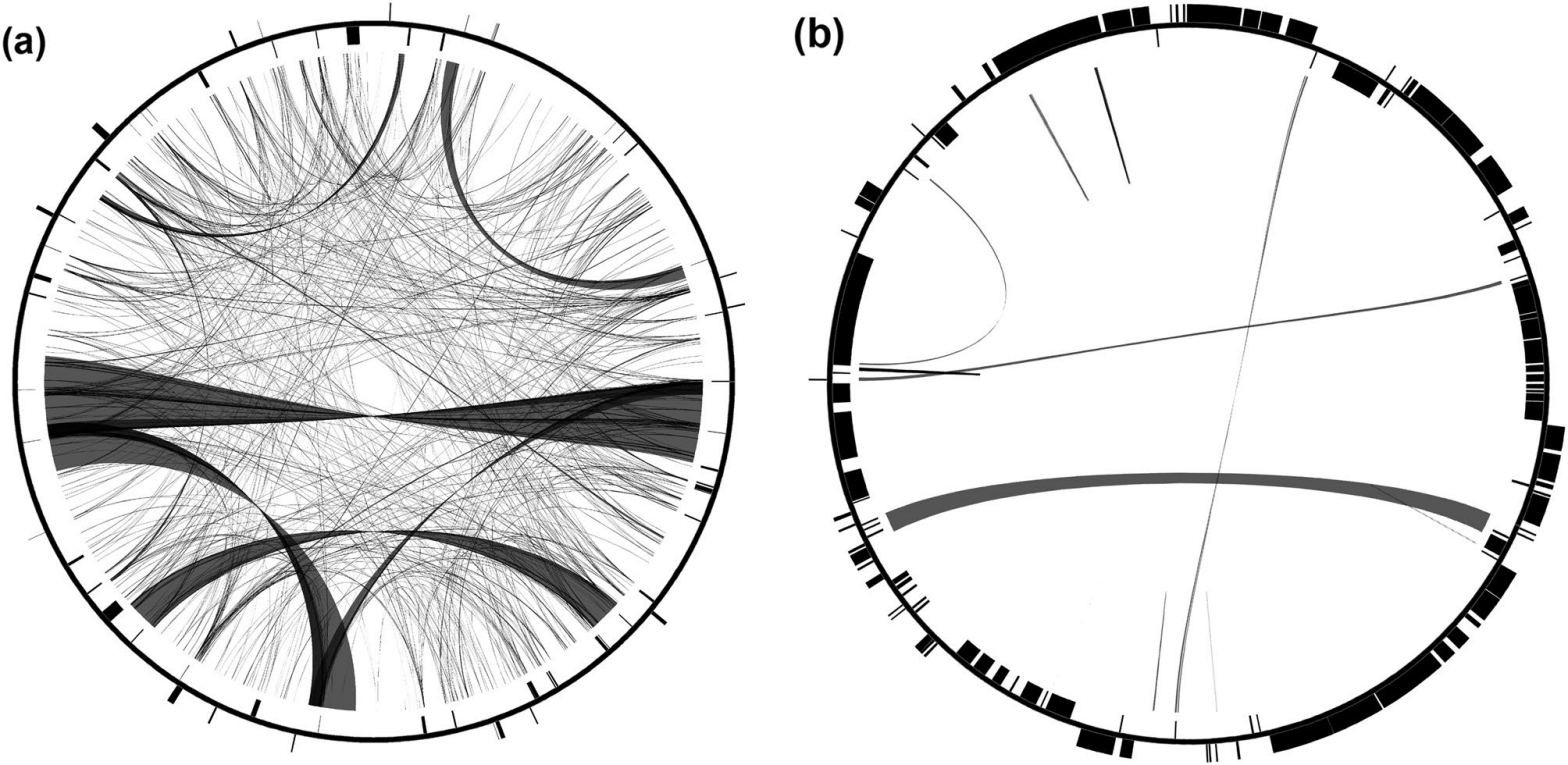
Distribution of repetitive DNA in *Abies koreana* organelle genomes. Within the circular maps, black lines represent the locations of pairs of repeats, with crossing lines denoting reverse repeats. In the inner and outer circles, the black boxes denote the locations of the mitochondrial genes. (**a**). mitochondrial genome. (**b**) plastid genome.

The *A. koreana* mitogenome was assembled into a circular molecule with a genome size of 1,174,803 bp (Table 1 and Fig. 1). The GC content was 45.9%, and the genome consisted of 3.08% protein-coding genes, 96.39% noncoding regions, 0.06% tRNA genes, and 0.47% rRNA genes (Table 1). The *A. koreana* mitogenome contained 153,910 bp of repetitive DNA (13.1%) (Table and Fig. 2a), ranging from 30 to 27,029 bp in length, including nine large (> 1000 bp), 36 intermediate (100–1000 bp), and 525 small (< 100 bp) repeats (Table S1). MIPT sequences were found in six fragments (504 bp) throughout the *A. koreana* mitogenome ranging from 68 to 106 bp in length (Table S2). Four partial genes (*petA*, *psaA*, *psbK*, and *ycf1*) and one tRNA gene (*trnW-CCA*) were identified. No *Bpu*-like elements were detected in the *A. koreana* mitogenome. The *A. koreana* mitogenome also contained 48,022 bp of diverse transposable elements (TEs) (Table S3). The majority of these TEs were long terminal repeat (LTR) retrotransposons (*copia*- and *gypsy*-like) (29,809 bp; 62.1%). The mitogenome of *A. koreana* contained 41 protein-coding genes, nine tRNAs, and three rRNAs (Table 1). In *Abies koreana*, 11 of the 14 clusters inferred from the ancestral angiosperm mitochondrial genome were missing: *nad3-rps12*, *rpl16-rps3-rps19-rpl2*, and *trnP-sdh3* (Table S4). Twenty-six group II introns were found in the ten genes, 13 and 13 of which were *cis*- and *trans*-spliced, respectively (Fig. 1). Six ORFs of at least 150 bp in length, two (*orf97* and *orf70*) of which were present as two copies, appeared to be chimeric ORFs containing small fragments (> 30 bp) of mitochondrial genes (Table S5). One (*orf69*) of these ORFs was predicted to encode one transmembrane helix containing small fragments of the *ccmC* gene (Fig. S1).

**Table 1:** Characteristics of *Abies koreana* organelle genomes.

	Mitochondrial genome	Plastid genome
Genome size (bp)	1,174,803	121,341
GC content (%)	45.9	38.3
Genes		
Protein-coding genes	41	72 (2)
(%)	3.08	50.29
tRNA genes	9	34 (1)
(%)	0.06	2.18
rRNA genes	3 (1)	4
(%)	0.47	3.73
Introns		
* cis*-spliced group	13	15
* trans*-spliced group	13	1
Plastid-derived		
Protein-coding genes	4	–
tRNA genes	1	–
Repeats (bp)	153,910	2357

PREP-Mt with a cutoff of 0.2 predicted 1268 putative C-to-U RNA editing sites in the 41 *A. koreana* mitochondrial protein-coding genes (Tables S6 and S7). Mapping of the RNA-seq reads and alignment of transcripts revealed 1356 C-to-U RNA editing sites and confirmed the 1122 sites predicted by PREP-Mt for the 41 mitochondrial genes. Moreover, 116 silent RNA editing sites were detected (Table S7). The ten *A. koreana* mitochondrial genes (*atp6*, *cox1, mttB*, *nad1, nad6*, *nad9*, *rpl2*, *rps3*, *rps4*, and *rps10*) had an ACG start codon that was RNA edited to AUG in their transcript. In the *atp4*, *atp6*, *atp9*, *ccmFc*, *cox1*, *nad4L*, *nad4*, *rpl2*, *rpl16*, and *sdh3* genes, premature stop codons were created by C-to-U RNA editing. Among them, the *A. koreana cox1* gene showed 16 sites of C-to-U editing, creating a stop codon, and then 23 sites of C-to-U editing generated a new AUG start codon (Tables S6 and S7, and Fig. S2). The *A. koreana rpl16* gene had a GTG codon, and *rps19* had a GCG codon (RNA edited to GUG in its transcript) as an alternative start codon (Fig. S3). The gene most affected by RNA editing was *nad5*, for which 108 changes caused 98 codon changes (Table S6). The highest density of RNA editing sites was in *atp6*, in which 61 out of 777 nucleotides were edited (Table S6). Leucine was mostly affected by C-to-U editing, with 41.85% (519 sites) codon changes, and 23.95% (297 sites) of its amino acids were converted to phenylalanine (Table S6).

**Figure 3:**
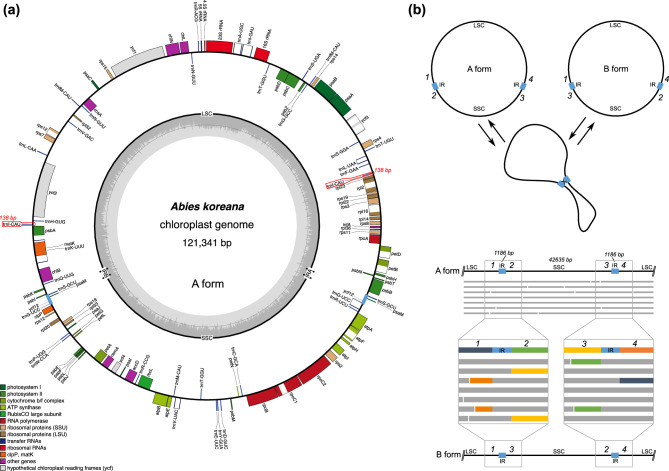
Plastid genome of *Abies koreana*. (**a**) Circle map of the A form. Genes on the inside and outside the map are transcribed in the clockwise and counterclockwise directions, respectively. (**b**) Formation of two isoforms. Intermolecular recombination across inverted repeats (blue). ONT reads were aligned to the plastid genome of *A. koreana*. The gray parts of the ONT reads indicate that those regions are identical to the parts of the reference genome. The dark blue, green, orange, and yellow parts of the ONT reads could not be aligned with the reference regions, which are corresponding regions. The four single-copy genomic regions surrounding the IR are shown with numbers and colors.

The plastome of *A. koreana* was 121,341 bp with a pair of inverted repeats of 1186 bp separated by a small single-copy (SSC) region (42,635 bp) and a large single-copy (LSC) region (76,334 bp) (Table 1 and Fig. 3a). Long reads provided strong evidence for the repeat-mediated rearrangement activity of the *A. koreana* plastome (Fig. 2b). Mapping of long reads to the plastome “form A” showed that many of the reads were consistent with the reference form except for the conflicting reads (Fig. 3b). The inconsistent areas were indicated with the corresponding regions of plastome “form B”, which identified the boundaries of the repeats (Fig. 3b). The *A. koreana* plastome contained 2357 bp of repetitive DNA (Table 1 and Fig. 3b), ranging from 32 to 1186 bp in length (Table S8). The GC content was 38.3%, and the genome consisted of 50.29% protein-coding genes, 43.80% noncoding regions, 2.18% tRNA genes, and 3.73% rRNA genes. The plastome contained 72 protein-coding genes, 34 tRNA genes, and four rRNA genes (Table 1). The short IR region contained the *trnS*-GCU, *psaM*, *ycf12*, and partial *trnG*-UCC genes (Fig. 3).

### *Abies* plastid genomes, mitochondrial genes, and nuclear DNAs

We determined complete plastomes from eight additional species spanning three sections of *Abies*, including *Balsamea* (*A. sibirica*, *A. nephrolepis*, *A. sachalinensis*, and *A. veitchii*), *Momi* (*A. pindrow, A. firma*, and *A. kawakamii*), and *Pseudopicea* (*A. spectabilis*). The assembled *Abies* plastomes were similar in terms of their organization, size and gene/intron content to those of the *A. koreana* plastome (Table S9). In thems of gene content, the *A*. *kawakamii* plastome contained two copies of the *trnH-GUG* gene with 97.3% nucleotide sequence identity. The duplicated copy was located downstream from the *trnT-GGU* gene (approximately 31 kb from the original copy). Among the eight species of *Abies*, *A*. *kawakamii* had the largest (121,803 bp) plastome, and *A. spectabilis* had the smallest (119,914 bp) plastome. The short IR size ranged from 1172 (*A. spectabilis*) to 1186 bp (*A. firma*, *A. nephrolepis*, *A. sachalinensis*, and *A. veitchii*). Using the mitochondrial gene sequences from *A. koreana*, we obtained 41 mitochondrial-encoded genes and 13 *cis*-spliced introns (*ccmFci829*, *nad1i477*, *nad2i156*, *nad2i709*, *nad4i1399*, *nad4i976*, *nad5i230*, *nad5i1872*, *nad7i140*, *nad7i676*, *rps3i74*, *rps3i257*, and *rps10i235*) for an additional eight *Abies* species.

We obtained nuclear single-copy gene sequences from the nine *Abies* PE reads using Read2Tree with a total of 1000 orthologs from *Picea glauca* and *Pinus taeda* and used only 452 single-copy orthologs that were present in the 11 analyzed species. In addition, we recovered nine nuclear rDNA sequences, including 5ʹ-ETS, 18S, ITS1, 5.8S, ITS2, and 26S, which ranged in size from 7877 bp (*A. sibirica*) to 7906 bp (*A*. *kawakamii*) (Fig. S4). The length of ITS1 ranged from 1228 to 1257 bp, and that of ITS2 was 244 bp. The length of the partial 5ʹ-ETS region ranged from 1043 to 1044 bp in length.

### *Abies* phylogeny based on plastid, mitochondrial, and nuclear sequences

**Figure 4:**
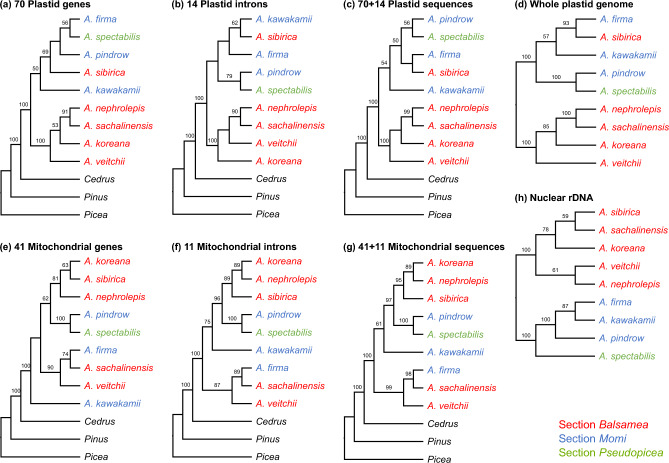
Phylogenetic relationships among the analyzed *Abies* species. Cladograms based on plastid (**a**–**d**), mitochondrial (**e**–**g**), and nuclear (**h**) sequences. Maximum likelihood bootstrap support values are shown above the branches on each cladogram.

To examine the phylogenetic relationships among the nine *Abies* species, we performed phylogenomic analyses using a concatenated alignment of plastid and mitochondrial protein-coding genes (plastid: 61,506 aligned nucleotide positions; mitochondrial: 34,501 aligned nucleotide positions), adding introns (plastid: 10,264 aligned nucleotide positions; mitochondrial: 23,302 aligned nucleotide positions), or a whole plastome alignment (122,389 aligned nucleotide positions) (Fig. 4a–g). The cladogram, based on plastid datasets, indicates that *Abies* is strongly supported as monophyletic according to the bootstrap (BS) values of 100% and is sister to *Cedrus* (BS = 100%) (Fig. 4a–d) and contains two major clades. One clade comprising four species of sect. *Balsamea* except *A. sibirica* is strongly supported (BS = 100%). *Abies sibirica* is consistently nested within the other clade with strong support (BS = 100%, whole plastid dataset). However, there were conflicts in the relationships among *A. firma*, *A. spectabilis*, *A. pindrow*, *A. sibirica*, and *A. kawakamii* according to the datasets (Fig. 4a–d). The concatenated alignment of all 41 mitochondrial protein-coding genes and 11 introns revealed a topology with relatively strong bootstrap support (Fig. 4e–g). We excluded two mitochondrial introns (*ccmFci829* and *nad1i477*) due to the extreme size variation in the outgroups. However, we additionally generated a 41-gene/13-intron data matrix with 56,378 aligned nucleotide positions for only *Abies* species, showing that the topology is consistent with the phylogenetic tree, including outgroups (Fig. S5). In contrast to the plastid results, *A. sachalinensis* and *A. veitchii* of sect. *Balsamea* were sisters to *A. firma* of sect. *Momi* (BS = 99%). *Abies pindrow*, *A. spectabilis*, and *A. kawakamii* were sisters to *A. koreana*, *A. nephrolepis*, and *A. sibirica* (BS = 100%; Fig. S5). Moreover, *A. sibirica* was sister to *A. koreana* and *A. nephrolepis* with strong support (BS = 95%).

**Figure 5:**
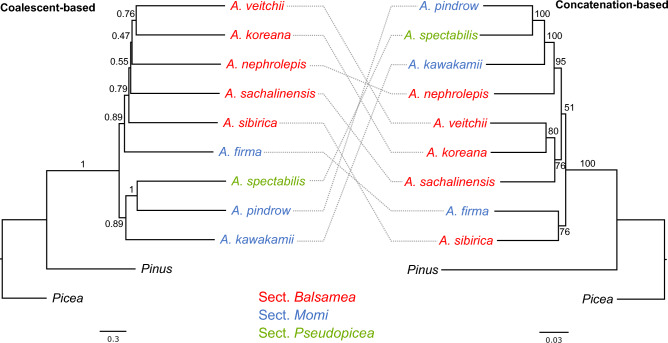
Phylogenomic trees of the nuclear single-copy genes inferred with ASTRAL-III and IQ-TREE2. Local posterior probability and maximum likelihood bootstrap support values are shown above the branches on each phylogram.

Phylogenomic analyses of the genus *Abies* based on nuclear rDNA sequences and single-copy gene sequences were also performed (Figs. 4h and 5). In particular, we conducted phylogenomic analyses of 452 nuclear single-copy genes employing coalescent- and concatenation-based methods. Our results revealed that the nuclear phylogenies were not congruent with those of the organellar topology. The cladogram, based on the nuclear rDNA datasets (7918 aligned nucleotide positions), provided strong support for the monophyly of *A. koreana*, *A. nephrolepis, A. sachalinensis, A. sibirica,* and *A. veitchii* from sect. *Balsamea* (BS = 100%) and for *A. firma*, *A. pindrow*, and *A. kawakamii* from sect. *Momi* (BS = 100%). A sister relationship between sect. *Momi* and *A. spectabilis* was strongly supported (BS = 100%). *Abies koreana* was sister to *A. sibirica* and *A. sachalinensis* (BS = 78%). The topologies of the Accurate Species TRee ALgorithm (ASTRAL) and the maximum likelihood (ML) supermatrix (441,999 aligned nucleotide positions) based on 452 genes were not identical (Fig. 5). The ASTRAL tree showed two major clades: one clade comprised five species of sect. *Balsamea* and *A. firma* from sect. *Momi* and the other was composed of *A. spectabilis*, *A. pindrow*, and *A. kawakamii*. In contrast to the ASTRAL tree, the concatenated ML tree showed that *A. spectabilis*, *A. pindrow*, and *A. kawakamii* were nested with the remaining *Abies* species.

To test phylogenetic incongruence, we compared four topologies (the whole plastome, 41 + 13 mitochondrial, nuclear rDNA and 452 nuclear gene sequences) by performing the approximately unbiased (AU), Shimodaira-Hasegawa (SH), and Kishino-Hasegawa (KH) tests (Fig. S6). For each dataset, the alternative topologies were significantly rejected, with a *p* value less than 0.05 for all tests. The four spilt networks among the nine *Abies* species were consistent with the ML analyses based on the nuclear, plastid, and mitochondrial datasets (Fig. S7). In addition, the pairwise homoplasy index (PHI) test indicated that there were recombination signals in the plastid (*p* = 0.002131) and mitochondrial (*p* = 0.00006591) datasets.

**Figure 6:**
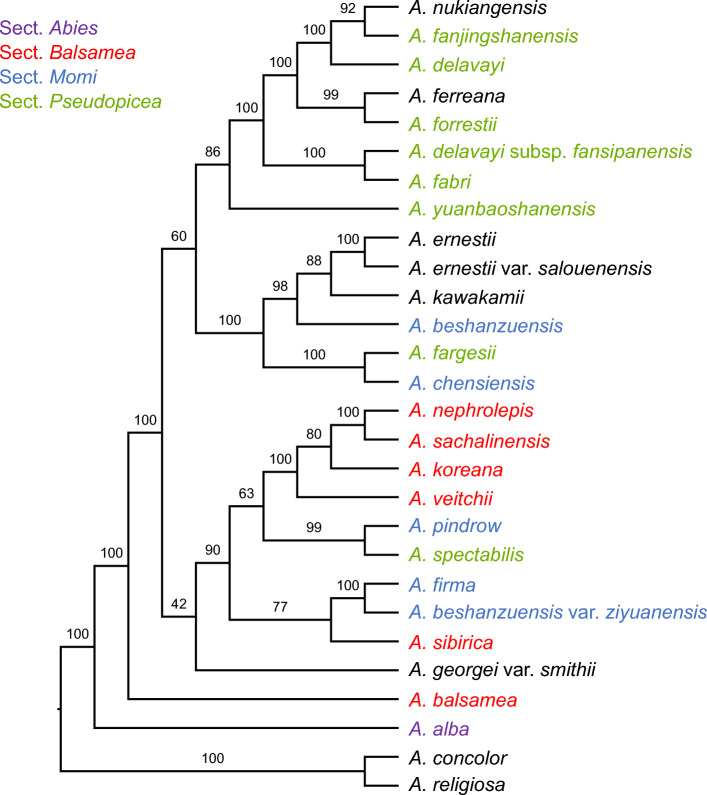
Phylogenetic relationships among the 25 *Abies* species based on whole plastid genomes. Maximum likelihood bootstrap support values > 50% are shown above the branches on each cladogram.

To further investigate the evolutionary relationships with the genus *Abies*, 19 additional *Abies* (*A. alba*, *A. balsamea*, *A. beshanzuensis*, *A. beshanzuensis* var. *ziyuanensis*, *A. chensiensis*, *A. concolor*, *A. delavayi*, *A. delavayi* subsp. *fansipanensis*, *A. ernestii*, *A. ernestii* var. *salouenensis*, *A. fabri*, *A. fanjingshanensis*, *A. fargesii*, *A. ferreana*, *A. forrestii*, *A. georgei* var. *smithii*, *A. nukiangensis*, *A. religiosa*, and *A. yuanbaoshanensis*) plastomes were selected (Table S9). ML analysis of the whole plastome alignment of 25 *Abies* (132,729 aligned nucleotide positions) yielded largely congruent topologies according to the ML tree based on the whole plastid dataset of nine *Abies* (Fig. 6). The topology also showed the monophyly of *A. koreana*, *A. nephrolepis, A. sachalinensis,* and *A. veitchii* with bootstrap values of 100%. The topology showed the monophyly of *A. pindrow and A. spectabilis* with bootstrap values of 99%. The topology showed the monophyly of *A. firma and A. sibirica* with *A. beshanzuensis* var. *ziyuanensis* with bootstrap values of 100%. However, *A. kawakamii* nested with additional taxa and was sister to the *A. ernestii* group.

## Discussion

Organelle genomes are important sources of phylogenetic information for inferring relationships within the Pinaceae family because of their different modes of inheritance, in which the mitochondrial and plastid genomes are maternally and paternally transferred, respectively. However, mitogenomes have a more complex structure (circular chromosome, linear, or complicated multibranched linear molecules) than plastomes^32^, making assembly difficult. As a result, 43 plastomes from 25 *Abies* taxa were sequenced. However, only one mitogenome sequence is available in GenBank (although three mitogenomes have been published). Here, we generated the complete mitogenome of *A. koreana* as a circular molecule supported by a high depth of coverage (ONT reads: mean coverage is 224×) (Fig. 1). The *A. koreana* had the smallest genome (1.17 Mb) compared to the three *Abies* mitogenomes available for *A. alba* (1.43 Mb^30^), *A. firma* (1.33 Mb^28^), and *A. sibirica* (1.49 Mb^31^), which were assembled into the several scaffolds (11 to 237) (Table S10). However, multiple scaffolds in the genome assembly (especially *A. firma*: 172 and *A. sibirica*: 237) can contain overlapping or redundant regions, leading to an overestimation of genome size. Incomplete or repetitive regions can also result in the creation of separate contigs or scaffolds that may actually represent the same genomic region. To obtain a more accurate estimate of *Abies* genome size, complete mitogenomes are needed. In gymnosperms, the mitogenome size varies greatly, from 346 kb in *G. biloba*^21^ to 11.69 Mb in *L. sibirica*^23^. The size of the *A. koreana* mitogenome is similar to that of the *Pinus taeda* mitogenome (1.19 Mb)^27^, which is the median among the sequenced gymnosperm mitogenomes. A comparison of the *A. koreana* mitogenome with the five representative gymnosperm mitogenomes (*Cycas*, *Ginkgo*, *Pinus*, *Taxus*, and *Welwitschia*)^22^ revealed that MIPTs, MINCs, or repeats often influence variations in mitogenome size. In the *A. koreana* mitogenome, 154 kb repeats (13.1%) and 48 kb TEs (4.15%) were identified, whereas only 0.5 kb MIPTs (0.04%) were found. The amount of unidentified DNA (77.3%) contributed to the *A. koreana* mitogenome size; a nuclear genome is needed to fully understand the reasons for the mitogenome expansion.

Recombination of large repeats promotes genomic rearrangements, leading to the inference of numerous isoform maps^33^. Nine large repeats (> 1 kb) may be associated with homologous recombination in the *A. koreana* mitogenome, generating multiple subgenomic circles. Recombination can disrupt conserved gene clusters in the mitogenome. We found only three gene clusters in the *A. koreana* mitogenome (Table S3). The frequency of *trans*-splicing also suggests that chromosomal rearrangements are the predominant source of transitions from *cis*- to *trans*-splicing in plant mitochondria^31^. In the *A. koreana* mitogenome, 13 of 26 introns were *trans*-splicing, which is a high number compared to that of other gymnosperm mitogenomes. This phenomenon is also observed in Pinaceae mitogenomes, suggesting that a high rate of genomic rearrangement occurred in the common ancestor of Pinaceae. Mitochondrial gene transfer to the nucleus is also an ongoing process in gymnosperms^28^, and most of the transferred genes are ribosomal protein genes (*rps1*, *rps2*, *rps7*, *rps10*, *rps11*, *rsp14*, and *rpl2*) and one succinate dehydrogenase protein gene (*sdh3*). However, our results confirmed that the *A. koreana* mitogenome also retained all 41 protein-encoding genes. Although *A. koreana* contains a full set of protein-coding genes, we performed a BLAST search between the protein and *A. koreana* transcriptomes for intracellular gene transfer (IGT) to the nucleus as an intermediate stage. Mitochondrial genes must be transferred to the nucleus before being lost from the mitogenome, but no evidence for IGT was detected in the *A. koreana* transcriptome. Taken together, the genomic rearrangement was not correlated with the gene content of mitochondrial genes in the *A. koreana* mitogenome.

Compared with RNA editing in the five mitogenomes^22^, *A. koreana* mitochondrial genes retained high levels of C-to-U RNA editing sites (1268 sites). We also identified mitochondrial RNA editing sites in *A. koreana* by comparing RNA-seq raw reads and the transcriptome data, identifying 1356 C-to-U RNA editing sites in 41 mitochondrial genes. This difference occurred because PREP-MT predicted only nonsilent RNA editing sites in protein-encoding genes. In addition, these empirical data also detected start codons (*cox1*, *nad6*, *rpl2*, and *rps4*) and stop codons (*atp4*, *cox1*, *rpl2*, and *sdh3*), which were not predicted by PREP-Mt (Table S7). Cytidine-to-uridine editing can generate start or stop codons^34^. In the *A. koreana* mitogenome, 13 of 41 genes were affected by C-to-U editing of start or stop codons. RNA-editing sites were associated with all stop codons: CAA (Q) to TAA (X) in the *atp4*, *atp6*, *ccmFc*, and *rpl16* genes; CAG (Q) to TAG (X) in the *cox1* and *rpl2* genes; and CGA (R) to TGA (X) in the *atp9*, *nad4*, *nad4L*, and *sdh3* genes. The *A. koreana* mitochondrial *rpl16* and *rps19* genes showed the GUG in the transcripts. Translation initiation at GTG and ATA codons has been proposed for a number of plant mitochondrial genes, including *apt8*, *cob*, *mttB*, *rpl16*, and *rps12*^35–38^. These results suggest that the mitochondrial *rps19* gene of *A. koreana* can also be initiated at alternative start codons. In particular, a premature stop codon by RNA-editing in the *cox1* gene occurred at the N-terminus, followed by a new start codon. Potential functional replacement of the mitochondrial *cox1* gene by transfer to the nucleus in *A. koreana* was not detected, indicating that this gene probably encodes a functional protein in mitochondria.

The sequenced *A. koreana* plastome exhibited moderate variation in genome size and gene/intron content among the published Pinaceae plastomes. High reduction of the typical inverted repeats (IRs), which contain only *trnI-CAU* and a portion of 3' *psbA* (236–496 bp), is a well-known phenomenon in Pinaceae plastomes^39^. The plastomes of nine *Abies* species have an extremely reduced pair of typical IRs, including only *trnI-CAU* (139 bp). In Pinaceae plastomes, multiple isoforms have been identified due to recombination at small repeats that cause genomic rearrangements^13,40^. Within the genus *Abies*, two distinct forms (A and B) were observed depending on the species, and only the A form of *A. koreana* was detected by PCR^13^. However, for the *A. koreana* plastome reported here, four independent assemblies using short and long reads verified that there were alternative isoforms. Mapping of the long reads to the A form confirmed that *A. koreana* has two isoforms, which are achieved by homologous recombination with a pair of short repeats (1186 bp) (Fig. 3b).

The nuclear, plastid and mitochondrial datasets analyzed in this study (Figs. 4 and 5) represent the most extensive sets of genes from the three plant genomes for *Abies* phylogenetics. Our analyses were largely consistent with previous studies based on the nuclear ITS region^3^, five plastid genes, and a single nuclear locus^4^; and a combined analysis of six mitochondrial, plastid, and nuclear ITS regions^2^; and large portion of the mitochondrial genes^9^. Collectively, these studies demonstrate that *Abies* is monophyletic and sister to the genus *Keteleeria*. The extended phylogeny presented here (Fig. 6) is strongly inconsistent with the traditional classification^41^. Our analyses also suggested high levels of genome-tree conflict between nuclear and cytoplasmic genomes or between organellar genomes, although not all sections of the genus *Abies* were sampled in this study. The conflict between plastid and mitochondrial genomes is possibly due to the different inheritance of organelles in the genus *Abies*. The main causes of cytonuclear discordance are hybridization or introgression and incomplete lineage sorting. Previous studies and the finding of the present study suggest that recurrent hybridization and introgression events may have contributed to the evolutionary history of eastern Asian *Abies*^8,42^. Thus, these phenomena in the genus *Abies* occur via organellar genome capture^43^.

Combining mitochondrial protein-coding genes with introns, rather than only using mitochondrial protein-coding genes alone, is more advantageous for generating an abundance of variable characters (Fig. 4). Additionally, the use of nuclear 5ʹ-ETS region sequences for phylogenetic analysis has improved resolution. However, the presence of ITS polymorphisms has been observed in Pinaceae, which may be a result of a slow rate of concerted evolution^44^.

Given the incongruence among genomes for *Abies* relationships, we urge caution in combining datasets derived from different genomes to study the phylogenetics of the genus *Abies*. A full understanding of the complicated evolutionary history of the genus *Abies* requires extensive sampling of all three plant genomes (especially nuclear transcriptomes) from all sections.

## Conclusions

The complete mitogenome of *A. koreana* provides important additional information for improving the understanding of genome evolution among gymnosperms in terms of plastid-derived DNA sequences, repeats, and RNA editing sites. Our results also provide new insight into the structural variation in the *A. koreana* plastome. Based on the organelle genome-scale data with nrDNA, phylogenomic relationships among the nine analyzed *Abies* species were strongly supported, showing conflicting topologies. Overall, our research has greatly enriched the genome resources of *Abies*, which will help to further analyze the phylogeny and suggest new approaches for elucidating the evolutionary relationships within *Abies* in the future.

## Methods

### DNA isolation and sequencing

Fresh leaves of *A. koreana* were collected from a single tree in the garden of the National Institute of Biological Resources (NIBR, Incheon, South Korea) and a voucher specimen was deposited in the NIBR Herbarium (NIBRVP0000729725 identified by Myounghai Kwak). The experimental study of the plant, including collection of the material, complied with institutional, national, and international guidelines. Total genomic DNA was extracted from the dried bulbus by grinding them with liquid nitrogen using the cetyltrimethylammonium bromide (CTAB) method^45^ and then sequenced to generate 100-bp × 2 paired-end (PE) reads from a 550-bp library using the Illumina NovaSeq 6000 platform (Illumina, San Diego, CA). A single flow cell of long reads was sequenced using the Oxford Nanopore Technologies PromethION platform (ONT, Oxford, United Kingdom). Additional gDNA from seven *Abies* species (*A. kawakamii*, *A. nephrolepis*, *A. pindrow*, *A. sachalinensis*, *A. sibirica*, *A. spectabilis*, and *A. veitchii*) was extracted and sequenced to generate 150-bp × 2 PE reads using the Illumina NovaSeq 6000 platform.

### Genome assembly, annotations, and analyses

The *A. koreana* plastid and mitochondrial genomes were generated from contigs produced by Velvet v1.2.10^46^, SPAdes v3.13.1^47^, and MaSuRCA v3.2.6^48^. For Velvet assemblies, each run was performed with only PE reads using multiple *k*-mers (69 to 93) and expected coverage values (50, 100, 200, 500, and 1000). For SPAdes assemblies, the coverage cutoff was set to 20, 50, and 100 with mismatch correction mode (-careful) using both PE and ONT reads. For MaSuRCA assembly, a configuration file containing default assembly parameters was created with both PE and ONT reads. Illumina data for the seven *Abies* species were assembled with Velvet as described above for *A. koreana*. For each species, nuclear ribosomal DNA (nrDNA), plastid, and mitochondrial contigs were identified using a BLAST-like algorithm in Geneious Prime 2022.0.1 (http://www.geneious.com) with the organelle genes of *Pinus taeda* plastid (NC_021440) and mitochondrial (NC_039746) genomes as queries. The consensus nrDNA, plastid, and mitochondrial genome sequences were completed by manually aligning the overlapping contigs. Genome sequencing data were obtained from the NCBI Sequence Read Archive (SRA) for one additional *Abies* species (*A. firma*; SRR12710828) and three additional Pinaceae species (*Cedrus deodara*; SRR12710827, *Picea smithiana*; SRR12710829, and *Pinus armandii*; SRR12710830), and the nrDNA and plastid genome sequences were recovered as described above.

Circular plastid and mitochondrial genome maps were drawn using OrganellarGenomeDRAW (OGDRAW) v1.3.1 (https://chlorobox.mpimp-golm.mpg.de/OGDraw.html)^49^. The newly generated plastid, mitochondrial, and nrDNA sequences were deposited in GenBank (Table S8) and the Dryad Digital Repository.

Using ROUSFinder.py^50^, repetitive sequences in the *A. koreana* mitochondrial and plastid genomes were discovered. MIPT sequences were identified by performing BLASTN searches of the *A. koreana* plastid genome against the mitochondrial genome in Geneious Prime with an e-value cutoff of 1e-10, at least 80% sequence identity and a minimum length of 50 bp. The "Find ORFs" with an ATG start codon in Geneious Prime were used to find open reading frames (ORFs) in the mitochondrial genome of *A. koreana* that were longer than 150 bp. To search for chimeric ORFs, all ORFs were compared with annotated *A. koreana* mitochondrial genes using BLASTN with an e-value cutoff of 1e−3, minimum length of 30 bp (as described in Mower et al.^51^) and at least 90% sequence identity. Using the TMHMM server v.2.0^52^, transmembrane helices in the selected ORFs were predicted.

RNA editing sites for mitochondrial genes were predicted using PREP-Mt^53^ with a cutoff value of 0.2. To check the empirical RNA editing sites on the protein-coding genes, paired-end Illumina sequence reads were also downloaded from the NCBI SRA repository (SRR5747840). We performed error correction for the raw reads using Rcorrector v1.0.4^54^ and then mapped the corrected reads to the genomic gene sequences using Bowtie v2.2.9^55^. In addition, the transcriptome was assembled by Trinity^56^ with the “trimmomatic” option using the corrected reads. The available mitochondrial transcripts in the transcriptome were identified using BLAST + v2.12.0^57^ and then aligned with the genomic gene sequences.

### Phylogenetic analyses

Each organelle gene was aligned based on the back-translation approach with MAFFT v7.450^58^ in Geneious Prime 2022.0.1. Nuclear rDNA sequences (5ʹ-ETS, 18S, ITS1, 5.8S, ITS2, and 26S) were aligned using MUSCLE v3.8.425^59^ in Geneious Prime. To recover nuclear single-copy gene sequences from the PE reads, an alignment-based method was employed using Read2Tree v0.1.5^60^. A total of 1000 orthologous markers for *Picea glauca* and *Pinus taeda* were obtained from the OMA browser (https://omabrowser.org/oma/export_markers)^61^. Read2Tree was utilized to generate nucleotide sequence alignments using these marker genes. Phylogenetic analyses of each dataset were performed using IQ-TREE2 v2.1.4-beta^62^ with a best-fit model (-m TEST) and 1000 ultrafast bootstrap replicates. ASTRAL v5.7.8^63^ was used for the datasets of 452 nuclear genes. Neighbor-Net was inferred using SplitsTree v4.15.1^64^ with uncorrected *p*-distances and 1000 bootstrap replicates. Three tree topologies were statistically tested for incongruence using approximately unbiased (AU), Shimodaira-Hasegawa (SH), and Kishino-Hasegawa (KH) methods. IQ-TREE2 estimated site likelihoods for each topology on three concatenated datasets and performed AU, SH, and KH tests. Species-level taxonomy follows that of Farjon and Rushforth^41^, except for the recognition of *A. kawakamii*, which is placed in Sect. *Momi* as suggested by phylogenetic studies^3,65^.

### Ethics approval and consent to participate

The experimental studies on the plants, including collection of the material, complied with institutional, national, and international guidelines.

### Supplementary Information

Supplementary Information.

## Data Availability

The datasets supporting the results of this article are included in additional files. Complete plastid and mitochondrial genome sequences are available in GenBank (https://www.ncbi.nlm.nih.gov/nuccore/ON897689, ON897690). DNA sequencing files are available in the NCBI Sequence Read Archive (http://www.ncbi.nlm.nih.gov/sra) under BioProject PRJNA1088239. The phylogenetic datasets generated during the current study are available in the Dryad Digital Repository, (https://doi.org/10.5061/dryad.83bk3j9wh).

## Abbreviations

ETS: External transcribed spacer
ITS: Internal transcribed spacer
MINCs: Mitochondrial DNA of nuclear origin
MIPTs: Mitochondrial DNA of plastid origin
ORF: Open reading frame
LSC: Large single copy
SSC: Small single copy
IR: Inverted repeat
