# Supplementary material for: Complete organelle genomes of Korean fir, Abies koreana and phylogenomics of the gymnosperm genus Abies using nuclear and cytoplasmic DNA sequence data
Source: Sci Rep. 2024 Apr 1;14:7636. doi: 10.1038/s41598-024-58253-x (PMC10985005; doi:10.1038/s41598-024-58253-x)
Supplement: Supplementary file 1 — Supplementary Information. [file 41598_2024_58253_MOESM1_ESM.pdf]

Article title: Complete organelle genomes of Korean fir, *Abies koreana* and phylogenomics of the gymnosperm genus *Abies* using nuclear and cytoplasmic DNA sequence data

Authors: Seongjun Park, Myounghai Kwak, SeonJoo Park

Additional material

Figure S1. Amino acid sequence and transmembrane helices of ORF69 of *Abies koreana*.

Figure S2. Nucleotide and amino acid sequence alignments of the mitochondrial *cox1* gene.

Figure S3. Nucleotide and amino acid sequence alignments of the mitochondrial *rps19* gene.

Figure S4. Structural alignments of nuclear ribosomal DNA regions from nine *Abies* species.

Figure S5. Maximum likelihood tree based on a concatenated alignment from 41 mitochondrial genes and 13 introns of *Abies* species.

Figure S6. Tests for alternative tree topologies using four different datasets.

Figure S7. Phylogenetic networks among the analyzed *Abies* species inferred by SplitsTree4.

Table S1. Predicted repeat pairs in *Abies koreana* mitochondrial genome.

Table S2. plastid-derived DNA segments in *Abies koreana* mitochondrial genome.

Table S3. Summary of putative transposable elements (TEs) in *Abies koreana* mitochondrial genome.

Table S4. Mitochondrial gene clusters among gymnosperms.

Table S5. Putative chimeric ORFs.

Table S6. Summary of editing sites detected in the 41 protein-coding genes of the *Abies koreana* mitogenome.

Table S7. RNA editing in 41 protein-coding genes for *Abies koreana* mitochondrial genome.

Table S8. Predicted repeat pairs in *Abies koreana* plastid genome.

Table S9. Summary of *Abies* plastid genomes.

Table S10. Summary of *Abies* mitochondrial genomes.

Figure S1. Amino acid sequence and transmembrane helices of ORF69 of *Abies koreana*.
A. Amino acid sequences of ORF69. Gray annotation indicate a partial *ccmC* gene. **B**. Schematic of the transmembrane helices of ORF69 as identified by the TMHMM.

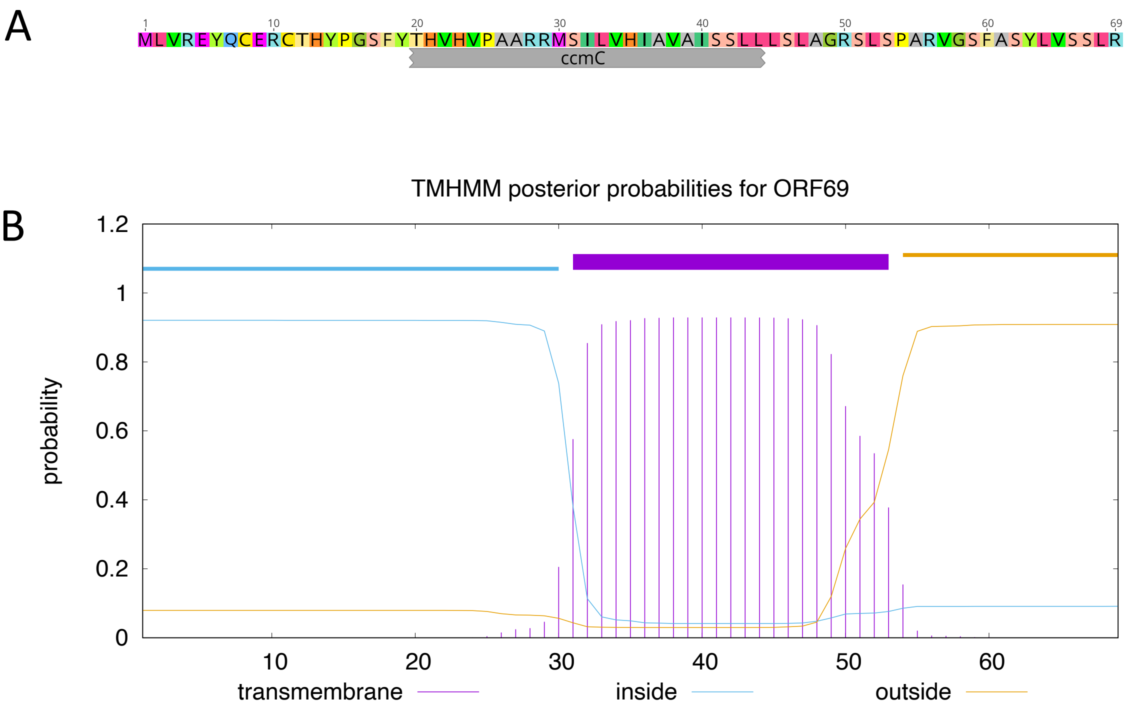

Figure S2. Nucleotide and amino acid sequence alignments of the mitochondrial *cox1* gene. Blue box indicate an internal stop codon created by RNA editing. Red box indicate a new start codon created by RNA editing.

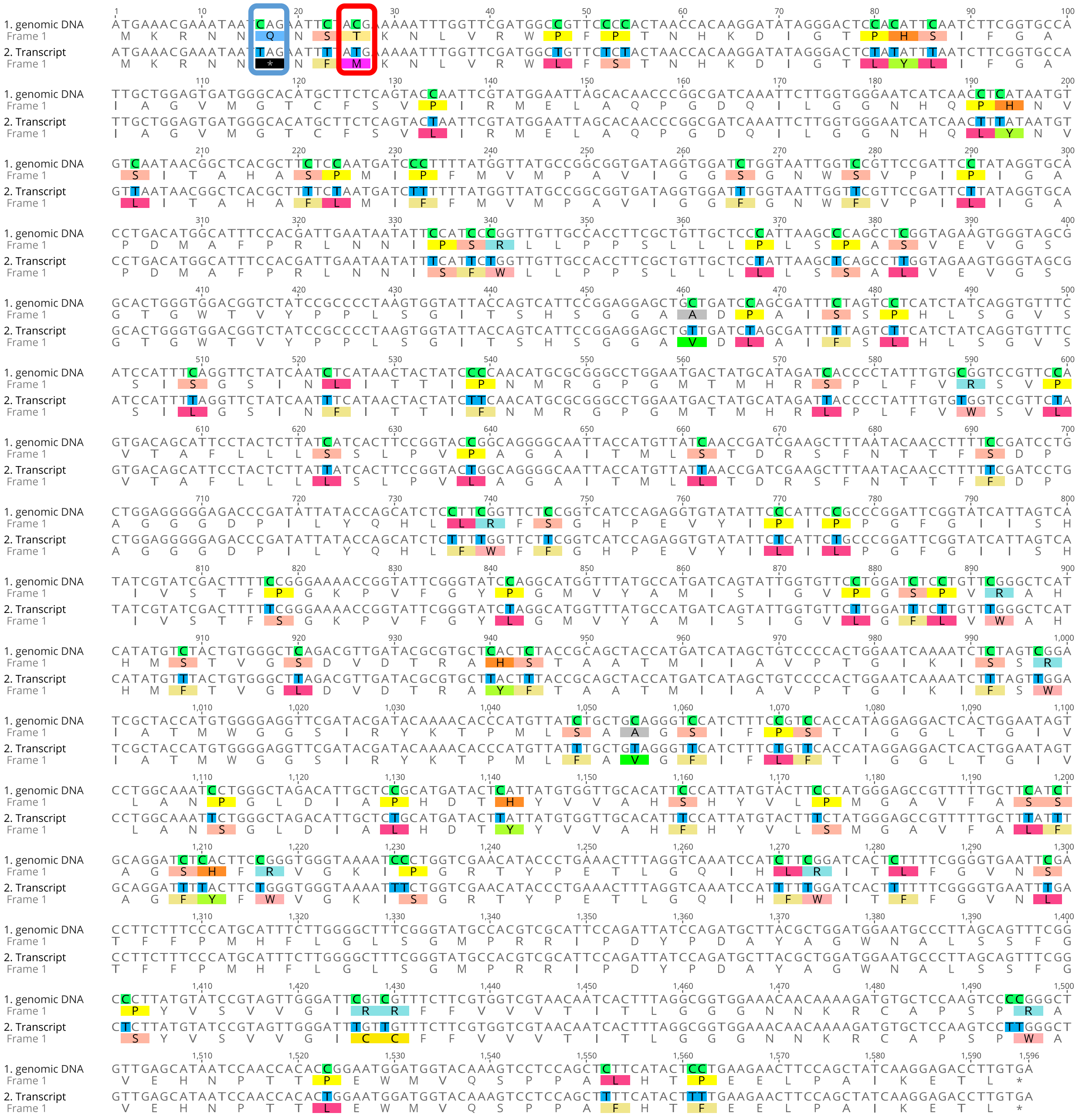

Figure S3. Nucleotide and amino acid sequence alignments of the mitochondrial *rps19* gene.
Red box indicate an alternative start codon created by RNA editing.

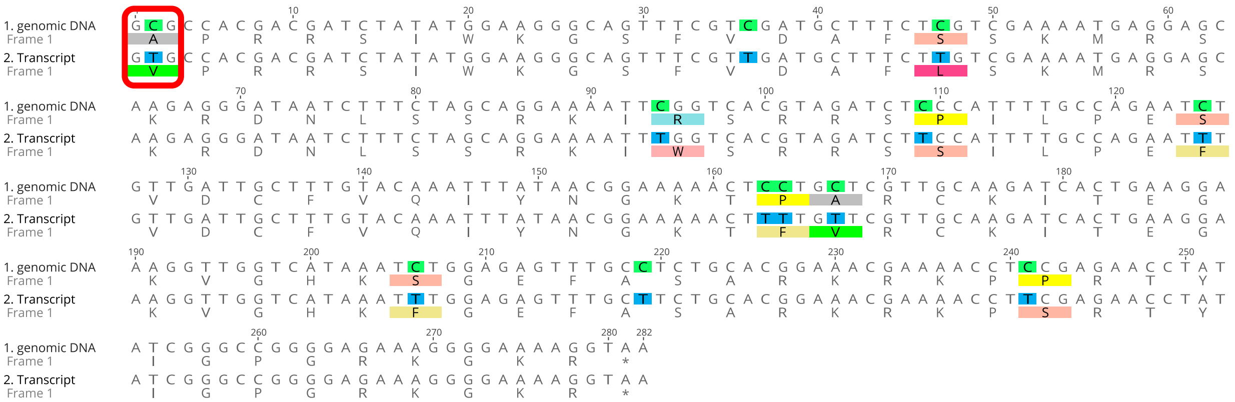

Figure S4. Structural alignments of nuclear ribosomal DNA regions from nine *Abies* species. Neighbor-joining tree was constructed using this dataset. Red boxes indicate a 18S, 5.8S, and 26S rRNA. ASP:*A. spectabilis*, API: *A. pindrow*, AFI: *A. firma*, AKA: *A. kawakamii*, AVE: *A. veitchii*, ANE: *A. nephrolepis*, AKO: *A. koreana*, ASA: *A. sachalinensis*, ASI: *A. sibirica.*

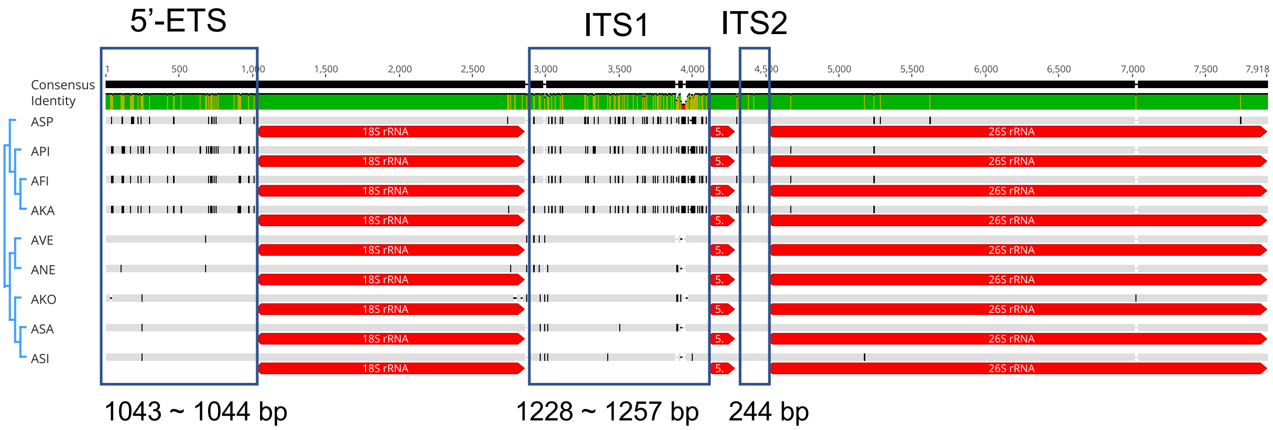

Taxon	Total size (bp)	5'-ETS (bp)	18S rRNA (bp)	ITS1 (bp)	5.8S rRNA (bp)	ITS2 (bp)	26S rRNA
A. kawakamii	7906	1044	1812	1257	162	244	3387
A. nephrolepis	7883	1044	1812	1234	162	244	3387
A. pindrow	7904	1044	1812	1255	162	244	3387
A. sachalinensis	7882	1044	1812	1233	162	244	3387
A. sibirica	7877	1044	1812	1228	162	244	3387
A. spectabilis	7904	1044	1812	1255	162	244	3387
A. veitchii	7878	1044	1812	1229	162	244	3387
A. firma	7905	1044	1812	1256	162	244	3387
A. koreana	7881	1043	1810	1234	162	244	3388

Figure S5. Maximum likelihood tree based on a concatenated alignment from 41 mitochondrial genes and 13 introns of *Abies* species.

The bootstrap value based on 1,000 replicates and > 50% is shown for each node.

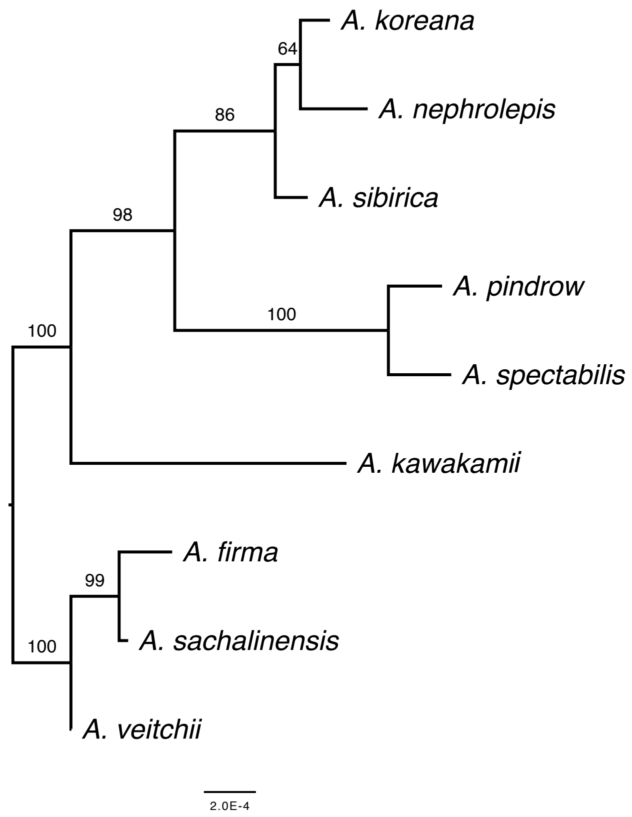

Figure S6. Tests for alternative tree topologies using four different datasets.
AU: *p*-value of approximately unbiased (AU) test, SH: *p*-value of Shimodaira-Hasegawa test, KH: *p*-value of one sided Kishino-Hasegawa test

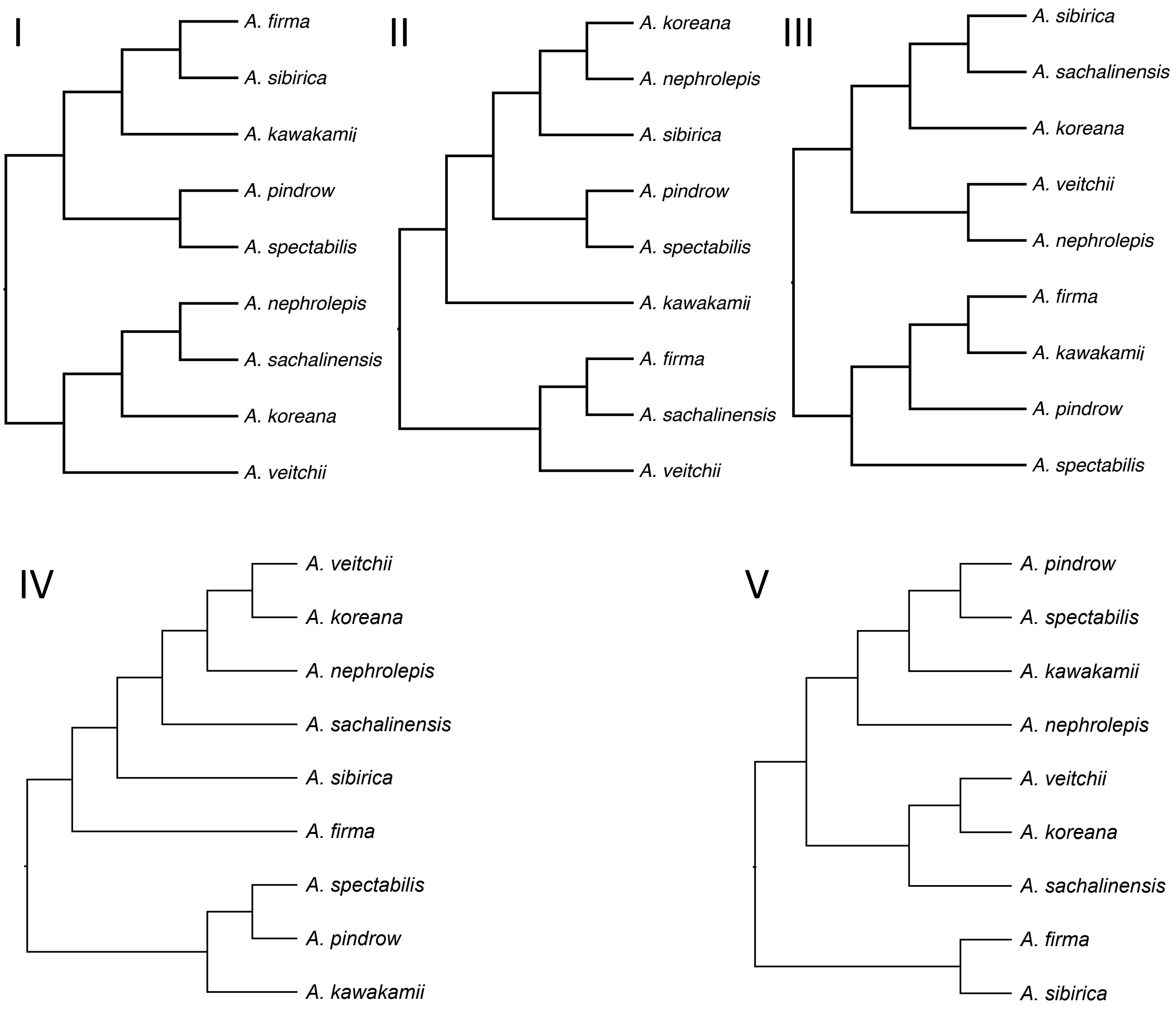

Dataset	Tree topology (AU/SH/KH)				
	I	II	III	IV	V
Whole plastome sequences	1/1/1	0/0/0	0/0/0	0/0/0	0/0/0
41+13 mitochondrial sequences	0/0/0	1/1/1	0/0/0	0/0/0	0/0/0
Nuclear rDNA sequences	0/0/0	0/0/0	1/1/1	0/0/0	0/0/0
452 nuclear sequences	0/0/0	0/0/0	0/0/0	0/0/0	1/1/0.993

Figure S7. Phylogenetic networks among the analyzed *Abies* species inferred by SplitsTree4.

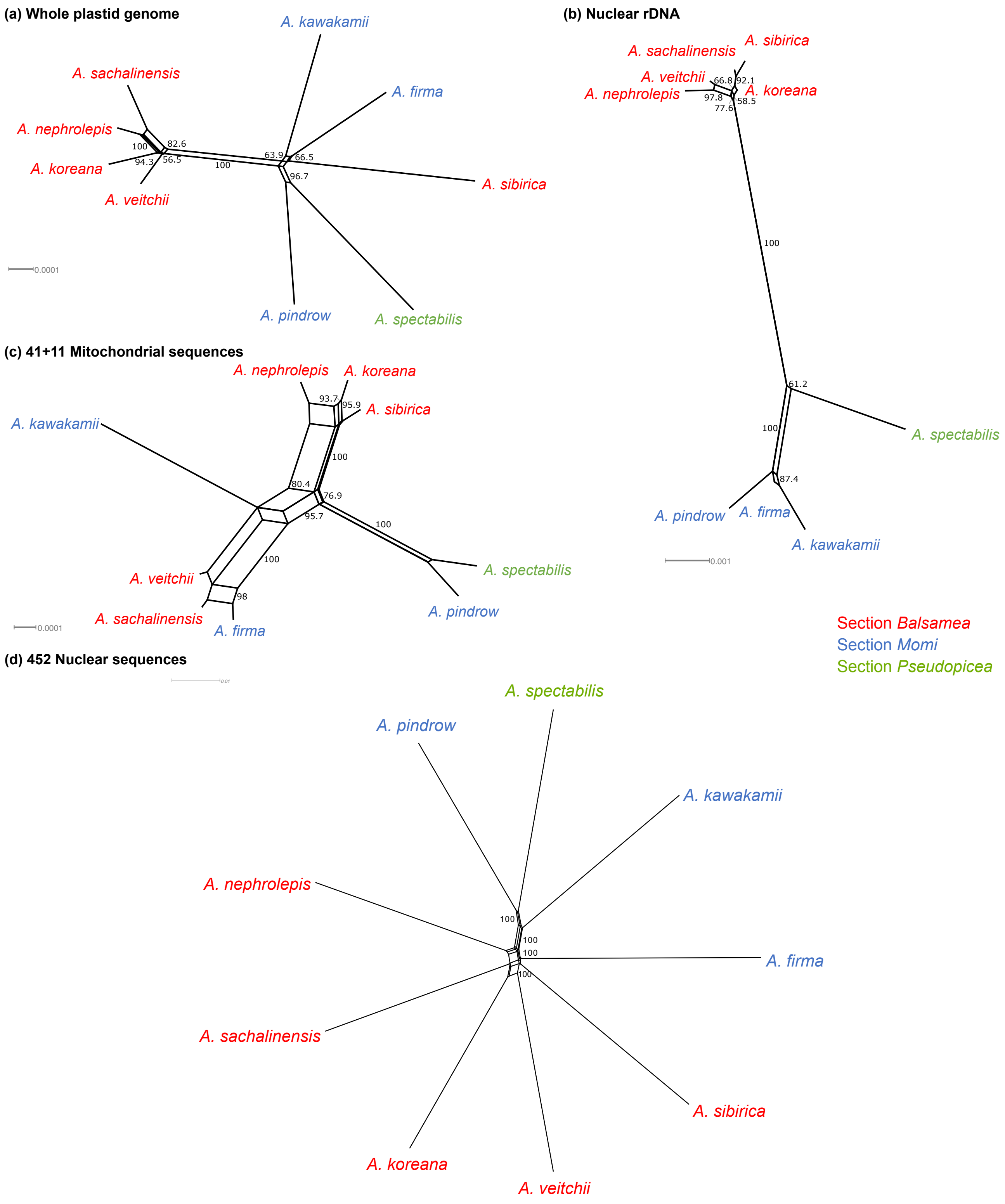

Table S1. Predicted repeat pairs in *Abies koreana* mitochondrial genome.

Repeat	Length	Start	End	Direction	Repeat	Length	Start	End	Direction
Repeat_1	27029	611642	638670	plus	Repeat_306	36	448942	448907	minus
Repeat_1	27029	843862	870890	plus	Repeat_306	36	424477	424512	plus
Repeat_2	23927	594944	618870	plus	Repeat_307	36	371962	371997	plus
Repeat_2	23927	1150877	1174803	plus	Repeat_307	36	909938	909903	minus
Repeat_3	22344	572600	594943	plus	Repeat_308	36	366983	366948	minus
Repeat_3	22344	1128527	1150870	plus	Repeat_308	36	371666	371701	plus
Repeat_4	12115	725642	737756	plus	Repeat_308	36	703276	703241	minus
Repeat_4	12115	1017695	1029809	plus	Repeat_309	36	1046651	1046616	minus
Repeat_5	7414	60048	67461	plus	Repeat_309	36	365500	365535	plus
Repeat_5	7414	251784	244371	minus	Repeat_310	36	353371	353406	plus
Repeat_6	7229	611642	618870	plus	Repeat_310	36	769576	769611	plus
Repeat_6	7229	843862	851090	plus	Repeat_311	36	348814	348849	plus
Repeat_6	7229	1167575	1174803	plus	Repeat_311	36	947739	947704	minus
Repeat_7	5291	737758	743048	plus	Repeat_312	36	641733	641698	minus
Repeat_7	5291	1029811	1035101	plus	Repeat_312	36	346583	346618	plus
Repeat_8	1234	276871	278104	plus	Repeat_313	36	325340	325375	plus
Repeat_8	1234	475135	476368	plus	Repeat_313	36	709092	709127	plus
Repeat_9	1043	278106	279148	plus	Repeat_314	36	325019	325054	plus
Repeat_9	1043	476370	477412	plus	Repeat_314	36	939206	939241	plus
Repeat_10	987	275883	276869	plus	Repeat_315	36	324830	324865	plus
Repeat_10	987	474147	475133	plus	Repeat_315	36	462082	462117	plus
Repeat_11	852	458611	459462	plus	Repeat_315	36	1052083	1052118	plus
Repeat_11	852	1082760	1081909	minus	Repeat_316	36	1016441	1016406	minus
Repeat_12	687	743171	743857	plus	Repeat_316	36	1016478	1016443	minus
Repeat_12	687	1035228	1035914	plus	Repeat_316	36	1042609	1042574	minus
Repeat_13	533	744082	744614	plus	Repeat_316	36	322349	322384	plus
Repeat_13	533	1036162	1036694	plus	Repeat_316	36	324671	324706	plus
Repeat_14	520	666	1185	plus	Repeat_317	36	307038	307073	plus
Repeat_14	520	936042	935523	minus	Repeat_317	36	448300	448335	plus
Repeat_15	516	157214	157729	plus	Repeat_318	36	278695	278730	plus
Repeat_15	516	334666	334151	minus	Repeat_318	36	476959	476994	plus
Repeat_16	341	228965	229305	plus	Repeat_318	36	929518	929483	minus
Repeat_16	341	729283	728943	minus	Repeat_319	36	278500	278535	plus
Repeat_16	341	1021336	1020996	minus	Repeat_319	36	336238	336203	minus
Repeat_17	319	744637	744955	plus	Repeat_319	36	476764	476799	plus
Repeat_17	319	998698	999016	plus	Repeat_320	36	640897	640862	minus
Repeat_18	283	461059	461341	plus	Repeat_320	36	278069	278104	plus
Repeat_18	283	708119	708401	plus	Repeat_320	36	476333	476368	plus
Repeat_19	281	458329	458609	plus	Repeat_321	36	257725	257760	plus
Repeat_19	281	1083042	1082762	minus	Repeat_321	36	371579	371544	minus
Repeat_20	270	24570	24839	plus	Repeat_321	36	619970	620005	plus
Repeat_20	270	922534	922265	minus	Repeat_321	36	743052	743087	plus
Repeat_21	266	829223	829488	plus	Repeat_321	36	773618	773583	minus
Repeat_21	266	979839	979574	minus	Repeat_321	36	852190	852225	plus
Repeat_22	218	334649	334866	plus	Repeat_321	36	1035105	1035140	plus
Repeat_22	218	594935	595152	plus	Repeat_322	36	230815	230850	plus
Repeat_23	209	334658	334866	plus	Repeat_322	36	302271	302306	plus
Repeat_23	209	594944	595152	plus	Repeat_323	36	158155	158190	plus
Repeat_23	209	1150877	1151085	plus	Repeat_323	36	927925	927960	plus
Repeat_24	203	743878	744080	plus	Repeat_324	36	147826	147861	plus
Repeat_24	203	1035958	1036160	plus	Repeat_324	36	311009	310974	minus
Repeat_26	182	461345	461526	plus	Repeat_325	36	1046667	1046632	minus
Repeat_26	182	708411	708592	plus	Repeat_325	36	146233	146268	plus
Repeat_27	180	617463	617642	plus	Repeat_326	36	108082	108117	plus
Repeat_27	180	849683	849862	plus	Repeat_326	36	128237	128202	minus
Repeat_27	180	1016481	1016660	plus	Repeat_326	36	1118046	1118081	plus
Repeat_27	180	1173396	1173575	plus	Repeat_327	36	104901	104936	plus

Repeat_28	161	744616	744776	plus	Repeat_327	36	576133	576168	plus
Repeat_28	161	1036696	1036856	plus	Repeat_327	36	1132060	1132095	plus
Repeat_29	159	676381	676539	plus	Repeat_328	36	58814	58849	plus
Repeat_29	159	810320	810478	plus	Repeat_328	36	961080	961115	plus
Repeat_30	154	969	1122	plus	Repeat_329	36	53858	53893	plus
Repeat_30	154	522161	522008	minus	Repeat_329	36	522645	522680	plus
Repeat_30	154	935739	935586	minus	Repeat_329	36	1101795	1101830	plus
Repeat_31	143	324772	324914	plus	Repeat_330	36	37514	37549	plus
Repeat_31	143	1052025	1052167	plus	Repeat_330	36	257650	257685	plus
Repeat_32	141	1	141	plus	Repeat_330	36	325361	325396	plus
Repeat_32	141	618871	619011	plus	Repeat_330	36	742979	743014	plus
Repeat_32	141	851091	851231	plus	Repeat_330	36	1035032	1035067	plus
Repeat_33	140	744637	744776	plus	Repeat_331	36	427503	427468	minus
Repeat_33	140	998698	998837	plus	Repeat_331	36	490729	490694	minus
Repeat_33	140	1036717	1036856	plus	Repeat_331	36	27336	27371	plus
Repeat_34	137	461728	461864	plus	Repeat_332	36	22921	22956	plus
Repeat_34	137	708819	708955	plus	Repeat_332	36	389854	389819	minus
Repeat_35	127	775348	775222	minus	Repeat_333	35	1078387	1078421	plus
Repeat_35	127	618250	618376	plus	Repeat_333	35	1107221	1107187	minus
Repeat_35	127	850470	850596	plus	Repeat_334	35	946629	946663	plus
Repeat_35	127	1174183	1174309	plus	Repeat_334	35	1105920	1105886	minus
Repeat_36	116	117998	118113	plus	Repeat_335	35	909902	909936	plus
Repeat_36	116	937984	938099	plus	Repeat_335	35	910058	910092	plus
Repeat_37	116	108136	108251	plus	Repeat_336	35	899339	899373	plus
Repeat_37	116	128184	128069	minus	Repeat_336	35	943822	943856	plus
Repeat_38	110	104979	105088	plus	Repeat_337	35	778810	778844	plus
Repeat_38	110	576211	576320	plus	Repeat_337	35	778845	778879	plus
Repeat_38	110	1132138	1132247	plus	Repeat_338	35	717985	718019	plus
Repeat_39	109	379237	379345	plus	Repeat_338	35	1066088	1066122	plus
Repeat_39	109	594800	594908	plus	Repeat_339	35	775791	775757	minus
Repeat_39	109	1150727	1150835	plus	Repeat_339	35	618199	618233	plus
Repeat_40	106	389160	389265	plus	Repeat_339	35	850419	850453	plus
Repeat_40	106	390987	391092	plus	Repeat_339	35	1174132	1174166	plus
Repeat_42	103	586455	586557	plus	Repeat_340	35	257668	257634	minus
Repeat_42	103	726447	726345	minus	Repeat_340	35	560529	560563	plus
Repeat_42	103	1018500	1018398	minus	Repeat_340	35	773673	773707	plus
Repeat_42	103	1142382	1142484	plus	Repeat_341	35	521559	521593	plus
Repeat_43	103	351783	351885	plus	Repeat_341	35	643014	642980	minus
Repeat_43	103	619598	619496	minus	Repeat_342	35	518574	518608	plus
Repeat_43	103	851818	851716	minus	Repeat_342	35	518604	518638	plus
Repeat_44	102	829513	829614	plus	Repeat_343	35	478654	478688	plus
Repeat_44	102	979560	979459	minus	Repeat_343	35	787877	787911	plus
Repeat_45	101	236056	235956	minus	Repeat_344	35	462233	462267	plus
Repeat_45	101	110234	110334	plus	Repeat_344	35	1035160	1035194	plus
Repeat_46	98	322782	322879	plus	Repeat_345	35	462176	462210	plus
Repeat_46	98	814027	814124	plus	Repeat_345	35	619968	620002	plus
Repeat_47	93	427158	427250	plus	Repeat_345	35	743050	743084	plus
Repeat_47	93	462203	462111	minus	Repeat_345	35	852188	852222	plus
Repeat_48	89	146352	146440	plus	Repeat_345	35	1035103	1035137	plus
Repeat_48	89	460060	460148	plus	Repeat_345	35	371581	371547	minus
Repeat_49	88	461627	461714	plus	Repeat_345	35	773620	773586	minus
Repeat_49	88	708723	708810	plus	Repeat_345	35	987306	987272	minus
Repeat_50	88	257243	257330	plus	Repeat_346	35	450923	450957	plus
Repeat_50	88	308497	308584	plus	Repeat_346	35	1013791	1013825	plus
Repeat_50	88	774162	774075	minus	Repeat_347	35	997894	997860	minus
Repeat_51	88	257642	257729	plus	Repeat_347	35	427151	427185	plus
Repeat_51	88	325353	325440	plus	Repeat_348	35	388968	389002	plus
Repeat_52	87	617779	617865	plus	Repeat_348	35	1109566	1109600	plus
Repeat_52	87	849999	850085	plus	Repeat_349	35	371554	371588	plus
Repeat_52	87	1173712	1173798	plus	Repeat_349	35	427158	427192	plus
Repeat_52	87	776360	776274	minus	Repeat_349	35	462203	462169	minus

Repeat_53	87	53329	53415	plus	Repeat_349	35	773593	773627	plus
Repeat_53	87	279050	279136	plus	Repeat_350	35	349274	349308	plus
Repeat_53	87	477314	477400	plus	Repeat_350	35	980996	981030	plus
Repeat_54	86	460708	460793	plus	Repeat_351	35	335118	335152	plus
Repeat_54	86	617912	617997	plus	Repeat_351	35	922064	922098	plus
Repeat_54	86	850132	850217	plus	Repeat_352	35	324880	324914	plus
Repeat_54	86	1173845	1173930	plus	Repeat_352	35	324915	324949	plus
Repeat_55	85	53118	53202	plus	Repeat_352	35	1052133	1052167	plus
Repeat_55	85	278830	278914	plus	Repeat_353	35	309073	309107	plus
Repeat_55	85	477094	477178	plus	Repeat_353	35	815970	816004	plus
Repeat_56	84	446065	445982	minus	Repeat_354	35	257353	257387	plus
Repeat_56	84	308682	308765	plus	Repeat_354	35	308608	308642	plus
Repeat_57	83	254933	255015	plus	Repeat_354	35	446139	446105	minus
Repeat_57	83	925778	925696	minus	Repeat_354	35	1117961	1117995	plus
Repeat_58	82	51721	51802	plus	Repeat_355	35	302307	302341	plus
Repeat_58	82	335932	335851	minus	Repeat_355	35	322006	322040	plus
Repeat_59	81	1003897	1003817	minus	Repeat_355	35	609072	609038	minus
Repeat_59	81	970697	970777	plus	Repeat_355	35	1165005	1164971	minus
Repeat_60	81	476333	476413	plus	Repeat_356	35	446101	446067	minus
Repeat_60	81	640897	640817	minus	Repeat_356	35	257391	257425	plus
Repeat_61	81	52779	52859	plus	Repeat_356	35	308646	308680	plus
Repeat_61	81	413785	413865	plus	Repeat_357	35	230901	230935	plus
Repeat_62	80	923799	923878	plus	Repeat_357	35	230910	230944	plus
Repeat_62	80	1004872	1004951	plus	Repeat_358	35	211951	211985	plus
Repeat_63	79	743050	743128	plus	Repeat_358	35	241483	241517	plus
Repeat_63	79	1035103	1035181	plus	Repeat_359	35	153804	153838	plus
Repeat_64	79	461528	461606	plus	Repeat_359	35	983696	983730	plus
Repeat_64	79	708594	708672	plus	Repeat_360	35	142997	143031	plus
Repeat_65	79	432066	432144	plus	Repeat_360	35	655323	655289	minus
Repeat_65	79	692323	692245	minus	Repeat_361	35	115006	115040	plus
Repeat_66	78	366948	367025	plus	Repeat_361	35	351166	351132	minus
Repeat_66	78	371701	371624	minus	Repeat_362	35	96291	96325	plus
Repeat_67	77	446231	446155	minus	Repeat_362	35	662335	662301	minus
Repeat_67	77	308514	308590	plus	Repeat_363	35	90422	90456	plus
Repeat_68	77	104811	104887	plus	Repeat_363	35	90500	90534	plus
Repeat_68	77	576028	576104	plus	Repeat_364	35	59252	59286	plus
Repeat_68	77	1131955	1132031	plus	Repeat_364	35	147037	147003	minus
Repeat_69	76	618085	618160	plus	Repeat_365	35	56490	56524	plus
Repeat_69	76	775910	775835	minus	Repeat_365	35	711543	711577	plus
Repeat_69	76	850305	850380	plus	Repeat_366	35	55013	55047	plus
Repeat_69	76	1174018	1174093	plus	Repeat_366	35	980831	980865	plus
Repeat_70	75	108630	108704	plus	Repeat_367	35	53859	53893	plus
Repeat_70	75	1083084	1083158	plus	Repeat_367	35	522646	522680	plus
Repeat_71	75	52100	52174	plus	Repeat_367	35	910271	910305	plus
Repeat_71	75	594564	594490	minus	Repeat_367	35	1101796	1101830	plus
Repeat_71	75	1150491	1150417	minus	Repeat_368	35	462185	462151	minus
Repeat_72	74	529	602	plus	Repeat_368	35	53855	53889	plus
Repeat_72	74	619065	619138	plus	Repeat_368	35	427176	427210	plus
Repeat_72	74	851285	851358	plus	Repeat_368	35	522642	522676	plus
Repeat_73	73	773877	773949	plus	Repeat_368	35	773611	773645	plus
Repeat_73	73	972076	972004	minus	Repeat_369	35	53304	53338	plus
Repeat_74	73	257353	257425	plus	Repeat_369	35	521885	521919	plus
Repeat_74	73	308608	308680	plus	Repeat_370	35	755	789	plus
Repeat_75	72	618386	618457	plus	Repeat_370	35	24282	24316	plus
Repeat_75	72	850606	850677	plus	Repeat_370	35	935953	935919	minus
Repeat_75	72	1174319	1174390	plus	Repeat_371	34	1106948	1106981	plus
Repeat_75	72	775212	775141	minus	Repeat_371	34	1118044	1118011	minus
Repeat_76	72	353277	353348	plus	Repeat_372	34	910148	910181	plus
Repeat_76	72	769483	769554	plus	Repeat_372	34	1037819	1037786	minus
Repeat_77	71	460746	460816	plus	Repeat_373	34	789717	789750	plus
Repeat_77	71	707799	707869	plus	Repeat_373	34	1106555	1106588	plus

Repeat_78	71	257260	257330	plus	Repeat_374	34	773664	773697	plus
Repeat_78	71	308514	308584	plus	Repeat_374	34	932628	932661	plus
Repeat_78	71	446231	446161	minus	Repeat_375	34	53858	53891	plus
Repeat_78	71	774145	774075	minus	Repeat_375	34	522645	522678	plus
Repeat_79	71	128260	128190	minus	Repeat_375	34	773614	773647	plus
Repeat_79	71	108059	108129	plus	Repeat_375	34	1101795	1101828	plus
Repeat_80	70	146433	146502	plus	Repeat_376	34	619859	619892	plus
Repeat_80	70	460145	460214	plus	Repeat_376	34	709098	709131	plus
Repeat_81	69	1106286	1106354	plus	Repeat_376	34	852079	852112	plus
Repeat_81	69	1106510	1106578	plus	Repeat_377	34	532923	532956	plus
Repeat_82	69	1003995	1003927	minus	Repeat_377	34	1109522	1109555	plus
Repeat_82	69	970303	970371	plus	Repeat_378	34	462079	462112	plus
Repeat_83	69	51596	51528	minus	Repeat_378	34	774002	773969	minus
Repeat_83	69	24474	24542	plus	Repeat_379	34	460869	460902	plus
Repeat_84	69	600	668	plus	Repeat_379	34	707918	707951	plus
Repeat_84	69	619138	619206	plus	Repeat_380	34	452420	452453	plus
Repeat_84	69	851358	851426	plus	Repeat_380	34	452440	452473	plus
Repeat_85	68	830282	830349	plus	Repeat_381	34	909936	909903	minus
Repeat_85	68	830350	830417	plus	Repeat_381	34	910092	910059	minus
Repeat_86	68	594506	594573	plus	Repeat_381	34	371964	371997	plus
Repeat_86	68	927193	927126	minus	Repeat_382	34	371962	371995	plus
Repeat_86	68	1150433	1150500	plus	Repeat_382	34	773966	773999	plus
Repeat_87	68	148066	148133	plus	Repeat_382	34	909938	909905	minus
Repeat_87	68	310765	310698	minus	Repeat_383	34	347072	347105	plus
Repeat_88	67	460677	460743	plus	Repeat_383	34	729765	729798	plus
Repeat_88	67	707730	707796	plus	Repeat_383	34	1021818	1021851	plus
Repeat_89	67	403285	403351	plus	Repeat_384	34	325011	325044	plus
Repeat_89	67	534692	534758	plus	Repeat_384	34	513860	513893	plus
Repeat_90	67	308771	308837	plus	Repeat_385	34	324827	324860	plus
Repeat_90	67	445975	445909	minus	Repeat_385	34	909902	909935	plus
Repeat_91	67	55134	55200	plus	Repeat_385	34	910058	910091	plus
Repeat_91	67	432201	432267	plus	Repeat_385	34	1052080	1052113	plus
Repeat_92	66	388050	388115	plus	Repeat_386	34	322588	322621	plus
Repeat_92	66	1114236	1114301	plus	Repeat_386	34	448732	448765	plus
Repeat_93	66	158434	158499	plus	Repeat_387	34	309406	309439	plus
Repeat_93	66	996764	996699	minus	Repeat_387	34	1042939	1042972	plus
Repeat_94	66	104679	104744	plus	Repeat_388	34	289125	289158	plus
Repeat_94	66	575889	575954	plus	Repeat_388	34	533077	533110	plus
Repeat_94	66	1131816	1131881	plus	Repeat_389	34	257631	257664	plus
Repeat_95	66	336280	336215	minus	Repeat_389	34	726076	726109	plus
Repeat_95	66	25099	25164	plus	Repeat_389	34	1018129	1018162	plus
Repeat_96	65	971827	971763	minus	Repeat_389	34	371801	371768	minus
Repeat_96	65	685193	685257	plus	Repeat_389	34	773710	773677	minus
Repeat_97	65	427189	427253	plus	Repeat_390	34	257629	257662	plus
Repeat_97	65	743046	742982	minus	Repeat_390	34	371803	371770	minus
Repeat_97	65	1035099	1035035	minus	Repeat_390	34	702852	702819	minus
Repeat_98	65	351646	351710	plus	Repeat_390	34	773712	773679	minus
Repeat_98	65	619725	619661	minus	Repeat_391	34	257621	257654	plus
Repeat_98	65	851945	851881	minus	Repeat_391	34	910053	910086	plus
Repeat_99	65	117803	117867	plus	Repeat_391	34	371811	371778	minus
Repeat_99	65	929631	929695	plus	Repeat_392	34	255487	255520	plus
Repeat_100	64	162661	162724	plus	Repeat_392	34	307117	307150	plus
Repeat_100	64	805017	804954	minus	Repeat_393	34	335317	335284	minus
Repeat_101	63	388694	388756	plus	Repeat_393	34	175578	175611	plus
Repeat_101	63	390769	390831	plus	Repeat_394	34	153802	153835	plus
Repeat_102	63	348117	348179	plus	Repeat_394	34	325340	325373	plus
Repeat_102	63	1107124	1107062	minus	Repeat_394	34	709092	709125	plus
Repeat_103	63	255396	255458	plus	Repeat_395	34	146146	146179	plus
Repeat_103	63	304310	304372	plus	Repeat_395	34	390945	390978	plus
Repeat_104	62	741817	741878	plus	Repeat_396	34	141026	141059	plus
Repeat_104	62	980531	980592	plus	Repeat_396	34	366535	366568	plus

Repeat_104	62	1033870	1033931	plus	Repeat_397	34	1043799	1043766	minus
Repeat_105	62	427250	427189	minus	Repeat_397	34	137828	137861	plus
Repeat_105	62	462111	462172	plus	Repeat_398	34	128026	128059	plus
Repeat_105	62	742985	743046	plus	Repeat_398	34	1078389	1078422	plus
Repeat_105	62	1035038	1035099	plus	Repeat_399	34	1078778	1078745	minus
Repeat_106	62	162604	162665	plus	Repeat_399	34	127935	127968	plus
Repeat_106	62	805072	805011	minus	Repeat_400	34	111606	111639	plus
Repeat_107	61	967856	967916	plus	Repeat_400	34	1017552	1017519	minus
Repeat_107	61	967910	967970	plus	Repeat_401	34	53856	53889	plus
Repeat_108	61	618025	618085	plus	Repeat_401	34	349047	349080	plus
Repeat_108	61	707870	707930	plus	Repeat_401	34	427177	427210	plus
Repeat_108	61	850245	850305	plus	Repeat_401	34	462184	462151	minus
Repeat_108	61	1173958	1174018	plus	Repeat_401	34	522643	522676	plus
Repeat_109	61	211984	212044	plus	Repeat_401	34	773612	773645	plus
Repeat_109	61	241515	241575	plus	Repeat_402	34	53063	53096	plus
Repeat_110	60	773645	773586	minus	Repeat_402	34	106689	106722	plus
Repeat_110	60	462151	462210	plus	Repeat_403	34	52275	52308	plus
Repeat_111	60	228742	228801	plus	Repeat_403	34	927372	927405	plus
Repeat_111	60	419841	419900	plus	Repeat_404	34	44625	44658	plus
Repeat_112	60	127966	128025	plus	Repeat_404	34	907652	907619	minus
Repeat_112	60	1078742	1078683	minus	Repeat_405	34	37514	37547	plus
Repeat_113	60	51472	51531	plus	Repeat_405	34	257650	257683	plus
Repeat_113	60	477501	477560	plus	Repeat_405	34	325361	325394	plus
Repeat_114	59	943867	943925	plus	Repeat_405	34	513938	513971	plus
Repeat_114	59	963887	963829	minus	Repeat_405	34	742979	743012	plus
Repeat_115	59	366925	366983	plus	Repeat_405	34	1035032	1035065	plus
Repeat_115	59	703218	703276	plus	Repeat_406	33	1005168	1005200	plus
Repeat_116	59	619815	619757	minus	Repeat_406	33	1005219	1005251	plus
Repeat_116	59	852035	851977	minus	Repeat_407	33	935047	935079	plus
Repeat_116	59	351556	351614	plus	Repeat_407	33	935235	935267	plus
Repeat_117	59	52100	52158	plus	Repeat_408	33	827458	827490	plus
Repeat_117	59	927135	927193	plus	Repeat_408	33	827540	827572	plus
Repeat_117	59	594564	594506	minus	Repeat_409	33	53859	53891	plus
Repeat_117	59	1150491	1150433	minus	Repeat_409	33	522646	522678	plus
Repeat_118	58	476343	476400	plus	Repeat_409	33	773615	773647	plus
Repeat_118	58	640887	640830	minus	Repeat_409	33	910271	910303	plus
Repeat_118	58	765930	765873	minus	Repeat_409	33	1101796	1101828	plus
Repeat_119	58	371526	371583	plus	Repeat_410	33	742312	742344	plus
Repeat_119	58	1035158	1035101	minus	Repeat_410	33	980783	980815	plus
Repeat_120	58	353722	353779	plus	Repeat_410	33	1034365	1034397	plus
Repeat_120	58	1079752	1079695	minus	Repeat_411	33	594574	594606	plus
Repeat_121	57	910259	910203	minus	Repeat_411	33	1150501	1150533	plus
Repeat_121	57	462193	462249	plus	Repeat_411	33	927116	927084	minus
Repeat_122	57	365811	365867	plus	Repeat_412	33	513651	513683	plus
Repeat_122	57	927491	927547	plus	Repeat_412	33	591508	591540	plus
Repeat_123	57	153769	153825	plus	Repeat_412	33	1147435	1147467	plus
Repeat_123	57	702944	703000	plus	Repeat_413	33	491932	491964	plus
Repeat_124	57	51567	51623	plus	Repeat_413	33	531891	531859	minus
Repeat_124	57	336086	336030	minus	Repeat_414	33	460783	460815	plus
Repeat_125	56	1016406	1016461	plus	Repeat_414	33	707836	707868	plus
Repeat_125	56	1016443	1016498	plus	Repeat_414	33	882876	882908	plus
Repeat_126	56	743105	743050	minus	Repeat_415	33	450923	450955	plus
Repeat_126	56	1035158	1035103	minus	Repeat_415	33	539393	539361	minus
Repeat_126	56	371526	371581	plus	Repeat_415	33	1013791	1013823	plus
Repeat_127	56	462184	462129	minus	Repeat_416	33	448441	448473	plus
Repeat_127	56	349047	349102	plus	Repeat_416	33	501756	501724	minus
Repeat_127	56	427177	427232	plus	Repeat_417	33	430969	431001	plus
Repeat_128	56	308198	308253	plus	Repeat_417	33	484610	484578	minus
Repeat_128	56	761357	761302	minus	Repeat_418	33	388943	388975	plus
Repeat_129	55	590291	590345	plus	Repeat_418	33	389255	389223	minus
Repeat_129	55	977081	977135	plus	Repeat_418	33	391082	391050	minus

Repeat_129	55	1146218	1146272	plus	Repeat_419	33	388068	388100	plus
Repeat_130	54	162439	162492	plus	Repeat_419	33	797879	797847	minus
Repeat_130	54	805194	805141	minus	Repeat_419	33	1114254	1114286	plus
Repeat_131	54	104624	104677	plus	Repeat_420	33	972180	972148	minus
Repeat_131	54	575834	575887	plus	Repeat_420	33	371750	371782	plus
Repeat_131	54	1131761	1131814	plus	Repeat_421	33	351887	351919	plus
Repeat_132	54	85487	85540	plus	Repeat_421	33	619494	619462	minus
Repeat_132	54	799666	799719	plus	Repeat_421	33	851714	851682	minus
Repeat_133	54	53517	53570	plus	Repeat_422	33	347038	347070	plus
Repeat_133	54	593334	593281	minus	Repeat_422	33	729731	729763	plus
Repeat_133	54	1149261	1149208	minus	Repeat_422	33	1021784	1021816	plus
Repeat_134	53	594595	594647	plus	Repeat_423	33	334864	334896	plus
Repeat_134	53	927089	927037	minus	Repeat_423	33	593495	593527	plus
Repeat_134	53	1150522	1150574	plus	Repeat_423	33	1149422	1149454	plus
Repeat_135	53	427158	427210	plus	Repeat_424	33	330342	330374	plus
Repeat_135	53	773593	773645	plus	Repeat_424	33	1072234	1072202	minus
Repeat_135	53	462203	462151	minus	Repeat_425	33	325416	325448	plus
Repeat_136	53	403405	403457	plus	Repeat_425	33	427201	427169	minus
Repeat_136	53	534809	534861	plus	Repeat_425	33	462160	462192	plus
Repeat_137	53	388046	388098	plus	Repeat_425	33	773636	773604	minus
Repeat_137	53	557493	557545	plus	Repeat_426	33	324828	324860	plus
Repeat_138	53	51802	51854	plus	Repeat_426	33	371997	371965	minus
Repeat_138	53	923184	923132	minus	Repeat_426	33	909903	909935	plus
Repeat_139	53	51616	51668	plus	Repeat_426	33	910059	910091	plus
Repeat_139	53	336033	335981	minus	Repeat_426	33	1052081	1052113	plus
Repeat_140	52	807250	807301	plus	Repeat_427	33	322461	322493	plus
Repeat_140	52	1101772	1101721	minus	Repeat_427	33	666822	666854	plus
Repeat_141	52	776997	776946	minus	Repeat_428	33	308985	309017	plus
Repeat_141	52	591842	591893	plus	Repeat_428	33	427066	427098	plus
Repeat_141	52	1147769	1147820	plus	Repeat_429	33	257725	257757	plus
Repeat_142	52	256712	256763	plus	Repeat_429	33	462178	462210	plus
Repeat_142	52	736535	736484	minus	Repeat_429	33	619970	620002	plus
Repeat_142	52	1028588	1028537	minus	Repeat_429	33	743052	743084	plus
Repeat_143	52	193430	193481	plus	Repeat_429	33	852190	852222	plus
Repeat_143	52	958231	958180	minus	Repeat_429	33	1035105	1035137	plus
Repeat_144	52	175489	175540	plus	Repeat_429	33	371579	371547	minus
Repeat_144	52	335399	335348	minus	Repeat_429	33	773618	773586	minus
Repeat_145	52	778454	778403	minus	Repeat_429	33	987304	987272	minus
Repeat_145	52	54253	54304	plus	Repeat_430	33	255301	255333	plus
Repeat_146	52	53843	53894	plus	Repeat_430	33	304214	304246	plus
Repeat_146	52	522630	522681	plus	Repeat_431	33	210734	210766	plus
Repeat_147	51	807303	807353	plus	Repeat_431	33	235613	235645	plus
Repeat_147	51	1101719	1101669	minus	Repeat_432	33	193549	193581	plus
Repeat_148	51	774322	774372	plus	Repeat_432	33	958087	958055	minus
Repeat_148	51	1117945	1117895	minus	Repeat_433	33	173820	173852	plus
Repeat_149	51	460795	460845	plus	Repeat_433	33	620586	620618	plus
Repeat_149	51	617999	618049	plus	Repeat_433	33	852806	852838	plus
Repeat_149	51	850219	850269	plus	Repeat_434	33	151857	151889	plus
Repeat_149	51	1173932	1173982	plus	Repeat_434	33	1013769	1013737	minus
Repeat_150	51	460625	460675	plus	Repeat_435	33	147293	147325	plus
Repeat_150	51	707678	707728	plus	Repeat_435	33	971514	971546	plus
Repeat_151	51	403565	403615	plus	Repeat_436	33	108514	108546	plus
Repeat_151	51	535010	535060	plus	Repeat_436	33	127565	127533	minus
Repeat_152	51	322366	322416	plus	Repeat_437	33	56352	56384	plus
Repeat_152	51	322398	322448	plus	Repeat_437	33	450557	450525	minus
Repeat_153	51	277716	277766	plus	Repeat_438	33	53034	53066	plus
Repeat_153	51	277757	277807	plus	Repeat_438	33	106659	106691	plus
Repeat_153	51	475980	476030	plus	Repeat_439	33	37517	37549	plus
Repeat_153	51	476021	476071	plus	Repeat_439	33	257653	257685	plus
Repeat_154	51	153785	153835	plus	Repeat_439	33	325364	325396	plus
Repeat_154	51	325323	325373	plus	Repeat_439	33	742982	743014	plus

Repeat_155	51	65984	66034	plus	Repeat_439	33	1035035	1035067	plus
Repeat_155	51	245848	245798	minus	Repeat_439	33	427253	427221	minus
Repeat_155	51	308893	308943	plus	Repeat_440	33	1185	1217	plus
Repeat_156	50	932860	932909	plus	Repeat_440	33	128026	128058	plus
Repeat_156	50	932957	933006	plus	Repeat_440	33	1078389	1078421	plus
Repeat_157	50	966429	966380	minus	Repeat_440	33	1107219	1107187	minus
Repeat_157	50	793197	793246	plus	Repeat_441	32	963816	963847	plus
Repeat_158	50	650662	650711	plus	Repeat_441	32	1112780	1112811	plus
Repeat_158	50	653212	653261	plus	Repeat_442	32	883494	883525	plus
Repeat_159	50	348691	348740	plus	Repeat_442	32	929283	929314	plus
Repeat_159	50	667929	667880	minus	Repeat_443	32	371964	371995	plus
Repeat_160	50	246079	246030	minus	Repeat_443	32	773968	773999	plus
Repeat_160	50	65753	65802	plus	Repeat_443	32	909936	909905	minus
Repeat_160	50	591595	591644	plus	Repeat_443	32	910092	910061	minus
Repeat_160	50	1147522	1147571	plus	Repeat_444	32	722929	722960	plus
Repeat_161	49	720791	720839	plus	Repeat_444	32	925712	925681	minus
Repeat_161	49	722594	722642	plus	Repeat_445	32	718143	718174	plus
Repeat_162	49	388050	388098	plus	Repeat_445	32	927299	927330	plus
Repeat_162	49	557497	557545	plus	Repeat_446	32	257662	257631	minus
Repeat_162	49	1114236	1114284	plus	Repeat_446	32	371770	371801	plus
Repeat_163	49	460912	460960	plus	Repeat_446	32	702819	702850	plus
Repeat_163	49	707952	708000	plus	Repeat_446	32	726107	726076	minus
Repeat_164	49	815774	815726	minus	Repeat_446	32	773679	773710	plus
Repeat_164	49	448866	448914	plus	Repeat_446	32	1018160	1018129	minus
Repeat_165	49	388758	388806	plus	Repeat_447	32	695023	695054	plus
Repeat_165	49	390833	390881	plus	Repeat_447	32	758435	758404	minus
Repeat_166	49	231721	231769	plus	Repeat_448	32	619791	619822	plus
Repeat_166	49	310320	310368	plus	Repeat_448	32	729366	729335	minus
Repeat_167	48	460746	460793	plus	Repeat_448	32	852011	852042	plus
Repeat_167	48	617950	617997	plus	Repeat_448	32	1021419	1021388	minus
Repeat_167	48	707799	707846	plus	Repeat_449	32	602203	602234	plus
Repeat_167	48	850170	850217	plus	Repeat_449	32	602369	602400	plus
Repeat_167	48	1173883	1173930	plus	Repeat_449	32	1158136	1158167	plus
Repeat_168	48	324880	324927	plus	Repeat_449	32	1158302	1158333	plus
Repeat_168	48	324915	324962	plus	Repeat_450	32	405316	405347	plus
Repeat_169	48	158088	158135	plus	Repeat_450	32	901867	901836	minus
Repeat_169	48	278320	278367	plus	Repeat_451	32	371967	371998	plus
Repeat_169	48	476584	476631	plus	Repeat_451	32	761199	761230	plus
Repeat_170	48	111556	111603	plus	Repeat_452	32	53858	53889	plus
Repeat_170	48	1017603	1017556	minus	Repeat_452	32	349049	349080	plus
Repeat_171	47	967281	967327	plus	Repeat_452	32	427179	427210	plus
Repeat_171	47	1083135	1083181	plus	Repeat_452	32	462182	462151	minus
Repeat_172	47	460273	460227	minus	Repeat_452	32	522645	522676	plus
Repeat_172	47	458089	458135	plus	Repeat_452	32	773614	773645	plus
Repeat_173	47	325275	325321	plus	Repeat_452	32	1101795	1101826	plus
Repeat_173	47	983629	983675	plus	Repeat_453	32	153804	153835	plus
Repeat_174	47	257648	257694	plus	Repeat_453	32	325342	325373	plus
Repeat_174	47	325359	325405	plus	Repeat_453	32	709094	709125	plus
Repeat_174	47	742977	743023	plus	Repeat_453	32	983696	983727	plus
Repeat_174	47	1035030	1035076	plus	Repeat_454	32	324762	324793	plus
Repeat_175	47	147484	147530	plus	Repeat_454	32	909897	909866	minus
Repeat_175	47	971715	971761	plus	Repeat_455	32	288991	289022	plus
Repeat_176	47	146084	146130	plus	Repeat_455	32	786963	786994	plus
Repeat_176	47	1042570	1042524	minus	Repeat_456	32	276353	276384	plus
Repeat_177	46	970207	970252	plus	Repeat_456	32	474617	474648	plus
Repeat_177	46	1004089	1004044	minus	Repeat_456	32	808757	808726	minus
Repeat_178	46	689599	689644	plus	Repeat_457	32	235779	235810	plus
Repeat_178	46	1043064	1043019	minus	Repeat_457	32	333653	333622	minus
Repeat_179	46	591892	591937	plus	Repeat_458	32	229536	229567	plus
Repeat_179	46	776942	776897	minus	Repeat_458	32	230841	230872	plus
Repeat_179	46	1147819	1147864	plus	Repeat_459	32	200787	200818	plus

Repeat_180	46	212045	212090	plus	Repeat_459	32	546724	546755	plus
Repeat_180	46	212121	212166	plus	Repeat_460	32	175539	175570	plus
Repeat_181	46	163146	163191	plus	Repeat_460	32	335353	335322	minus
Repeat_181	46	163216	163261	plus	Repeat_461	32	164123	164154	plus
Repeat_182	46	51810	51855	plus	Repeat_461	32	629980	629949	minus
Repeat_182	46	335846	335801	minus	Repeat_461	32	862200	862169	minus
Repeat_183	46	24540	24585	plus	Repeat_462	32	162645	162676	plus
Repeat_183	46	335540	335495	minus	Repeat_462	32	682562	682593	plus
Repeat_184	45	776794	776838	plus	Repeat_463	32	153751	153782	plus
Repeat_184	45	813887	813931	plus	Repeat_463	32	325289	325320	plus
Repeat_185	45	371544	371588	plus	Repeat_463	32	983643	983674	plus
Repeat_185	45	773583	773627	plus	Repeat_464	32	127464	127495	plus
Repeat_186	45	51854	51810	minus	Repeat_464	32	967211	967180	minus
Repeat_186	45	335802	335846	plus	Repeat_465	32	108083	108114	plus
Repeat_186	45	923132	923176	plus	Repeat_465	32	128236	128205	minus
Repeat_187	45	324666	324710	plus	Repeat_465	32	590987	590956	minus
Repeat_187	45	1016483	1016439	minus	Repeat_465	32	1118047	1118078	plus
Repeat_188	45	324625	324669	plus	Repeat_465	32	1146914	1146883	minus
Repeat_188	45	426975	427019	plus	Repeat_466	32	67262	67293	plus
Repeat_188	45	818095	818139	plus	Repeat_466	32	244570	244539	minus
Repeat_189	45	162288	162332	plus	Repeat_466	32	647765	647734	minus
Repeat_189	45	805340	805296	minus	Repeat_467	32	66036	66067	plus
Repeat_190	45	246119	246075	minus	Repeat_467	32	245796	245765	minus
Repeat_190	45	65713	65757	plus	Repeat_467	32	324827	324858	plus
Repeat_190	45	513673	513717	plus	Repeat_467	32	909902	909933	plus
Repeat_191	45	58768	58812	plus	Repeat_467	32	910058	910089	plus
Repeat_191	45	961034	961078	plus	Repeat_467	32	1052080	1052111	plus
Repeat_192	45	27336	27380	plus	Repeat_468	32	56365	56396	plus
Repeat_192	45	427503	427459	minus	Repeat_468	32	815735	815704	minus
Repeat_193	44	471823	471866	plus	Repeat_469	32	52067	52098	plus
Repeat_193	44	471845	471888	plus	Repeat_469	32	927106	927137	plus
Repeat_194	44	349059	349102	plus	Repeat_470	32	25110	25141	plus
Repeat_194	44	427189	427232	plus	Repeat_470	32	116438	116407	minus
Repeat_194	44	462172	462129	minus	Repeat_470	32	336269	336238	minus
Repeat_194	44	743046	743003	minus	Repeat_471	31	1080350	1080380	plus
Repeat_194	44	1035099	1035056	minus	Repeat_471	31	1117554	1117524	minus
Repeat_195	44	302208	302251	plus	Repeat_472	31	1070889	1070919	plus
Repeat_195	44	721141	721098	minus	Repeat_472	31	1070952	1070982	plus
Repeat_196	44	297460	297503	plus	Repeat_473	31	1014530	1014560	plus
Repeat_196	44	297487	297530	plus	Repeat_473	31	1117735	1117765	plus
Repeat_197	44	278106	278149	plus	Repeat_474	31	981072	981102	plus
Repeat_197	44	476370	476413	plus	Repeat_474	31	1017228	1017258	plus
Repeat_197	44	640860	640817	minus	Repeat_475	31	970254	970284	plus
Repeat_198	44	257621	257664	plus	Repeat_475	31	1004042	1004012	minus
Repeat_198	44	371811	371768	minus	Repeat_476	31	883524	883494	minus
Repeat_199	44	255348	255391	plus	Repeat_476	31	929313	929283	minus
Repeat_199	44	304266	304309	plus	Repeat_476	31	922051	922081	plus
Repeat_200	44	193491	193534	plus	Repeat_477	31	726055	726085	plus
Repeat_200	44	958153	958110	minus	Repeat_477	31	971138	971168	plus
Repeat_201	44	153701	153744	plus	Repeat_477	31	1018108	1018138	plus
Repeat_201	44	983593	983636	plus	Repeat_478	31	703560	703590	plus
Repeat_202	44	147863	147906	plus	Repeat_478	31	703611	703641	plus
Repeat_202	44	310972	310929	minus	Repeat_479	31	619874	619904	plus
Repeat_203	44	108532	108575	plus	Repeat_479	31	852094	852124	plus
Repeat_203	44	967124	967167	plus	Repeat_479	31	972148	972178	plus
Repeat_204	44	51863	51906	plus	Repeat_479	31	371782	371752	minus
Repeat_204	44	923007	922964	minus	Repeat_480	31	577898	577928	plus
Repeat_205	43	943829	943871	plus	Repeat_480	31	577911	577941	plus
Repeat_205	43	953012	953054	plus	Repeat_480	31	1133825	1133855	plus
Repeat_206	43	743127	743169	plus	Repeat_480	31	1133838	1133868	plus
Repeat_206	43	1035184	1035226	plus	Repeat_481	31	573889	573919	plus

Repeat_207	43	497969	498011	plus	Repeat_481	31	577612	577642	plus
Repeat_207	43	981207	981249	plus	Repeat_481	31	1129816	1129846	plus
Repeat_208	43	325352	325394	plus	Repeat_481	31	1133539	1133569	plus
Repeat_208	43	513929	513971	plus	Repeat_482	31	388068	388098	plus
Repeat_209	43	302301	302343	plus	Repeat_482	31	557515	557545	plus
Repeat_209	43	322000	322042	plus	Repeat_482	31	797879	797849	minus
Repeat_210	43	302277	302319	plus	Repeat_482	31	1114254	1114284	plus
Repeat_210	43	721139	721097	minus	Repeat_483	31	518957	518987	plus
Repeat_211	43	257626	257668	plus	Repeat_483	31	518980	519010	plus
Repeat_211	43	773715	773673	minus	Repeat_484	31	445868	445898	plus
Repeat_212	43	186205	186247	plus	Repeat_484	31	462015	461985	minus
Repeat_212	43	1072411	1072453	plus	Repeat_485	31	37547	37517	minus
Repeat_213	43	113199	113241	plus	Repeat_485	31	257683	257653	minus
Repeat_213	43	1042689	1042731	plus	Repeat_485	31	325394	325364	minus
Repeat_214	43	1031	1073	plus	Repeat_485	31	513971	513941	minus
Repeat_214	43	429908	429866	minus	Repeat_485	31	743012	742982	minus
Repeat_214	43	522099	522057	minus	Repeat_485	31	1035065	1035035	minus
Repeat_214	43	935677	935635	minus	Repeat_485	31	427223	427253	plus
Repeat_215	42	970779	970820	plus	Repeat_486	31	419964	419994	plus
Repeat_215	42	1003815	1003774	minus	Repeat_486	31	1017457	1017487	plus
Repeat_216	42	460039	460080	plus	Repeat_487	31	324860	324830	minus
Repeat_216	42	1109623	1109664	plus	Repeat_487	31	371965	371995	plus
Repeat_217	42	371547	371588	plus	Repeat_487	31	462112	462082	minus
Repeat_217	42	462210	462169	minus	Repeat_487	31	773969	773999	plus
Repeat_217	42	773586	773627	plus	Repeat_487	31	909935	909905	minus
Repeat_218	42	929128	929087	minus	Repeat_487	31	910091	910061	minus
Repeat_218	42	334058	334099	plus	Repeat_487	31	1052113	1052083	minus
Repeat_219	42	721139	721098	minus	Repeat_488	31	257664	257634	minus
Repeat_219	42	302210	302251	plus	Repeat_488	31	371768	371798	plus
Repeat_219	42	302277	302318	plus	Repeat_488	31	560533	560563	plus
Repeat_220	42	257653	257694	plus	Repeat_488	31	726109	726079	minus
Repeat_220	42	325364	325405	plus	Repeat_488	31	773677	773707	plus
Repeat_220	42	742982	743023	plus	Repeat_488	31	1018162	1018132	minus
Repeat_220	42	1035035	1035076	plus	Repeat_489	31	352989	353019	plus
Repeat_220	42	427253	427212	minus	Repeat_489	31	608228	608198	minus
Repeat_221	42	257642	257683	plus	Repeat_489	31	1164161	1164131	minus
Repeat_221	42	325353	325394	plus	Repeat_490	31	462181	462151	minus
Repeat_221	42	513930	513971	plus	Repeat_490	31	53859	53889	plus
Repeat_222	42	230821	230862	plus	Repeat_490	31	349050	349080	plus
Repeat_222	42	1118163	1118122	minus	Repeat_490	31	427180	427210	plus
Repeat_223	42	141096	141137	plus	Repeat_490	31	522646	522676	plus
Repeat_223	42	366605	366646	plus	Repeat_490	31	773615	773645	plus
Repeat_224	42	106453	106494	plus	Repeat_490	31	910271	910301	plus
Repeat_224	42	513590	513549	minus	Repeat_490	31	1101796	1101826	plus
Repeat_225	42	85544	85585	plus	Repeat_491	31	348894	348924	plus
Repeat_225	42	799723	799764	plus	Repeat_491	31	668033	668003	minus
Repeat_226	42	24624	24665	plus	Repeat_492	31	324880	324910	plus
Repeat_226	42	922480	922439	minus	Repeat_492	31	324915	324945	plus
Repeat_226	42	927343	927384	plus	Repeat_492	31	774729	774759	plus
Repeat_227	42	22776	22817	plus	Repeat_492	31	1052133	1052163	plus
Repeat_227	42	835243	835284	plus	Repeat_493	31	324835	324865	plus
Repeat_228	41	814000	814040	plus	Repeat_493	31	349144	349114	minus
Repeat_228	41	906345	906385	plus	Repeat_493	31	462087	462117	plus
Repeat_229	41	388778	388818	plus	Repeat_493	31	1052088	1052118	plus
Repeat_229	41	424569	424609	plus	Repeat_494	31	324830	324860	plus
Repeat_230	41	153785	153825	plus	Repeat_494	31	371995	371965	minus
Repeat_230	41	325323	325363	plus	Repeat_494	31	462082	462112	plus
Repeat_230	41	702960	703000	plus	Repeat_494	31	773999	773969	minus
Repeat_231	41	324666	324706	plus	Repeat_494	31	909905	909935	plus
Repeat_231	41	1016446	1016406	minus	Repeat_494	31	910061	910091	plus
Repeat_231	41	1016483	1016443	minus	Repeat_494	31	1052083	1052113	plus

Repeat_232	41	322344	322384	plus	Repeat_495	31	322423	322453	plus
Repeat_232	41	1042614	1042574	minus	Repeat_495	31	427063	427093	plus
Repeat_233	41	257725	257765	plus	Repeat_496	31	245848	245818	minus
Repeat_233	41	773618	773578	minus	Repeat_496	31	65984	66014	plus
Repeat_234	41	1185	1225	plus	Repeat_496	31	308893	308923	plus
Repeat_234	41	1107219	1107179	minus	Repeat_496	31	462000	462030	plus
Repeat_235	40	1099393	1099432	plus	Repeat_497	31	307318	307348	plus
Repeat_235	40	1099534	1099573	plus	Repeat_497	31	435637	435607	minus
Repeat_236	40	371544	371583	plus	Repeat_498	31	302209	302239	plus
Repeat_236	40	773583	773622	plus	Repeat_498	31	721140	721110	minus
Repeat_236	40	1035140	1035101	minus	Repeat_498	31	1118164	1118134	minus
Repeat_237	40	619968	620007	plus	Repeat_499	31	279273	279303	plus
Repeat_237	40	773620	773581	minus	Repeat_499	31	926650	926680	plus
Repeat_237	40	852188	852227	plus	Repeat_500	31	278106	278136	plus
Repeat_238	40	777099	777060	minus	Repeat_500	31	476370	476400	plus
Repeat_238	40	591745	591784	plus	Repeat_500	31	640860	640830	minus
Repeat_238	40	1147672	1147711	plus	Repeat_500	31	765903	765873	minus
Repeat_239	40	462174	462213	plus	Repeat_501	31	254545	254575	plus
Repeat_239	40	987308	987269	minus	Repeat_501	31	818178	818148	minus
Repeat_240	40	427117	427156	plus	Repeat_502	31	230775	230805	plus
Repeat_240	40	743118	743079	minus	Repeat_502	31	302306	302336	plus
Repeat_240	40	1035171	1035132	minus	Repeat_502	31	322005	322035	plus
Repeat_241	40	351515	351554	plus	Repeat_503	31	335261	335231	minus
Repeat_241	40	619856	619817	minus	Repeat_503	31	175638	175668	plus
Repeat_241	40	852076	852037	minus	Repeat_504	31	805295	805265	minus
Repeat_242	40	346349	346388	plus	Repeat_504	31	162332	162362	plus
Repeat_242	40	1051319	1051358	plus	Repeat_505	31	151856	151886	plus
Repeat_243	40	324821	324860	plus	Repeat_505	31	231572	231542	minus
Repeat_243	40	909896	909935	plus	Repeat_506	31	116029	116059	plus
Repeat_243	40	1052074	1052113	plus	Repeat_506	31	116166	116196	plus
Repeat_244	40	104938	104977	plus	Repeat_507	31	108648	108678	plus
Repeat_244	40	576170	576209	plus	Repeat_507	31	967254	967284	plus
Repeat_244	40	1132097	1132136	plus	Repeat_507	31	1083102	1083132	plus
Repeat_245	40	65975	66014	plus	Repeat_508	31	108578	108608	plus
Repeat_245	40	245857	245818	minus	Repeat_508	31	967170	967200	plus
Repeat_245	40	461991	462030	plus	Repeat_509	31	90132	90162	plus
Repeat_246	40	51931	51970	plus	Repeat_509	31	90179	90209	plus
Repeat_246	40	922930	922891	minus	Repeat_510	31	80258	80288	plus
Repeat_247	39	883486	883524	plus	Repeat_510	31	750190	750160	minus
Repeat_247	39	922089	922051	minus	Repeat_511	31	78148	78178	plus
Repeat_248	39	773961	773999	plus	Repeat_511	31	78163	78193	plus
Repeat_248	39	909943	909905	minus	Repeat_512	31	244687	244657	minus
Repeat_249	39	593365	593403	plus	Repeat_512	31	67145	67175	plus
Repeat_249	39	593810	593848	plus	Repeat_512	31	971640	971670	plus
Repeat_249	39	1149292	1149330	plus	Repeat_513	31	66037	66067	plus
Repeat_249	39	1149737	1149775	plus	Repeat_513	31	245795	245765	minus
Repeat_250	39	591802	591840	plus	Repeat_513	31	324828	324858	plus
Repeat_250	39	777037	776999	minus	Repeat_513	31	371997	371967	minus
Repeat_250	39	1147729	1147767	plus	Repeat_513	31	761229	761199	minus
Repeat_251	39	590440	590478	plus	Repeat_513	31	909903	909933	plus
Repeat_251	39	932379	932417	plus	Repeat_513	31	910059	910089	plus
Repeat_251	39	1146367	1146405	plus	Repeat_513	31	1052081	1052111	plus
Repeat_252	39	388057	388095	plus	Repeat_514	31	11362	11392	plus
Repeat_252	39	557504	557542	plus	Repeat_514	31	1085630	1085600	minus
Repeat_252	39	1043307	1043345	plus	Repeat_515	30	1099395	1099424	plus
Repeat_252	39	1114243	1114281	plus	Repeat_515	30	1099467	1099496	plus
Repeat_253	39	257664	257626	minus	Repeat_515	30	1099536	1099565	plus
Repeat_253	39	371768	371806	plus	Repeat_516	30	935027	934998	minus
Repeat_253	39	773677	773715	plus	Repeat_516	30	932344	932373	plus
Repeat_254	39	371763	371801	plus	Repeat_517	30	823499	823528	plus
Repeat_254	39	726114	726076	minus	Repeat_517	30	824723	824752	plus

Repeat_254	39	1018167	1018129	minus	Repeat_518	30	726516	726545	plus
Repeat_255	39	371752	371790	plus	Repeat_518	30	1018569	1018598	plus
Repeat_255	39	619904	619866	minus	Repeat_518	30	1106924	1106895	minus
Repeat_255	39	852124	852086	minus	Repeat_519	30	642343	642372	plus
Repeat_256	39	348315	348353	plus	Repeat_519	30	1049832	1049861	plus
Repeat_256	39	1100244	1100282	plus	Repeat_520	30	619966	619995	plus
Repeat_257	39	345529	345567	plus	Repeat_520	30	852186	852215	plus
Repeat_257	39	570454	570492	plus	Repeat_520	30	997858	997887	plus
Repeat_258	39	308606	308644	plus	Repeat_521	30	614314	614343	plus
Repeat_258	39	446141	446103	minus	Repeat_521	30	614336	614365	plus
Repeat_259	39	276214	276252	plus	Repeat_521	30	846534	846563	plus
Repeat_259	39	474478	474516	plus	Repeat_521	30	846556	846585	plus
Repeat_259	39	689673	689711	plus	Repeat_521	30	1170247	1170276	plus
Repeat_260	39	257656	257694	plus	Repeat_521	30	1170269	1170298	plus
Repeat_260	39	325367	325405	plus	Repeat_522	30	593825	593854	plus
Repeat_260	39	427250	427212	minus	Repeat_522	30	594146	594175	plus
Repeat_260	39	462111	462149	plus	Repeat_522	30	1149752	1149781	plus
Repeat_260	39	742985	743023	plus	Repeat_522	30	1150073	1150102	plus
Repeat_260	39	1035038	1035076	plus	Repeat_523	30	560535	560564	plus
Repeat_261	39	228831	228869	plus	Repeat_523	30	943830	943859	plus
Repeat_261	39	419930	419968	plus	Repeat_523	30	953013	953042	plus
Repeat_262	39	147942	147980	plus	Repeat_524	30	471418	471447	plus
Repeat_262	39	310890	310852	minus	Repeat_524	30	471444	471473	plus
Repeat_263	39	104746	104784	plus	Repeat_525	30	458170	458199	plus
Repeat_263	39	575956	575994	plus	Repeat_525	30	592891	592862	minus
Repeat_263	39	1131883	1131921	plus	Repeat_525	30	1148818	1148789	minus
Repeat_264	39	53476	53514	plus	Repeat_526	30	450606	450635	plus
Repeat_264	39	593375	593337	minus	Repeat_526	30	963556	963585	plus
Repeat_264	39	1149302	1149264	minus	Repeat_527	30	371554	371583	plus
Repeat_265	38	750049	750086	plus	Repeat_527	30	427158	427187	plus
Repeat_265	38	786920	786957	plus	Repeat_527	30	462203	462174	minus
Repeat_266	38	420017	420054	plus	Repeat_527	30	773593	773622	plus
Repeat_266	38	1017589	1017626	plus	Repeat_527	30	987279	987308	plus
Repeat_267	38	371544	371581	plus	Repeat_527	30	1035130	1035101	minus
Repeat_267	38	620005	619968	minus	Repeat_528	30	424562	424591	plus
Repeat_267	38	743087	743050	minus	Repeat_528	30	448846	448817	minus
Repeat_267	38	773583	773620	plus	Repeat_529	30	391138	391167	plus
Repeat_267	38	852225	852188	minus	Repeat_529	30	981011	981040	plus
Repeat_267	38	1035140	1035103	minus	Repeat_530	30	388112	388141	plus
Repeat_268	38	619666	619629	minus	Repeat_530	30	797833	797804	minus
Repeat_268	38	851886	851849	minus	Repeat_531	30	371892	371921	plus
Repeat_268	38	351710	351747	plus	Repeat_531	30	462085	462056	minus
Repeat_269	38	324845	324882	plus	Repeat_532	30	619895	619866	minus
Repeat_269	38	513763	513800	plus	Repeat_532	30	852115	852086	minus
Repeat_269	38	1052098	1052135	plus	Repeat_532	30	371761	371790	plus
Repeat_270	38	773618	773581	minus	Repeat_532	30	950623	950652	plus
Repeat_270	38	257725	257762	plus	Repeat_533	30	371753	371782	plus
Repeat_270	38	619970	620007	plus	Repeat_533	30	619903	619874	minus
Repeat_270	38	852190	852227	plus	Repeat_533	30	666535	666506	minus
Repeat_271	38	257350	257387	plus	Repeat_533	30	852123	852094	minus
Repeat_271	38	1117958	1117995	plus	Repeat_533	30	972177	972148	minus
Repeat_272	38	231590	231627	plus	Repeat_534	30	365433	365462	plus
Repeat_272	38	348229	348192	minus	Repeat_534	30	943944	943973	plus
Repeat_273	38	593053	593016	minus	Repeat_535	30	532957	532928	minus
Repeat_273	38	1148980	1148943	minus	Repeat_535	30	357632	357661	plus
Repeat_273	38	54398	54435	plus	Repeat_536	30	1100482	1100453	minus
Repeat_274	38	53350	53387	plus	Repeat_536	30	351138	351167	plus
Repeat_274	38	279071	279108	plus	Repeat_537	30	347125	347154	plus
Repeat_274	38	477335	477372	plus	Repeat_537	30	729817	729846	plus
Repeat_274	38	521926	521963	plus	Repeat_537	30	1021870	1021899	plus
Repeat_275	38	24433	24470	plus	Repeat_538	30	335715	335744	plus

Repeat_275	38	336020	336057	plus	Repeat_538	30	980347	980376	plus
Repeat_276	38	429908	429871	minus	Repeat_539	30	325346	325375	plus
Repeat_276	38	522099	522062	minus	Repeat_539	30	619859	619888	plus
Repeat_276	38	935677	935640	minus	Repeat_539	30	709098	709127	plus
Repeat_276	38	969967	969930	minus	Repeat_539	30	852079	852108	plus
Repeat_276	38	1031	1068	plus	Repeat_540	30	322190	322219	plus
Repeat_277	37	906422	906458	plus	Repeat_540	30	1042354	1042383	plus
Repeat_277	37	909856	909892	plus	Repeat_541	30	309891	309920	plus
Repeat_278	37	771863	771899	plus	Repeat_541	30	1035123	1035094	minus
Repeat_278	37	1042983	1042947	minus	Repeat_542	30	309408	309437	plus
Repeat_279	37	725988	726024	plus	Repeat_542	30	450601	450630	plus
Repeat_279	37	971090	971126	plus	Repeat_542	30	1042941	1042970	plus
Repeat_279	37	1018041	1018077	plus	Repeat_543	30	309034	309063	plus
Repeat_280	37	624057	624093	plus	Repeat_543	30	365391	365420	plus
Repeat_280	37	713467	713503	plus	Repeat_544	30	308967	308996	plus
Repeat_280	37	856277	856313	plus	Repeat_544	30	322307	322336	plus
Repeat_281	37	593932	593968	plus	Repeat_545	30	721003	720974	minus
Repeat_281	37	740007	740043	plus	Repeat_545	30	302345	302374	plus
Repeat_281	37	1032060	1032096	plus	Repeat_546	30	721139	721110	minus
Repeat_281	37	1149859	1149895	plus	Repeat_546	30	1118163	1118134	minus
Repeat_282	37	776895	776859	minus	Repeat_546	30	230821	230850	plus
Repeat_282	37	591939	591975	plus	Repeat_546	30	302210	302239	plus
Repeat_282	37	1147866	1147902	plus	Repeat_546	30	302277	302306	plus
Repeat_283	37	541213	541249	plus	Repeat_547	30	640788	640759	minus
Repeat_283	37	686473	686509	plus	Repeat_547	30	278165	278194	plus
Repeat_284	37	462174	462210	plus	Repeat_547	30	476429	476458	plus
Repeat_284	37	1035101	1035137	plus	Repeat_548	30	1043522	1043493	minus
Repeat_284	37	371583	371547	minus	Repeat_548	30	255539	255568	plus
Repeat_284	37	773622	773586	minus	Repeat_549	30	254577	254606	plus
Repeat_284	37	987308	987272	minus	Repeat_549	30	322423	322452	plus
Repeat_285	37	403499	403535	plus	Repeat_549	30	427063	427092	plus
Repeat_285	37	534943	534979	plus	Repeat_550	30	151886	151857	minus
Repeat_286	37	462210	462174	minus	Repeat_550	30	231542	231571	plus
Repeat_286	37	1035137	1035101	minus	Repeat_550	30	1013740	1013769	plus
Repeat_286	37	371547	371583	plus	Repeat_551	30	720999	720970	minus
Repeat_286	37	773586	773622	plus	Repeat_551	30	230839	230868	plus
Repeat_286	37	987272	987308	plus	Repeat_552	30	721139	721110	minus
Repeat_287	37	349108	349144	plus	Repeat_552	30	1118163	1118134	minus
Repeat_287	37	462123	462087	minus	Repeat_552	30	230821	230850	plus
Repeat_288	37	349084	349120	plus	Repeat_552	30	302210	302239	plus
Repeat_288	37	773649	773685	plus	Repeat_552	30	302277	302306	plus
Repeat_289	37	324959	324995	plus	Repeat_553	30	609072	609043	minus
Repeat_289	37	513808	513844	plus	Repeat_553	30	1165005	1164976	minus
Repeat_290	37	324874	324910	plus	Repeat_553	30	230776	230805	plus
Repeat_290	37	774723	774759	plus	Repeat_553	30	302307	302336	plus
Repeat_290	37	1052127	1052163	plus	Repeat_553	30	322006	322035	plus
Repeat_291	37	257634	257670	plus	Repeat_554	30	168767	168796	plus
Repeat_291	37	560563	560527	minus	Repeat_554	30	578145	578174	plus
Repeat_292	37	257353	257389	plus	Repeat_554	30	1134072	1134101	plus
Repeat_292	37	308608	308644	plus	Repeat_555	30	160013	160042	plus
Repeat_292	37	446139	446103	minus	Repeat_555	30	1070293	1070264	minus
Repeat_293	37	163716	163752	plus	Repeat_556	30	158398	158427	plus
Repeat_293	37	492725	492689	minus	Repeat_556	30	996801	996772	minus
Repeat_294	37	158016	158052	plus	Repeat_557	30	147327	147356	plus
Repeat_294	37	278265	278301	plus	Repeat_557	30	971548	971577	plus
Repeat_294	37	476529	476565	plus	Repeat_558	30	142932	142961	plus
Repeat_295	37	146993	147029	plus	Repeat_558	30	143019	143048	plus
Repeat_295	37	1042625	1042589	minus	Repeat_559	30	113298	113327	plus
Repeat_296	37	66028	66064	plus	Repeat_559	30	963270	963299	plus
Repeat_296	37	245804	245768	minus	Repeat_560	30	113067	113096	plus
Repeat_296	37	773723	773687	minus	Repeat_560	30	997669	997640	minus

Repeat_297	37	53855	53891	plus	Repeat_561	30	106550	106579	plus
Repeat_297	37	522642	522678	plus	Repeat_561	30	1126238	1126267	plus
Repeat_297	37	773611	773647	plus	Repeat_562	30	68937	68966	plus
Repeat_298	37	156924	156888	minus	Repeat_562	30	923188	923217	plus
Repeat_298	37	922500	922464	minus	Repeat_563	30	52828	52857	plus
Repeat_298	37	24604	24640	plus	Repeat_563	30	53958	53987	plus
Repeat_299	36	1014572	1014607	plus	Repeat_563	30	413834	413863	plus
Repeat_299	36	1117777	1117812	plus	Repeat_564	30	335979	335950	minus
Repeat_300	36	460708	460743	plus	Repeat_564	30	51670	51699	plus
Repeat_300	36	617912	617947	plus	Repeat_565	30	427250	427221	minus
Repeat_300	36	707761	707796	plus	Repeat_565	30	37520	37549	plus
Repeat_300	36	850132	850167	plus	Repeat_565	30	257656	257685	plus
Repeat_300	36	1173845	1173880	plus	Repeat_565	30	325367	325396	plus
Repeat_301	36	617393	617428	plus	Repeat_565	30	462111	462140	plus
Repeat_301	36	849613	849648	plus	Repeat_565	30	742985	743014	plus
Repeat_301	36	1015639	1015674	plus	Repeat_565	30	1035038	1035067	plus
Repeat_301	36	1173326	1173361	plus	Repeat_566	30	36190	36219	plus
Repeat_302	36	567326	567361	plus	Repeat_566	30	254758	254787	plus
Repeat_302	36	741448	741413	minus	Repeat_567	30	24582	24611	plus
Repeat_302	36	1033501	1033466	minus	Repeat_567	30	24688	24717	plus
Repeat_303	36	257648	257683	plus	Repeat_567	30	922416	922387	minus
Repeat_303	36	325359	325394	plus	Repeat_567	30	922522	922493	minus
Repeat_303	36	513936	513971	plus	Repeat_568	30	51596	51567	minus
Repeat_303	36	742977	743012	plus	Repeat_568	30	24474	24503	plus
Repeat_303	36	1035030	1035065	plus	Repeat_568	30	336057	336086	plus
Repeat_304	36	815722	815687	minus	Repeat_569	30	21749	21778	plus
Repeat_304	36	448918	448953	plus	Repeat_569	30	1004456	1004485	plus
Repeat_305	36	427488	427523	plus	Repeat_570	30	14953	14982	plus
Repeat_305	36	947678	947643	minus	Repeat_570	30	1001269	1001298	plus

Table S2. plastid-derived DNA segments in *Abies koreana* mitochondrial genome.

Identity	Aligned length	number of mismatches	number of gap openings	Query start	Query end	Hit start	Hit end	E value	Bit-score	Annotation
89.623	106	11	0	85121	85226	1003774	1003879	1.72E-30	135	*petA**
85.437	103	3	4	75680	75778	1075838	1075932	8.13E-19	97.1	*psbK**
93.75	80	5	0	47623	47702	435116	435195	4.82E-26	121	*trnW-CCA*
88.462	78	9	0	48270	48347	350493	350416	2.92E-18	95.3	*ycf1**
94.203	69	4	0	75179	75247	1075344	1075412	1.35E-21	106	*psaA**
92.647	68	1	1	75902	75969	1075994	1076057	2.92E-18	95.3	*psaA**

Table S3. Summary of putative transposable elements (TEs) in *Abies koreana* mitochondrial genome.

Repeat Class	Fragments	Length
**Integrated Virus**	**5**	**304**
Caulimoviridae	5	304
**Interspersed Repeat**	**2**	**264**
**Transposable Element**	**559**	**48,022**
DNA transposon	184	12,380
EnSpm/CACTA	41	2,745
Harbinger	14	1,319
Helitron	27	1,575
Mariner/Tc1	1	79
MuDR	66	4,602
hAT	29	1,601
LTR Retrotransposon	322	29,809
Copia	124	10,764
Gypsy	192	18,188
Non-LTR Retrotransposon	52	5,750
L1	45	5,351
Penelope	6	350
Naiad/Chlamys	6	350
**Multicopy gene**	**6**	**355**
tRNA	6	355
**Simple Repeat**	**4**	**328**
Satellite	3	249
**Total**	**514**	**44,991**

Table S4. Mitochondrial gene clusters among gymnosperms.

No.	gene clusters	*Abies*	*Cycas*	*Ginkgo*	*Welwitschia*	*Pinus*	*Taxus*
1	*atp8-cox3-sdh4*	X	X	X	X	X	O
2	*nad5 e3-nad1 e5*	X	X	X	X	X	X
3	*nad3-rps12*	O	O	O	O	O	O
4	*nad4L-atp4*	X	X	X	X	X	X
5	*rpl16-rps3-rps19-rpl2*	O	O	O	O	O	X
6	*cob-rps14-rpl5*	X	O	O	X	X	X
7	*rps10-cox1*	X	O	O	X	X	X
8	*rps13-nad1 e2-e3*	X	X	X	X	X	X
9	*rrn26- trnfM(CAU)*	X	X	X	X	X	X
10	*rrn18-rrn5*	X	O	O	X	X	O
11	*trnP(TGG-sdh3*	O	O	O	O	O	O
12	*trnP(TGG)cp-trnW(CCA)cp*	X	X	X	X	X	X
13	*trnS(GCT)-trnF(GAA)-trnP(UGG)*	X	X	X	X	X	X
14	*trnY(GTA)-nad2 e5-e4-e3*	X	X	X	X	X	X
	Total	3	6	6	3	3	5

Table S5. Putative chimeric ORFs.

ORFs	ORF start	ORF end	ORF length	Identity	ORF hist start	ORF hit end	Chimera length	Gene hit start	Gene hit end	E-value	Gene	No of transmembrande helices/probabilities
orf226	708727	708047	681	95.5	56	681	626	185	788	0	*atp1*	0/0.028
orf57	707981	707808	174	92	1	174	187	850	1036	2.34E-76	*atp1*	0/07456
orf97	593057<br>1148984	592764<br>1148691	294	96.5	1	56	57	8	64	2.77E-21	*rps11*	0/0.09219
orf70	619024<br>851224	618812<br>851032	213	100	14	100	87	296	382	1.62E-41	*cox2*	0/0.03084
orf69	555940	555731	210	91.4	57	137	81	159	236	5.95E-28	*ccmC*	**1/0.92061**
orf66	255404	255204	201	90.8	1	104	109	1022	1126	2.26E-39	*nad4*	0/0.11072

Table S6. Summary of editing sites detected in the 41 protein-coding genes of the *Abies koreana* mitogenome

Gene	Size	Codon changes by C-to-U editing															PREP-	RNA-seq		Generation of	
	(bp)	A>V	H>Y	L>F	P>F	P>L	P>S	Q>L	Q>*	R>C	R>W	R>*	S>F	S>L	T>I	T>M	Mt	total	Silent	Start	Stop
atp1	1524	1	1	0	0	2	0	0	0	0	0	0	0	2	0	0	6	6	0	No	No
atp4	543	0	0	0	1	7	1	0	1	3	0	0	3	6	0	1	21	24	1	No	Yes
atp6	777	0	3	1	2	14	4	0	1	2	2	0	14	13	1	1	54	61	3	Yes	Yes
atp8	480	0	2	1	0	5	1	0	0	1	1	0	3	1	0	0	21	20	5	No	No
atp9	249	2	1	1	0	0	1	0	0	0	0	1	3	3	0	0	12	13	1	No	Yes
ccmB	624	0	1	0	0	13	3	0	0	4	1	0	6	9	0	0	37	40	3	No	No
ccmC	720	0	4	1	0	9	8	0	0	3	6	0	6	5	1	0	49	51	8	No	No
ccmFc	1395	1	1	2	2	9	3	0	1	6	2	0	5	3	2	0	38	39	2	No	Yes
ccmFn	1728	0	2	3	4	12	5	0	0	7	7	0	5	9	0	0	58	57	3	No	No
cob	1194	0	6	0	6	13	2	0	0	2	8	0	7	6	0	0	51	57	7	No	No
cox1	1596	2	5	5	8	19	7	0	1	2	8	0	18	10	0	1	84	88	2	Yes	Yes
cox2	807	1	1	1	0	3	1	0	0	1	4	0	3	6	0	2	21	23	0	No	No
cox3	798	0	2	2	6	7	3	0	0	1	5	0	4	6	0	0	37	39	3	No	No
matR	2190	0	3	1	0	11	0	0	0	0	2	0	2	1	0	0	30	20	0	No	No
mttB	750	0	2	1	2	10	2	0	0	4	2	0	8	10	1	1	47	47	4	Yes	No
nad1	981	1	2	2	2	11	3	0	0	2	4	0	8	6	0	1	42	47	5	Yes	No
nad2	1473	1	4	1	2	11	7	0	0	5	1	0	11	11	1	2	56	66	9	No	No
nad3	357	0	2	0	0	6	2	0	0	1	1	0	3	4	0	0	25	20	1	No	No
nad4	1578	5	2	4	8	25	10	0	0	3	2	1	16	9	1	2	84	97	9	No	Yes
nad4L	567	0	0	0	0	3	3	0	0	1	1	1	2	8	1	0	19	20	0	No	Yes
nad5	2004	1	6	1	6	19	11	0	0	6	5	0	20	18	1	4	97	108	10	No	No
nad6	615	1	4	2	6	7	2	1	0	1	2	0	6	10	1	1	41	47	3	Yes	No
nad7	1185	2	5	0	0	5	3	0	0	3	0	0	6	14	0	0	38	44	6	No	No
nad9	591	1	0	0	2	4	4	0	0	1	1	0	5	4	0	1	21	26	3	Yes	No
rpl2	1416[A]	0	0	0	0	3	6	0	1	0	1	0	1	5	2	2	25	21	0	Yes	Yes
rpl5	576	0	2	0	0	5	2	0	0	1	0	0	2	3	0	0	17	17	2	No	No
rpl10	483	0	0	0	0	6	0	0	0	0	0	0	1	3	0	0	13	10	0	No	No
rpl16	414	1	0	0	0	3	0	0	1	0	1	0	0	1	3	0	9	11	1	No	Yes
rps1	690	1	0	0	2	7	3	0	0	1	0	0	5	2	3	1	24	27	2	No	No
rps2	720	2	0	0	0	7	1	0	0	2	0	0	5	2	0	0	20	20	1	No	No
rps3	1668	0	4	1	0	4	4	0	0	0	2	0	5	4	0	2	29	33	7	Yes	No
rps4	1053	2	5	0	0	12	4	0	0	1	1	0	5	9	0	1	36	43	3	Yes	No
rps7	588	0	1	0	0	4	1	0	0	0	0	0	0	2	1	1	16	10	0	No	No
rps10	342	0	1	0	2	0	0	0	0	0	1	0	1	3	1	1	11	10	0	Yes	No
rps11	582	1	0	1	0	4	1	0	0	0	0	0	3	1	0	0	3	13	2	No	No
rps12	378	1	0	0	0	3	3	0	0	2	0	0	0	5	1	0	15	15	0	No	No
rps13	489	0	1	0	0	1	0	0	0	1	0	0	0	2	0	0	7	5	0	No	No
rps14	303	0	2	0	0	2	3	0	0	0	1	0	1	2	0	0	11	11	0	No	No
rps19	282	2	0	0	2	0	2	0	0	0	1	0	2	1	0	0	10	12	2	No	No
sdh3	564	0	1	2	2	5	3	0	0	1	0	1	0	3	0	0	24	21	3	No	Yes
sdh4	396	1	1	1	3	4	0	0	0	0	1	0	0	1	0	0	9	17	5	No	No
Total		30	77	34	68	295	119	1	6	68	74	4	195	223	21	25	1268	1356	116	10	10

Table S7. RNA editing in 41 protein-coding genes for *Abies koreana* mitochondrial genome.

gene	Nt Pos	Effect	PREP-Mt Score	RNA-seq	gene	Nt Pos	Effect	PREP-Mt Score	RNA-seq
atp1	398	GCG (A) => GTG (V)	1.00	X	*nad3*	22	CGT (R) => TGT (C)	0.73	O
1524	557	TCG (S) => TTG (L)	1.00	O	357	44	TCG (S) => TTG (L)	1.00	O
	701	GCC (A) => GTC (V)	X	O		62	CCA (P) => CTA (L)	0.95	O
	923	CCA (P) => CTA (L)	1.00	O		80	CCA (P) => CTA (L)	1.00	O
	1345	CAT (H) => TAT (Y)	0.90	O		88	CCC (P) => TCC (S)	0.95	O
	1490	CCG (P) => CTG (L)	0.90	O		124	CAC (H) => TAC (Y)	1.00	O
	1502	TCA (S) => TTA (L)	0.70	O		137	TCC (S) => TTC (F)	1.00	O
atp4	5	CCA (P) => CTA (L)	1.00	O		146	TCC (S) => TTC (F)	1.00	O
543	20	CCA (P) => CTA (L)	0.86	O		185	CCG (P) => CTG (L)	0.95	O
	28	CGT (R) => TGT (C)	1.00	O		190	CCT (P) => TCT (S)	1.00	O
	53	TCA (S) => TTA (L)	1.00	O		197	TCA (S) => TTA (L)	1.00	O
	71	ACG (T) => ATG (M)	1.00	X		209	TCT (S) => TTT (F)	0.95	O
	82	CGT (R) => TGT (C)	0.71	O		215	CCG (P) => CTG (L)	1.00	O
	85	CGT (R) => TGT (C)	1.00	O		230	TCC (S) => TTC (F)	0.86	X
	89	TCC (S) => TTC (F)	1.00	O		231	TCC (S) => TCT (S)	X	O
	90	TCC (S) => TTT (F)	X	O		233	TCC (S) => TTC (F)	1.00	X
	122	TCG (S) => TTG (L)	1.00	O		238	CGG (R) => TGG (W)	1.00	X
	134	TCC (S) => TTC (F)	1.00	O		247	CCT (P) => TCT (S)	1.00	X
	140	GCG (A) => GTG (V)	0.29	X		266	CCG (P) => CTG (L)	1.00	O
	190	CCC (P) => TTC (F)	0.57	X		275	TCT (S) => TTT (F)	1.00	X
	191	CCC (P) => TTC (F)	0.57	O		277	CGG (R) => TGG (W)	1.00	X

atp4	212	TCT (S) => TTT (F)	X	O
	215	CCG (P) => CTG (L)	0.43	O
	284	TCA (S) => TTA (L)	X	O
	290	CCG (P) => CTG (L)	X	O
	299	ACG (T) => ATG (M)	X	O
	344	TCA (S) => TTA (L)	1.00	O
	368	TCA (S) => TTA (L)	1.00	O
	377	CCT (P) => CTT (L)	1.00	O
	380	CCA (P) => CTA (L)	0.71	O
	463	CCC (P) => TCC (S)	0.57	O
	503	CCG (P) => CTG (L)	0.71	O
	527	TCA (S) => TTA (L)	1.00	O
	538	CAA (Q) => TAA (X)	X	O
atp6	2	ACG (T) => ATG (M)	0.25	O
777	5	CCA (P) => CTA (L)	X	O
	25	CCT (P) => TTT (F)	1.00	O
	26	CCT (P) => TTT (F)	1.00	O
	73	CCA (P) => TCA (S)	1.00	O
	77	TCC (S) => TTC (F)	1.00	O
	85	CCA (P) => TCA (S)	0.75	O
	95	TCC (S) => TTC (F)	1.00	O
	101	CCG (P) => CTG (L)	0.92	O
	116	TCA (S) => TTA (L)	0.75	O
	126	CTC (L) => CTT (L)	X	O
	164	TCA (S) => TTA (L)	1.00	O
nad3	296	TCA (S) => TTA (L)	1.00	O
	305	TCG (S) => TTG (L)	1.00	O
	320	CCC (P) => CTC (L)	0.86	O
	322	CAT (H) => TAT (Y)	1.00	O
	349	CGG (R) => TGG (W)	1.00	O
nad4L	20	TCA (S) => TTA (L)	0.38	O
567	28	CCT (P) => TCT (S)	1.00	O
	41	TCT (S) => TTT (F)	1.00	O
	47	TCA (S) => TTA (L)	1.00	O
	55	CGG (R) => TGG (W)	1.00	O
	68	CCA (P) => CTA (L)	1.00	O
	86	ACT (T) => ATT (I)	X	O
	95	TCA (S) => TTA (L)	1.00	O
	100	CCA (P) => TCA (S)	1.00	O
	110	TCA (S) => TTA (L)	1.00	O
	131	TCG (S) => TTG (L)	0.88	O
	140	TCG (S) => TTG (L)	1.00	O
	148	CCC (P) => TCC (S)	1.00	O
	158	CCG (P) => CTG (L)	1.00	O
	191	TCG (S) => TTG (L)	0.62	O
	230	TCG (S) => TTG (L)	1.00	O
	239	CCG (P) => CTG (L)	0.38	O
	281	TCT (S) => TTT (F)	0.88	O
	289	CGC (R) => TGC (C)	0.38	O
	301	CGA (R) => TGA (X)	0.88	O

atp6		178	CGG (R) => TGG (W)	1.00	O	*nad4*		16	CGT (R) => TGT (C)	0.75	X
		188	TCG (S) => TTG (L)	0.83	X		1578	29	TCC (S) => TTC (F)	0.67	O
		202	CAT (H) => TAT (Y)	1.00	O			30	TCC (S) => TTT (F)	X	O
		215	CCG (P) => CTG (L)	1.00	O			44	CCT (P) => CTT (L)	1.00	O
		249	TCC (S) => TCT (S)	X	O			77	CCT (P) => CTT (L)	0.78	O
		269	TCT (S) => TTT (F)	1.00	O			83	TCC (S) => TTC (F)	0.67	O
		272	TCC (S) => TTC (F)	1.00	O			117	TTC (F) => TTT (F)	X	O
		277	CGC (R) => TGC (C)	0.75	O			137	CCT (P) => CTT (L)	1.00	O
		284	TCG (S) => TTG (L)	0.67	O			149	TCG (S) => TTG (L)	0.89	O
		292	CTT (L) => TTT (F)	1.00	O			154	CCC (P) => TCC (S)	1.00	O
		299	TCT (S) => TTT (F)	1.00	O			156	CCC (P) => TCT (S)	X	O
		302	TCG (S) => TTG (L)	1.00	O			158	CCT (P) => CTT (L)	1.00	O
		305	TCA (S) => TTA (L)	1.00	O			164	CCC (P) => CTC (L)	0.67	O
		310	CGT (R) => TGT (C)	1.00	O			166	CGG (R) => TGG (W)	1.00	O
		317	CCC (P) => CTC (L)	1.00	O			184	CCT (P) => TCT (S)	0.89	O
		341	TCC (S) => TTC (F)	1.00	O			197	TCC (S) => TTC (F)	1.00	O
		359	TCT (S) => TTT (F)	1.00	O			249	TAC (Y) => TAT (Y)	X	O
		371	TCG (S) => TTG (L)	0.92	O			251	ACG (T) => ATG (M)	0.22	O
		379	CCA (P) => TCA (S)	1.00	O			268	CCC (P) => TCC (S)	1.00	O
		392	TCT (S) => TTT (F)	1.00	O			271	CCA (P) => TTA (L)	X	O
		416	TCT (S) => TTT (F)	1.00	O			272	CCA (P) => TTA (L)	1.00	O
		440	TCC (S) => TTC (F)	0.58	O			278	TCT (S) => TTT (F)	1.00	O
		449	TCC (S) => TTC (F)	X	O			317	TCA (S) => TTA (L)	1.00	O
		450	TCC (S) => TTT (F)	X	O			335	ACG (T) => ATG (M)	0.67	X
		452	TCA (S) => TTA (L)	1.00	O			365	GCA (A) => GTA (V)	X	O

atp6	488	TCA (S) => TTA (L)	1.00	O	*nad4*	371	CCA (P) => CTA (L)	0.89	O
	508	CCT (P) => TCT (S)	1.00	O		376	CGC (R) => TGC (C)	0.78	O
	511	CAT (H) => TAT (Y)	1.00	O		393	ATC (I) => ATT (I)	X	O
	527	TCA (S) => TTA (L)	1.00	O		401	TCT (S) => TTT (F)	1.00	O
	548	TCC (S) => TTC (F)	1.00	O		403	CGC (R) => TGC (C)	1.00	O
	575	TCA (S) => TTA (L)	1.00	O		410	CCG (P) => CTG (L)	1.00	O
	586	CCA (P) => TTA (L)	X	O		419	TCA (S) => TTA (L)	1.00	O
	587	CCA (P) => TTA (L)	1.00	O		427	CAT (H) => TAT (Y)	0.89	O
	601	CGG (R) => TGG (W)	1.00	O		433	CTT (L) => TTT (F)	1.00	O
	611	CCA (P) => CTA (L)	1.00	O		436	CCC (P) => TTC (F)	0.89	O
	635	TCC (S) => TTC (F)	0.83	O		437	CCC (P) => TTC (F)	0.89	O
	647	CCG (P) => CTG (L)	0.92	O		467	ACT (T) => ATT (I)	1.00	X
	656	CCA (P) => CTA (L)	0.75	O		520	CCC (P) => TTC (F)	1.00	O
	658	CCA (P) => TTA (L)	X	O		521	CCC (P) => TTC (F)	1.00	O
	659	CCA (P) => TTA (L)	X	O		524	CCA (P) => CTA (L)	1.00	O
	674	CCG (P) => CTG (L)	1.00	O		533	TCA (S) => TTA (L)	1.00	O
	683	TCG (S) => TTG (L)	1.00	O		537	CTC (L) => CTT (L)	X	O
	704	TCA (S) => TTA (L)	1.00	O		547	CTT (L) => TTT (F)	1.00	O
	712	CAT (H) => TAT (Y)	1.00	O		566	CCG (P) => CTG (L)	1.00	O
	719	TCT (S) => TTT (F)	1.00	O		574	CTC (L) => TTC (F)	X	O
	728	TCA (S) => TTA (L)	1.00	O		578	TCC (S) => TTC (F)	0.67	O
	743	CCG (P) => CTG (L)	1.00	O		611	TCA (S) => TTA (L)	0.89	O
	755	ACA (T) => ATA (I)	0.92	O		623	TCC (S) => TTC (F)	0.89	O
	761	CCT (P) => CTT (L)	1.00	O		647	CCA (P) => CTA (L)	1.00	O
	766	CAA (Q) => TAA (X)	1.00	O		659	TCT (S) => TTT (F)	1.00	O

atp8	11	CCG (P) => CTG (L)	1.00	O	*nad4*	661	CCC (P) => TTC (F)	1.00	O
480	20	TCC (S) => TTC (F)	0.75	O		662	CCC (P) => TTC (F)	1.00	O
	29	TCC (S) => TTC (F)	1.00	O		667	CCT (P) => TCT (S)	0.89	O
	30	TCC (S) => TTC (F)	X	O		673	CCC (P) => TCC (S)	X	O
	43	CGG (R) => TGG (W)	1.00	O		755	TCG (S) => TTG (L)	1.00	O
	61	CTC (L) => TTC (F)	0.88	O		770	TCA (S) => TTA (L)	1.00	O
	63	CTC (L) => TTT (F)	X	O		794	TCG (S) => TTG (L)	1.00	O
	68	TCC (S) => TTC (F)	1.00	O		802	CCC (P) => TCC (S)	1.00	O
	69	TCC (S) => TTT (F)	X	O		836	TCC (S) => TTC (F)	1.00	O
	70	CAT (H) => TAT (Y)	1.00	O		863	GCG (A) => GTG (V)	X	O
	77	TCC (S) => TTC (F)	0.38	X		887	CCG (P) => CTG (L)	1.00	O
	80	CCA (P) => CTA (L)	0.25	O		934	CCA (P) => TCA (S)	1.00	O
	98	CCT (P) => CTT (L)	1.00	X		947	ACG (T) => ATG (M)	1.00	O
	116	CCC (P) => CTC (L)	1.00	O		953	TCT (S) => TTT (F)	0.44	O
	122	CCA (P) => CTA (L)	1.00	O		971	TCC (S) => TTC (F)	1.00	O
	139	CCA (P) => TCA (S)	1.00	X		977	CCG (P) => CTG (L)	0.78	O
	194	TCG (S) => TTG (L)	1.00	O		1007	CCA (P) => CTA (L)	1.00	O
	223	CAT (H) => TAT (Y)	1.00	O		1010	CCG (P) => CTG (L)	1.00	O
	232	CCC (P) => TCC (S)	0.75	O		1028	CCG (P) => CTG (L)	1.00	O
	234	CCC (P) => TCT (S)	X	O		1033	CCT (P) => TCT (S)	1.00	O
	269	GCC (A) => GTC (V)	1.00	X		1036	CCA (P) => TCA (S)	1.00	O
	310	CGT (R) => TGT (C)	0.75	O		1043	CCT (P) => CTT (L)	1.00	O
	354	ATC (I) => ATT (I)	X	O		1046	TCT (S) => TTT (F)	1.00	O
	356	CCA (P) => CTA (L)	0.50	O		1049	CCA (P) => CTA (L)	1.00	O
	367	CCG (P) => TCG (S)	1.00	X		1088	CCT (P) => CTT (L)	0.89	O

atp8		391	CCC (P) => TCC (S)	0.75	X
atp9		65	GCC (A) => GTC (V)	0.47	O
	249	80	GCT (A) => GTT (V)	1.00	O
		82	CTT (L) => TTT (F)	1.00	O
		92	TCG (S) => TTG (L)	1.00	O
		96	ATC (I) => ATT (I)	X	O
		100	CCC (P) => TCC (S)	0.95	O
		137	TCT (S) => TTT (F)	1.00	O
		142	CAC (H) => TAC (Y)	1.00	O
		185	TCT (S) => TTT (F)	1.00	O
		191	TCA (S) => TTA (L)	1.00	O
		212	TCA (S) => TTA (L)	1.00	O
		215	TCC (S) => TTC (F)	1.00	O
		223	CGA (R) => TGA (X)	1.00	O
ccmB		28	CAT (H) => TAT (Y)	0.89	O
	624	73	CCA (P) => TCA (S)	X	O
		74	CCA (P) => CTA (L)	1.00	X
		116	TCT (S) => TTT (F)	1.00	O
		131	TCA (S) => TTA (L)	1.00	O
		140	TCC (S) => TTC (F)	1.00	O
		151	CCG (P) => TTG (L)	X	O
		152	CCG (P) => TTG (L)	1.00	O
		157	CGG (R) => TGG (W)	1.00	O
		163	CCT (P) => TCT (S)	0.67	O
		175	CCT (P) => TCT (S)	0.89	O

nad4	1091	GCT (A) => GTT (V)	1.00	O
	1109	TCA (S) => TTA (L)	1.00	O
	1129	CCC (P) => TTC (F)	1.00	O
	1130	CCC (P) => TTC (F)	1.00	O
	1132	CCT (P) => TCT (S)	1.00	O
	1136	ACC (T) => ATC (I)	X	O
	1148	TCT (S) => TTT (F)	0.89	O
	1151	TCC (S) => TTC (F)	1.00	O
	1152	TCC (S) => TTT (F)	X	O
	1190	TCT (S) => TTT (F)	1.00	O
	1205	CCC (P) => CTC (L)	1.00	O
	1230	ATC (I) => ATT (I)	X	O
	1289	CCT (P) => CTT (L)	0.78	O
	1307	GCG (A) => GTG (V)	1.00	O
	1312	CTT (L) => TTT (F)	0.33	O
	1355	CCA (P) => CTA (L)	1.00	O
	1373	CCG (P) => CTG (L)	X	O
	1403	GCT (A) => GTT (V)	0.78	O
	1405	CGG (R) => TGG (W)	1.00	O
	1417	CAC (H) => TAC (Y)	1.00	O
	1430	TCC (S) => TTC (F)	1.00	O
	1431	TCC (S) => TTT (F)	X	O
	1433	CCG (P) => CTG (L)	1.00	O
	1438	CGT (R) => TGT (C)	1.00	O
	1486	CGA (R) => TGA (X)	0.88	O

ccmB	178	CTT (L) => TTT (F)	1.00	X
	184	CCC (P) => TCC (S)	0.78	X
	186	CCC (P) => CCT (P)	X	O
	197	TCT (S) => TTT (F)	0.89	O
	224	CCC (P) => CTC (L)	1.00	O
	225	CCC (P) => CTT (L)	X	O
	230	TCG (S) => TTG (L)	1.00	O
	239	TCA (S) => TTA (L)	1.00	O
	307	CGT (R) => TGT (C)	0.78	O
	316	CGT (R) => TGT (C)	0.89	O
	341	CCG (P) => CTG (L)	1.00	O
	383	CCA (P) => CTA (L)	0.78	O
	386	CCA (P) => CTA (L)	0.78	O
	394	CCG (P) => TTG (L)	X	O
	395	CCG (P) => TTG (L)	0.89	O
	427	CGT (R) => TGT (C)	0.89	O
	431	TCG (S) => TTG (L)	1.00	O
	478	CCA (P) => TTA (L)	X	O
	479	CCA (P) => TTA (L)	0.89	O
	500	TCG (S) => TTG (L)	1.00	O
	506	CCA (P) => CTA (L)	1.00	O
	517	CGT (R) => TGT (C)	1.00	O
	528	ATC (I) => ATT (I)	X	O
	551	CCT (P) => CTT (L)	0.78	O
	554	TCA (S) => TTA (L)	1.00	O
nad5	20	TCG (S) => TTG (L)	X	O
2004	32	CCC (P) => CTC (L)	0.90	O
	33	CCC (P) => CTT (L)	X	O
	119	TCT (S) => TTT (F)	X	O
	134	ACT (T) => ATT (I)	0.90	X
	140	TCT (S) => TTT (F)	1.00	O
	142	CAT (H) => TAT (Y)	1.00	O
	155	CCG (P) => CTG (L)	1.00	O
	176	CCA (P) => CTA (L)	0.70	O
	190	CGG (R) => TGG (W)	1.00	O
	220	CGG (R) => TGG (W)	1.00	O
	227	TCC (S) => TTC (F)	1.00	O
	242	CCG (P) => CTG (L)	1.00	O
	246	ACC (T) => ACT (T)	X	O
	257	CCA (P) => CTA (L)	1.00	O
	293	CCT (P) => CTT (L)	0.70	O
	295	CAT (H) => TAT (Y)	1.00	O
	298	CCC (P) => TCC (S)	1.00	O
	346	CAT (H) => TAT (Y)	1.00	O
	350	TCA (S) => TTA (L)	1.00	O
	358	CCT (P) => TTT (F)	1.00	O
	359	CCT (P) => TTT (F)	1.00	O
	371	ACG (T) => ATG (M)	1.00	O
	374	CCA (P) => CTA (L)	0.90	O
	380	TCG (S) => TTG (L)	1.00	O

ccmB	557	TCG (S) => TTG (L)	0.89	O
	569	TCC (S) => TTC (F)	0.78	O
	572	TCT (S) => TTT (F)	0.78	O
	575	CCG (P) => CTG (L)	1.00	O
	581	GCA (A) => GTA (V)	0.78	X
	584	TCT (S) => TTT (F)	X	O
	599	TCG (S) => TTG (L)	0.89	O
	614	TCA (S) => TTA (L)	0.89	O
	623	TCA (S) => TTA (L)	0.22	X
ccmC	6	TTC (F) => TTT (F)	X	O
720	10	CCG (P) => TCG (S)	0.62	O
	63	ATC (I) => ATT (I)	X	O
	75	TTC (F) => TTT (F)	X	O
	76	CGG (R) => TGG (W)	0.78	O
	103	CAT (H) => TAT (Y)	1.00	O
	115	CGG (R) => TGG (W)	0.78	O
	128	TCA (S) => TTA (L)	0.56	X
	134	TCT (S) => TTT (F)	0.67	O
	151	CAT (H) => TAT (Y)	0.22	O
	159	ATC (I) => ATT (I)	X	O
	161	ACC (T) => ATC (I)	0.22	O
	163	CAT (H) => TAT (Y)	1.00	O
	179	GCA (A) => GTA (V)	0.78	X
	184	CGG (R) => TGG (W)	1.00	O
	197	CCT (P) => CTT (L)	0.56	O
nad5	387	ACC (T) => ACT (T)	X	O
	413	CCG (P) => CTG (L)	1.00	O
	439	CCA (P) => TCA (S)	1.00	O
	446	TCG (S) => TTG (L)	1.00	O
	449	TCA (S) => TTA (L)	1.00	O
	458	TCC (S) => TTC (F)	1.00	O
	459	TCC (S) => TTT (F)	X	O
	460	CGG (R) => TGG (W)	1.00	O
	464	TCC (S) => TTC (F)	1.00	O
	533	TCA (S) => TTA (L)	1.00	O
	557	TCT (S) => TTT (F)	1.00	O
	581	TCC (S) => TTC (F)	1.00	O
	583	CCG (P) => TCG (S)	0.90	O
	593	TCT (S) => TTT (F)	1.00	O
	598	CGT (R) => TGT (C)	1.00	O
	640	CGC (R) => TGC (C)	0.90	O
	673	CGT (R) => TGT (C)	1.00	O
	678	ATC (I) => ATT (I)	X	O
	680	TCA (S) => TTA (L)	1.00	O
	683	CCC (P) => CTC (L)	0.90	O
	684	CCC (P) => CTT (L)	X	O
	686	TCT (S) => TTT (F)	0.90	O
	722	TCG (S) => TTG (L)	1.00	O
	730	CGG (R) => TGG (W)	1.00	O
	734	TCA (S) => TTA (L)	1.00	O

ccmC	202	CAT (H) => TAT (Y)	1.00	O	*nad5*	746	ACG (T) => ATG (M)	1.00	O
	232	CTC (L) => TTC (F)	1.00	O		773	CCG (P) => CTG (L)	1.00	O
	236	TCA (S) => TTA (L)	1.00	O		791	ACG (T) => ATG (M)	1.00	O
	260	CCC (P) => CTC (L)	0.78	X		808	CCC (P) => TTC (F)	1.00	O
	268	CCC (P) => TCC (S)	1.00	O		809	CCC (P) => TTC (F)	1.00	O
	281	ACA (T) => ATA (I)	0.56	X		833	CCA (P) => CTA (L)	1.00	O
	299	TCC (S) => TTC (F)	1.00	O		836	TCT (S) => TTT (F)	1.00	O
	300	TCC (S) => TTT (F)	X	O		844	CCA (P) => TCA (S)	0.90	O
	314	TCA (S) => TTA (L)	1.00	O		857	TCG (S) => TTG (L)	1.00	O
	331	CGG (R) => TGG (W)	1.00	O		896	TCC (S) => TTC (F)	0.30	O
	358	CGG (R) => TGG (W)	1.00	O		929	TCA (S) => TTA (L)	1.00	O
	381	ACC (T) => ACT (T)	X	O		946	CAT (H) => TAT (Y)	1.00	O
	382	CCT (P) => TCT (S)	1.00	O		949	CCA (P) => TCA (S)	1.00	O
	395	TCG (S) => TTG (L)	1.00	O		955	CGC (R) => TGC (C)	1.00	O
	398	TCT (S) => TTT (F)	0.78	O		979	CTT (L) => TTT (F)	1.00	O
	400	CTT (L) => TTT (F)	0.89	X		985	CGC (R) => TGC (C)	1.00	O
	410	CCG (P) => CTG (L)	1.00	O		996	TCC (S) => TCT (S)	X	O
	419	CCG (P) => CTG (L)	1.00	O		1016	TCT (S) => TTT (F)	1.00	O
	421	CGT (R) => TGT (C)	0.78	O		1022	TCA (S) => TTA (L)	1.00	O
	425	TCC (S) => TTC (F)	1.00	O		1069	CCA (P) => TCA (S)	1.00	O
	436	CCT (P) => TCT (S)	0.89	O		1103	ACG (T) => ATG (M)	1.00	O
	446	CCG (P) => CTG (L)	0.78	O		1157	CCC (P) => CTC (L)	1.00	O
	451	CCT (P) => TCT (S)	1.00	O		1193	CCA (P) => CTA (L)	1.00	O
	458	TCA (S) => TTA (L)	0.78	O		1207	CCC (P) => TCC (S)	1.00	O
	463	CGT (R) => TGT (C)	1.00	O		1223	TCA (S) => TTA (L)	1.00	O

ccmC	473	CCG (P) => CTG (L)	1.00	O	*nad5*	1263	TTC (F) => TTT (F)	X	O
	497	TCC (S) => TTC (F)	1.00	O		1292	TCT (S) => TTT (F)	0.20	O
	499	CCC (P) => TCC (S)	1.00	O		1300	CCT (P) => TCT (S)	1.00	O
	501	CCC (P) => TCT (S)	X	O		1306	CAC (H) => TAC (Y)	1.00	O
	508	CGG (R) => TGG (W)	1.00	O		1319	TCG (S) => TTG (L)	1.00	O
	521	TCG (S) => TTG (L)	1.00	O		1328	CCG (P) => CTG (L)	1.00	O
	548	TCT (S) => TTT (F)	1.00	O		1337	CCA (P) => CTA (L)	1.00	O
	568	CCT (P) => TCT (S)	1.00	O		1340	GCA (A) => GTA (V)	0.80	O
	612	TTC (F) => TTT (F)	X	O		1351	CCA (P) => TCA (S)	0.90	O
	614	CCA (P) => CTA (L)	0.78	O		1409	TCA (S) => TTA (L)	1.00	O
	619	CGT (R) => TGT (C)	0.78	O		1433	CCT (P) => CTT (L)	0.80	O
	628	CTC (L) => TTC (F)	0.89	X		1467	ATC (I) => ATT (I)	X	O
	650	CCT (P) => CTT (L)	0.78	O		1484	TCT (S) => TTT (F)	1.00	O
	656	CCA (P) => CTA (L)	0.89	O		1486	CGG (R) => TGG (W)	1.00	O
	665	CCC (P) => CTC (L)	0.78	O		1495	CCC (P) => TCC (S)	1.00	O
	673	CCC (P) => TCC (S)	0.78	O		1499	CCT (P) => CTT (L)	0.80	O
ccmFc	11	CCG (P) => CTG (L)	1.00	O		1502	TCC (S) => TTC (F)	0.80	O
1395	35	ACT (T) => ATT (I)	X	O		1559	ACC (T) => ATC (I)	0.90	O
	38	TCC (S) => TTC (F)	0.83	O		1565	CCA (P) => CTA (L)	0.90	O
	50	CCT (P) => CTT (L)	1.00	O		1580	TCT (S) => TTT (F)	1.00	O
	52	CGT (R) => TGT (C)	1.00	O		1661	TCT (S) => TTT (F)	0.80	O
	87	TTC (F) => TTT (F)	X	O		1675	CAT (H) => TAT (Y)	1.00	O
	103	CCC (P) => TCC (S)	1.00	O		1682	TCT (S) => TTT (F)	1.00	O
	107	ACA (T) => ATA (I)	X	O		1702	CCC (P) => TTC (F)	1.00	O
	119	TCT (S) => TTT (F)	1.00	O		1703	CCC (P) => TTC (F)	1.00	O

ccmFc	122	TCC (S) => TTC (F)	1.00	O
	145	CCT (P) => TTT (F)	X	O
	146	CCT (P) => TTT (F)	1.00	O
	149	ACA (T) => ATA (I)	1.00	X
	160	CCC (P) => TCC (S)	0.67	X
	164	TCT (S) => TTT (F)	0.33	X
	181	CCC (P) => TCC (S)	0.83	O
	245	CCT (P) => CTT (L)	X	O
	280	CCC (P) => TCC (S)	1.00	X
	305	TCA (S) => TTA (L)	0.83	O
	314	TCC (S) => TTC (F)	1.00	O
	319	CTC (L) => TTC (F)	X	O
	368	CCA (P) => CTA (L)	0.83	O
	379	CGC (R) => TGC (C)	1.00	O
	381	CGC (R) => TGT (C)	X	O
	391	CGT (R) => TGT (C)	1.00	O
	406	CGT (R) => TGT (C)	0.83	O
	412	CTT (L) => TTT (F)	0.67	X
	415	CTC (L) => TTC (F)	1.00	X
	422	CCA (P) => CTA (L)	1.00	X
	545	CCC (P) => CTC (L)	0.67	X
	551	CCA (P) => CTA (L)	1.00	X
	731	GCT (A) => GTT (V)	0.83	X
	886	CAT (H) => TAT (Y)	X	O
	890	TCA (S) => TTA (L)	1.00	O

nad5	1730	GCC (A) => GTC (V)	0.70	X
	1775	TCA (S) => TTA (L)	1.00	O
	1869	TTC (F) => TTT (F)	X	O
	1925	TCT (S) => TTT (F)	0.70	O
	1927	CGT (R) => TGT (C)	0.70	O
	1948	CCT (P) => TCT (S)	0.70	O
	1967	TCG (S) => TTG (L)	0.90	O
	1979	TCG (S) => TTG (L)	0.50	O
	1997	TCC (S) => TTC (F)	0.20	O
	2000	CCA (P) => CTA (L)	X	O
nad6	2	ACG (T) => ATG (M)	X	O
615	8	TCT (S) => TTT (F)	1.00	O
	17	TCG (S) => TTG (L)	0.70	O
	19	CCG (P) => TCG (S)	0.90	O
	26	CCT (P) => CTT (L)	0.40	O
	36	GTC (V) => GTT (V)	X	O
	76	CCC (P) => TCC (S)	1.00	O
	80	GCT (A) => GTT (V)	0.90	O
	83	TCG (S) => TTG (L)	1.00	O
	87	TTC (F) => TTT (F)	X	O
	88	CCC (P) => TTC (F)	0.70	O
	89	CCC (P) => TTC (F)	0.70	O
	95	CCA (P) => CTA (L)	1.00	O
	103	CGC (R) => TGC (C)	1.00	O
	142	CTC (L) => TTC (F)	1.00	O

ccmFc	1049	TCT (S) => TTT (F)	1.00	O	*nad6*	145	CCT (P) => TTT (F)	1.00	O
	1057	CGT (R) => TGT (C)	X	O		146	CCT (P) => TTT (F)	1.00	O
	1169	GCT (A) => GTT (V)	1.00	O		158	TCC (S) => TTC (F)	1.00	O
	1198	CCA (P) => TTA (L)	X	O		161	CCA (P) => CTA (L)	1.00	O
	1199	CCA (P) => TTA (L)	1.00	O		169	CAT (H) => TAT (Y)	1.00	O
	1208	CCG (P) => CTG (L)	1.00	O		191	TCA (S) => TTA (L)	1.00	O
	1262	CCG (P) => CTG (L)	1.00	O		194	TCC (S) => TTC (F)	1.00	O
	1267	CTT (L) => TTT (F)	1.00	O		195	TCC (S) => TTT (F)	X	O
	1276	CGC (R) => TGC (C)	X	O		199	CCC (P) => TTC (F)	1.00	O
	1282	CGG (R) => TGG (W)	1.00	O		200	CCC (P) => TTC (F)	1.00	O
	1303	CCG (P) => TCG (S)	1.00	O		251	TCG (S) => TTG (L)	0.90	O
	1307	CCG (P) => CTG (L)	1.00	O		260	CCA (P) => CTA (L)	1.00	O
	1352	TCG (S) => TTG (L)	1.00	O		289	CTT (L) => TTT (F)	1.00	O
	1357	CGG (R) => TGG (W)	1.00	O		292	CGG (R) => TGG (W)	0.70	O
	1363	CAA (Q) => TAA (X)	1.00	O		314	CCA (P) => CTA (L)	0.70	O
ccmFn	38	CCG (P) => CTG (L)	1.00	O		325	CAC (H) => TAC (Y)	0.22	O
1728	47	TCC (S) => TTC (F)	1.00	O		335	TCA (S) => TTA (L)	0.78	O
	61	CAC (H) => TAC (Y)	1.00	O		379	CAT (H) => TAT (Y)	0.90	O
	86	TCT (S) => TTT (F)	1.00	O		410	TCG (S) => TTG (L)	1.00	O
	98	CCT (P) => CTT (L)	1.00	O		419	TCG (S) => TTG (L)	1.00	O
	113	ACT (T) => ATT (I)	1.00	X		428	TCA (S) => TTA (L)	1.00	O
	124	CTC (L) => TTC (F)	1.00	O		433	CAT (H) => TAT (Y)	1.00	O
	134	CCT (P) => CTT (L)	1.00	O		446	TCC (S) => TTC (F)	1.00	O
	137	TCG (S) => TTG (L)	1.00	O		451	CGG (R) => TGG (W)	0.70	O
	142	CGT (R) => TGT (C)	1.00	O		458	TCG (S) => TTG (L)	1.00	O

ccmFn	149	ACT (T) => ATT (I)	1.00	X
	151	CCT (P) => TCT (S)	0.83	O
	163	CCC (P) => TCC (S)	1.00	O
	208	CTT (L) => TTT (F)	1.00	X
	254	CCA (P) => CTA (L)	1.00	O
	257	CCA (P) => CTA (L)	1.00	O
	259	CGG (R) => TGG (W)	1.00	O
	265	CGG (R) => TGG (W)	1.00	O
	272	CCA (P) => CTA (L)	0.83	O
	290	CCC (P) => CTC (L)	0.67	O
	295	CGT (R) => TGT (C)	1.00	O
	365	TCC (S) => TTC (F)	0.67	O
	372	GTC (V) => GTT (V)	X	O
	374	TCG (S) => TTG (L)	0.67	O
	381	TTC (F) => TTT (F)	X	O
	403	CTC (L) => TTC (F)	X	O
	408	CCC (P) => CCT (P)	X	O
	455	CCA (P) => CTA (L)	0.67	X
	514	CTT (L) => TTT (F)	X	O
	584	TCT (S) => TTT (F)	0.50	X
	623	TCG (S) => TTG (L)	X	O
	697	CCC (P) => TCC (S)	1.00	X
	748	CTT (L) => TTT (F)	0.67	X
	752	TCC (S) => TTC (F)	1.00	O
	758	TCG (S) => TTG (L)	0.83	O
nad6	470	CCT (P) => CTT (L)	1.00	O
	476	CCA (P) => CTA (L)	1.00	O
	479	TCA (S) => TTA (L)	1.00	O
	500	ACA (T) => ATA (I)	1.00	O
	569	TCC (S) => TTC (F)	1.00	O
	570	TCC (S) => TTT (F)	X	O
	602	CAA (Q) => CTA (L)	X	O
nad7	38	TCG (S) => TTG (L)	0.75	O
1185	45	TTC (F) => TTT (F)	X	O
	74	GCT (A) => GTT (V)	1.00	O
	77	TCA (S) => TTA (L)	1.00	O
	83	TCA (S) => TTA (L)	1.00	O
	137	TCA (S) => TTA (L)	1.00	O
	160	CTA (L) => TTA (L)	X	O
	209	TCA (S) => TTA (L)	0.88	O
	247	CCT (P) => TCT (S)	1.00	O
	251	TCA (S) => TTA (L)	1.00	O
	268	CTG (L) =>TTG (L)	X	O
	274	CGC (R) => TGC (C)	1.00	O
	316	CGT (R) => TGT (C)	1.00	O
	335	TCG (S) => TTG (L)	0.88	O
	344	TCA (S) => TTA (L)	1.00	O
	392	TCC (S) => TTC (F)	1.00	O
	404	TCT (S) => TTT (F)	1.00	O
	422	TCG (S) => TTG (L)	1.00	O

ccmFn	796	CGT (R) => TGT (C)	1.00	O	*nad7*	433	CAT (H) => TAT (Y)	1.00	O
	805	CCG (P) => TCG (S)	X	O		445	CCG (P) => TCG (S)	0.88	O
	818	TCA (S) => TTA (L)	1.00	O		566	TCA (S) => TTA (L)	0.88	O
	830	CCG (P) => CTG (L)	1.00	O		578	TCA (S) => TTA (L)	0.88	O
	862	CGC (R) => TGC (C)	1.00	O		656	TCC (S) => TTC (F)	1.00	O
	979	CGC (R) => TGC (C)	1.00	O		657	TCC (S) => TTT (F)	X	O
	1108	CTC (L) => TTC (F)	1.00	X		671	CCA (P) => CTA (L)	1.00	O
	1114	CTC (L) => TTC (F)	1.00	X		698	TCG (S) => TTG (L)	1.00	O
	1208	TCG (S) => TTG (L)	X	O		722	GCC (A) => GTC (V)	1.00	O
	1286	CCG (P) => CTG (L)	1.00	O		724	CAT (H) => TAT (Y)	1.00	O
	1294	CGG (R) => TGG (W)	1.00	O		734	TCG (S) => TTG (L)	0.88	O
	1322	CCA (P) => CTA (L)	1.00	O		740	TCT (S) => TTT (F)	1.00	O
	1339	CAT (H) => TAT (Y)	1.00	O		781	CAC (H) => TAC (Y)	1.00	O
	1354	CGG (R) => TGG (W)	1.00	O		815	CCT (P) => CTT (L)	0.50	O
	1363	CGG (R) => TGG (W)	1.00	O		836	CCT (P) => CTT (L)	1.00	O
	1372	CGG (R) => TGG (W)	1.00	O		913	CCC (P) => TCC (S)	1.00	O
	1396	CCT (P) => TTT (F)	1.00	O		926	TCA (S) => TTA (L)	0.88	O
	1397	CCT (P) => TTT (F)	1.00	O		944	CCC (P) => CTC (L)	1.00	O
	1405	CGG (R) => TGG (W)	1.00	O		946	CAT (H) => TAT (Y)	1.00	O
	1423	CGT (R) => TGT (C)	1.00	O		963	TCC (S) => TCT (S)	X	O
	1432	CCA (P) => TCA (S)	1.00	O		982	CAT (H) => TAT (Y)	1.00	O
	1472	CCC (P) => CTC (L)	0.83	O		1057	CGT (R) => TGT (C)	1.00	O
	1490	TCA (S) => TTA (L)	1.00	O		1079	TCT (S) => TTT (F)	1.00	O
	1492	CGC (R) => TGC (C)	1.00	O		1083	GCC (A) => GCT (A)	X	O
	1502	TCA (S) => TTA (L)	1.00	O		1103	TCT (S) => TTT (F)	1.00	O

ccmFn	1511	TCT (S) => TTT (F)	1.00	O
	1514	TCG (S) => TTG (L)	X	O
	1532	CCA (P) => CTA (L)	1.00	O
	1537	CCC (P) => TCC (S)	1.00	O
	1549	CCT (P) => TTT (F)	1.00	O
	1550	CCT (P) => TTT (F)	1.00	O
	1585	CGG (R) => TGG (W)	0.67	X
cob	24	TTC (F) => TTT (F)	X	O
1194	25	CCT (P) => TCT (S)	1.00	O
	32	CCC (P) => CTC (L)	1.00	O
	33	CCC (P) => CTT (L)	X	O
	76	CAT (H) => TAT (Y)	1.00	O
	118	CCG (P) => TCG (S)	0.92	O
	133	CGT (R) => TGT (C)	1.00	O
	137	CCA (P) => CTA (L)	1.00	O
	140	GCC (A) => GTC (V)	0.92	X
	167	CCA (P) => CTA (L)	1.00	O
	178	CAC (H) => TAC (Y)	1.00	O
	244	CGG (R) => TGG (W)	1.00	O
	287	TCC (S) => TTC (F)	1.00	O
	303	CTC (L) => CTT (L)	X	O
	325	CAT (H) => TAT (Y)	1.00	O
	334	CAT (H) => TAT (Y)	1.00	O
	354	TTC (F) => TTT (F)	X	O
	358	CGG (R) => TGG (W)	1.00	O

nad7	1124	CCA (P) => CTA (L)	1.00	O
nad9	2	ACG (T) => ATG (M)	0.92	O
591	28	CCG (P) => TCG (S)	0.75	O
	41	TCA (S) => TTA (L)	1.00	O
	92	TCA (S) => TTA (L)	X	O
	113	CCA (P) => CTA (L)	0.92	O
	134	CCG (P) => CTG (L)	0.92	O
	158	TCC (S) => TTC (F)	0.33	O
	167	TCG (S) => TTG (L)	0.92	O
	178	CGC (R) => TGC (C)	0.92	O
	195	CCC (P) => CCT (P)	X	O
	218	GCG (A) => GTG (V)	0.92	O
	230	TCA (S) => TTA (L)	1.00	O
	233	CCA (P) => CTA (L)	1.00	O
	250	CCA (P) => TCA (S)	0.92	O
	298	CCG (P) => TCG (S)	0.83	O
	311	CCA (P) => CTA (L)	0.75	O
	328	CGG (R) => TGG (W)	1.00	O
	355	CCC (P) => TTC (F)	1.00	O
	356	CCC (P) => TTC (F)	1.00	O
	357	CCC (P) => TTT (F)	X	O
	368	TCC (S) => TTC (F)	1.00	O
	413	TCC (S) => TTC (F)	1.00	O
	440	TCC (S) => TTC (F)	1.00	O
	441	TCC (S) => TTT (F)	X	O

cob		403	CCT (P) => TTT (F)	1.00	O
		404	CCT (P) => TTT (F)	1.00	O
		442	CGG (R) => TGG (W)	1.00	O
		467	TCA (S) => TTA (L)	1.00	O
		514	CGG (R) => TGG (W)	1.00	O
		545	TCA (S) => TTA (L)	1.00	O
		568	CAT (H) => TAT (Y)	0.92	O
		575	CCC (P) => CTC (L)	1.00	O
		587	CCA (P) => CTA (L)	0.77	O
		620	TCG (S) => TTG (L)	1.00	O
		647	TCG (S) => TTG (L)	1.00	O
		680	TCT (S) => TTT (F)	1.00	O
		707	CCA (P) => CTA (L)	1.00	O
		715	CGG (R) => TGG (W)	1.00	O
		725	TCC (S) => TTC (F)	1.00	O
		726	TCC (S) => TTT (F)	X	O
		737	TCT (S) => TTT (F)	0.92	O
		770	TCG (S) => TTG (L)	1.00	O
		835	CGG (R) => TGG (W)	1.00	O
		845	CCA (P) => CTA (L)	1.00	O
		853	CAT (H) => TAT (Y)	1.00	O
		863	CCT (P) => CTT (L)	1.00	O
		908	CCA (P) => CTA (L)	1.00	O
		914	TCT (S) => TTT (F)	1.00	O
		932	CCA (P) => CTA (L)	1.00	O
		478	CCG (P) => TCG (S)	X	O
		533	TCT (S) => TTT (F)	1.00	O
rpl10		5	CCA (P) => CTA (L)	1.00	O
	483	74	TCA (S) => TTA (L)	X	O
		92	TCG (S) => TTG (L)	0.83	O
		116	CCC (P) => CTC (L)	1.00	O
		124	CCA (P) => TTA (L)	X	O
		125	CCA (P) => TTA (L)	0.83	O
		146	CCA (P) => CTA (L)	0.83	O
		215	TCC (S) => TTC (F)	1.00	X
		274	CCG (P) => TCG (S)	1.00	X
		290	TCG (S) => TTG (L)	1.00	X
		307	CGG (R) => TGG (W)	1.00	X
		335	CCA (P) => CTA (L)	0.83	X
		338	TCA (S) => TTA (L)	1.00	O
		425	TCT (S) => TTT (F)	X	O
		439	CAT (H) => TAT (Y)	0.83	X
		458	CCT (P) => CTT (L)	1.00	O
rpl16		5	CCA (P) => CTA (L)	1.00	O
	414	104	ACC (T) => ATC (I)	1.00	O
		105	ACC (T) => ATT (I)	X	O
		164	ACA (T) => ATA (I)	0.89	O
		196	CGG (R) => TGG (W)	1.00	O
		218	CCC (P) => CTC (L)	0.56	O
		293	GCG (A) => GTG (V)	1.00	O

cob	937	CCT (P) => TTT (F)	1.00	O
	938	CCT (P) => TTT (F)	1.00	O
	971	TCT (S) => TTT (F)	1.00	O
	997	CGG (R) => TGG (W)	1.00	O
	1015	CGC (R) => TGC (C)	1.00	O
	1019	TCA (S) => TTA (L)	1.00	O
	1030	CGG (R) => TGG (W)	1.00	O
	1047	CCC (P) => CCT (P)	X	O
	1091	GCT (A) => GTT (V)	0.46	X
	1097	TCC (S) => TTC (F)	1.00	O
	1105	CCC (P) => TTC (F)	1.00	O
	1106	CCC (P) => TTC (F)	1.00	O
	1124	CCG (P) => CTG (L)	1.00	O
	1148	ACC (T) => ATC (I)	0.23	X
	1168	CCA (P) => TTA (L)	X	O
	1169	CCA (P) => TTA (L)	X	O
	1191	ACC (T) => ACT (T)	X	O
cox1	16	CAG (Q) => TAG (X)	X	O
1596	23	TCT (S) => TTT (F)	X	O
	26	ACG (T) => ATG (M)	X	O
	47	CCG (P) => CTG (L)	0.89	O
	52	CCC (P) => TCC (S)	1.00	O
	54	CCC (P) => TCT (S)	X	O
	80	CCA (P) => CTA (L)	1.00	O
	82	CAT (H) => TAT (Y)	1.00	O
rpl16	297	TCC (S) => TCT (S)	X	O
	311	CCA (P) => CTA (L)	1.00	O
	383	TCG (S) => TTG (L)	0.75	O
	409	CAA (Q) => TAA (X)	1.00	O
rpl2	2	ACG (T) => ATG (M)	X	O
	32	CCT (P) => CTT (L)	1.00	O
	41	TCC (S) => TTC (F)	1.00	O
	76	CCA (P) => TCA (S)	1.00	O
	89	ACT (T) => ATT (I)	0.75	O
	143	TCG (S) => TTG (L)	1.00	O
	160	CCT (P) => TCT (S)	1.00	O
	367	CCT (P) => TCT (S)	1.00	X
	373	CTC (L) => TTC (F)	1.00	X
	412	CCT (P) => TCT (S)	0.50	X
	416	TCC (S) => TTC (F)	1.00	X
	595	CGG (R) => TGG (W)	1.00	O
	679	CCG (P) => TCG (S)	0.75	X
	722	TCA (S) => TTA (L)	0.25	O
	737	TCG (S) => TTG (L)	1.00	O
	758	ACG (T) => ATG (M)	0.75	O
	847	CTT (L) => TTT (F)	1.00	X
	974	CCG (P) => CTG (L)	1.00	O
	1004	TCA (S) => TTA (L)	1.00	O
	1070	CCG (P) => CTG (L)	1.00	O
	1100	ACA (T) => ATA (I)	1.00	O

cox1	86	TCA (S) => TTA (L)	0.33	O	*rpl2*	1123	CCA (P) => TCA (S)	1.00	O
	134	CCA (P) => CTA (L)	1.00	O		1142	TCA (S) => TTA (L)	1.00	O
	191	CCT (P) => CTT (L)	1.00	O		1147	CCG (P) => TCG (S)	1.00	O
	193	CAT (H) => TAT (Y)	1.00	O		1171	CCC (P) => TCC (S)	1.00	O
	203	TCA (S) => TTA (L)	1.00	O		1201	CCC (P) => TCC (S)	1.00	O
	221	TCT (S) => TTT (F)	1.00	O		1414	CAG (Q) => TAG (X)	X	O
	224	CCA (P) => CTA (L)	1.00	O	*rpl5*	9	ATC (I) => ATT (I)	X	O
	232	CCT (P) => TTT (F)	1.00	O	576	34	CAC (H) => TAC (Y)	1.00	O
	233	CCT (P) => TTT (F)	1.00	O		58	CCG (P) => TTG (L)	X	O
	266	TCT (S) => TTT (F)	1.00	O		59	CCG (P) => TTG (L)	1.00	O
	278	TCC (S) => TTC (F)	1.00	O		71	CCG (P) => CTG (L)	0.89	O
	290	CCT (P) => CTT (L)	0.89	O		76	CAC (H) => TAC (Y)	1.00	O
	334	CCA (P) => TCA (S)	1.00	O		89	ACG (T) => ATG (M)	1.00	X
	338	TCC (S) => TTC (F)	1.00	O		104	TCG (S) => TTG (L)	1.00	O
	340	CGG (R) => TGG (W)	1.00	O		136	CCA (P) => TCA (S)	0.50	O
	368	CCA (P) => CTA (L)	1.00	O		179	CCG (P) => CTG (L)	0.44	O
	376	CCA (P) => TCA (S)	1.00	O		181	CGC (R) => TGC (C)	0.89	O
	383	TCG (S) => TTG (L)	1.00	O		251	CCC (P) => CTC (L)	1.00	X
	461	GCT (A) => GTT (V)	1.00	O		335	TCC (S) => TTC (F)	1.00	O
	467	CCA (P) => CTA (L)	1.00	O		350	TCG (S) => TTG (L)	0.44	O
	476	TCT (S) => TTT (F)	1.00	O		409	CCG (P) => TCG (S)	0.67	O
	482	CCT (P) => CTT (L)	1.00	O		467	TCC (S) => TTC (F)	1.00	O
	509	TCA (S) => TTA (L)	1.00	O		500	GCA (A) => GTA (V)	0.89	X
	523	CTC (L) => TTC (F)	1.00	O		510	GCC (A) => GCT (A)	X	O
	538	CCC (P) => TTC (F)	1.00	O		533	CCA (P) => CTA (L)	0.89	O

cox1	539	CCC (P) => TTC (F)	1.00	O
	575	TCA (S) => TTA (L)	1.00	O
	589	CGG (R) => TGG (W)	1.00	O
	599	CCA (P) => CTA (L)	1.00	O
	623	TCA (S) => TTA (L)	1.00	O
	638	CCG (P) => CTG (L)	1.00	O
	662	TCA (S) => TTA (L)	1.00	O
	692	TCC (S) => TTC (F)	1.00	O
	736	CTT (L) => TTT (F)	1.00	O
	739	CGG (R) => TGG (W)	1.00	O
	746	TCC (S) => TTC (F)	1.00	O
	770	CCC (P) => CTC (L)	1.00	O
	776	CCG (P) => CTG (L)	1.00	O
	817	CCG (P) => TCG (S)	1.00	O
	842	CCA (P) => CTA (L)	1.00	O
	878	CCT (P) => CTT (L)	1.00	O
	884	TCT (S) => TTT (F)	1.00	O
	887	CCT (P) => CTT (L)	0.67	O
	892	CGG (R) => TGG (W)	1.00	O
	908	TCT (S) => TTT (F)	1.00	O
	920	TCA (S) => TTA (L)	1.00	O
	940	CAC (H) => TAC (Y)	1.00	O
	944	TCT (S) => TTT (F)	1.00	O
	992	TCT (S) => TTT (F)	1.00	O
	997	CGG (R) => TGG (W)	1.00	O
rpl5	551	TCG (S) => TTG (L)	1.00	O
rps10	2	ACG (T) => ATG (M)	1.00	O
342	16	CGC (R) => TGC (C)	0.40	X
	34	CCT (P) => TTT (F)	0.80	O
	35	CCT (P) => TTT (F)	0.80	O
	89	TCG (S) => TTG (L)	1.00	O
	158	TCT (S) => TTT (F)	1.00	O
	191	ACA (T) => ATA (I)	0.80	O
	229	CGG (R) => TGG (W)	0.60	O
	233	TCA (S) => TTA (L)	0.80	O
	280	CAC (H) => TAC (Y)	0.60	O
	293	TCG (S) => TTG (L)	1.00	O
rps11	178	CCG (P) => CTG (L)	X	O
582	238	CAT (H) => TAT (Y)	1.00	X
	242	TCC (S) => TTC (F)	X	O
	243	TCC (S) => TTT (F)	X	O
	244	CCA (P) => TTA (L)	X	O
	245	CCA (P) => TTA (L)	X	O
	247	CCA (P) => TCA (S)	1.00	O
	252	ATC (I) => ATT (I)	X	O
	266	TCT (S) => TTT (F)	X	O
	275	GCG (A) => GTG (V)	X	O
	323	TCG (S) => TTG (L)	X	O
	418	CCA (P) => TCA (S)	0.33	X
	451	CTC (L) => TTC (F)	X	O

cox1	1049	TCT (S) => TTT (F)	1.00	O
	1055	GCA (A) => GTA (V)	1.00	O
	1061	TCC (S) => TTC (F)	1.00	O
	1070	CCG (P) => CTG (L)	1.00	O
	1073	TCC (S) => TTC (F)	1.00	O
	1111	CCT (P) => TCT (S)	0.89	O
	1130	CCG (P) => CTG (L)	1.00	O
	1141	CAT (H) => TAT (Y)	1.00	O
	1160	TCC (S) => TTC (F)	1.00	O
	1174	CCT (P) => TCT (S)	1.00	O
	1196	TCA (S) => TTA (L)	1.00	O
	1199	TCT (S) => TTT (F)	1.00	O
	1208	TCT (S) => TTT (F)	1.00	O
	1210	CAC (H) => TAC (Y)	1.00	O
	1216	CGG (R) => TGG (W)	1.00	O
	1231	CCT (P) => TTT (F)	0.67	O
	1232	CCT (P) => TTT (F)	0.67	O
	1270	CTT (L) => TTT (F)	1.00	O
	1273	CGG (R) => TGG (W)	1.00	O
	1282	CTT (L) => TTT (F)	1.00	O
	1298	TCG (S) => TTG (L)	0.89	O
	1402	CCT (P) => TCT (S)	1.00	O
	1426	CGT (R) => TGT (C)	0.67	O
	1429	CGT (R) => TGT (C)	0.78	O
rps11	476	CCG (P) => CTG (L)	X	O
	482	TCT (S) => TTT (F)	X	O
rps12	11	TCG (S) => TTG (L)	0.29	O
378	20	TCG (S) => TTG (L)	0.94	O
	71	TCG (S) => TTG (L)	0.94	O
	100	CGC (R) => TGC (C)	1.00	O
	104	CCG (P) => CTG (L)	1.00	O
	112	CCG (P) => TCG (S)	0.88	O
	139	CCA (P) => TCA (S)	1.00	O
	146	CCA (P) => CTA (L)	1.00	O
	221	CCG (P) => CTG (L)	0.88	O
	232	CCT (P) => TCT (S)	1.00	O
	242	TCG (S) => TTG (L)	0.88	O
	278	GCG (A) => GTG (V)	1.00	O
	289	CGT (R) => TGT (C)	0.88	O
	311	TCG (S) => TTG (L)	1.00	O
	320	ACT (T) => ATT (I)	0.94	O
rps13	5	TCA (S) => TTA (L)	0.60	O
489	7	CAT (H) => TAT (Y)	1.00	X
	56	CCA (P) => CTA (L)	0.90	O
	92	ACT (T) => ATT (I)	0.90	X
	100	CGT (R) => TGT (C)	0.90	O
	143	TCA (S) => TTA (L)	1.00	O
	190	CAT (H) => TAT (Y)	X	O

cox1	1494	CCC (P) => CCT (P)	X	O
	1495	CGG (R) => TGG (W)	1.00	O
	1513	CCA (P) => TCA (S)	1.00	X
	1523	CCG (P) => CTG (L)	1.00	O
	1552	CTT (L) => TTT (F)	1.00	O
	1561	CCT (P) => TTT (F)	1.00	O
	1562	CCT (P) => TTT (F)	1.00	O
cox2	25	CTC (L) => TTC (F)	X	O
807	71	TCT (S) => TTT (F)	1.00	O
	161	TCA (S) => TTA (L)	0.95	O
	253	CGG (R) => TGG (W)	1.00	O
	263	TCT (S) => TTT (F)	0.95	O
	278	CCG (P) => CTG (L)	1.00	O
	308	CCG (P) => CTG (L)	1.00	O
	373	CGG (R) => TGG (W)	1.00	O
	379	CGG (R) => TGG (W)	1.00	O
	425	CCA (P) => CTA (L)	1.00	O
	443	ACG (T) => ATG (M)	1.00	O
	461	TCA (S) => TTA (L)	1.00	O
	476	TCA (S) => TTA (L)	1.00	O
	554	GCA (A) => GTA (V)	1.00	O
	565	CGG (R) => TGG (W)	1.00	O
	577	CCC (P) => TCC (S)	1.00	O
	581	TCA (S) => TTA (L)	1.00	O
rps13	349	CCG (P) => TCG (S)	1.00	X
rps14	47	GCG (A) => GTG (V)	0.60	X
303	74	CCT (P) => CTT (L)	0.40	O
	76	CAT (H) => TAT (Y)	1.00	O
	104	CCT (P) => CTT (L)	0.80	O
	127	CAT (H) => TAT (Y)	X	O
	142	CCT (P) => TCT (S)	0.60	O
	149	TCG (S) => TTG (L)	1.00	O
	163	CCC (P) => TCC (S)	1.00	O
	235	CCT (P) => TCT (S)	0.80	O
	248	TCT (S) => TTT (F)	1.00	O
	275	TCG (S) => TTG (L)	1.00	O
	298	CGG (R) => TGG (W)	1.00	O
rps19	2	GCG (A) => GTG (V)	X	O
282	36	GTC (V) => GTT (V)	X	O
	47	TCG (S) => TTG (L)	1.00	O
	50	TCG (S) => TTG (L)	0.67	X
	94	CGG (R) => TGG (W)	1.00	O
	109	CCC (P) => TCC (S)	0.50	O
	125	TCT (S) => TTT (F)	1.00	O
	163	CCT (P) => TTT (F)	1.00	O
	164	CCT (P) => TTT (F)	1.00	O
	167	GCT (A) => GTT (V)	1.00	O
	206	TCT (S) => TTT (F)	0.83	O

cox2	614	TCG (S) => TTG (L)	1.00	O
	632	TCG (S) => TTG (L)	0.84	O
	655	CAC (H) => TAC (Y)	1.00	O
	676	CGT (R) => TGT (C)	1.00	O
	695	TCT (S) => TTT (F)	X	O
	698	ACG (T) => ATG (M)	X	O
	766	CCA (P) => TCA (S)	0.68	X
cox3	149	TCG (S) => TTG (L)	1.00	O
798	156	TTC (F) => TTT (F)	X	O
	161	CCA (P) => CTA (L)	0.92	O
	174	TTC (F) => TTT (F)	X	O
	178	CGG (R) => TGG (W)	1.00	O
	245	CCT (P) => CTT (L)	0.92	O
	263	CCG (P) => CTG (L)	1.00	O
	266	TCC (S) => TTC (F)	1.00	O
	270	ATC (I) => ATT (I)	X	O
	289	CTC (L) => TTC (F)	0.92	O
	298	CTT (L) => TTT (F)	1.00	O
	301	CCT (P) => TTT (F)	1.00	O
	302	CCT (P) => TTT (F)	1.00	O
	304	CGG (R) => TGG (W)	1.00	O
	311	TCT (S) => TTT (F)	0.92	O
	322	CCT (P) => TCT (S)	1.00	O
	326	TCG (S) => TTG (L)	1.00	O
	355	CGG (R) => TGG (W)	1.00	O
rps19	219	GCC (A) => GCT (A)	X	O
	241	CCG (P) => TCG (S)	0.83	O
rps1	43	CCC (P) => TCC (S)	1.00	O
690	50	TCT (S) => TTT (F)	1.00	O
	52	CCC (P) => TTC (F)	1.00	X
	53	CCC (P) => TTC (F)	1.00	X
	82	CCA (P) => TCA (S)	X	O
	95	TCA (S) => TTA (L)	1.00	O
	113	CCG (P) => CTG (L)	1.00	O
	128	CCT (P) => CTT (L)	0.67	O
	145	CGT (R) => TGT (C)	0.67	O
	209	TCC (S) => TTC (F)	X	O
	212	CCG (P) => CTG (L)	0.33	O
	236	CCG (P) => CTG (L)	1.00	X
	338	TCG (S) => TTG (L)	0.33	O
	350	TCT (S) => TTT (F)	1.00	O
	359	CCA (P) => CTA (L)	1.00	O
	377	CCG (P) => CTG (L)	1.00	X
	497	CCG (P) => CTG (L)	0.67	O
	536	TCT (S) => TTT (F)	1.00	O
	542	ACT (T) => ATT (I)	1.00	O
	565	CCA (P) => TCA (S)	X	O
	566	CCA (P) => CTA (L)	0.67	X
	569	GCA (A) => GTA (V)	1.00	O
	587	ACA (T) => ATA (I)	0.67	O

cox3	380	TCA (S) => TTA (L)	1.00	O
	413	CCT (P) => CTT (L)	0.83	O
	419	CCC (P) => CTC (L)	1.00	O
	424	CCA (P) => TCA (S)	1.00	O
	497	TCA (S) => TTA (L)	0.92	O
	512	TCA (S) => TTA (L)	0.75	X
	550	CAT (H) => TAT (Y)	1.00	O
	565	CCC (P) => TTC (F)	0.92	O
	566	CCC (P) => TTC (F)	0.92	O
	586	CAT (H) => TAT (Y)	1.00	O
	601	CCC (P) => TTC (F)	1.00	O
	602	CCC (P) => TTC (F)	1.00	O
	605	TCA (S) => TTA (L)	1.00	O
	650	TCC (S) => TTC (F)	1.00	O
	653	CCA (P) => CTA (L)	1.00	O
	661	CGT (R) => TGT (C)	1.00	O
	733	CGG (R) => TGG (W)	1.00	O
	740	TCC (S) => TTC (F)	1.00	O
	754	CGG (R) => TGG (W)	0.92	O
	758	TCA (S) => TTA (L)	1.00	O
	764	CCA (P) => CTA (L)	0.92	O
	772	CCT (P) => TCT (S)	1.00	O
matR	71	CCC (P) => CTC (L)	1.00	O
2190	77	TCC (S) => TTC (F)	0.62	X
	98	CCG (P) => CTG (L)	X	O
rps1	592	CCC (P) => TTC (F)	1.00	O
	593	CCC (P) => TTC (F)	1.00	O
	594	CCC (P) => TTT (F)	X	O
	596	CCT (P) => CTT (L)	1.00	O
	608	CCT (P) => CTT (L)	X	O
	641	TCC (S) => TTC (F)	1.00	O
	642	TCC (S) => TTT (F)	X	O
	644	ACC (T) => ATC (I)	X	O
	671	ACG (T) => ATG (M)	X	O
rps2	10	CCC (P) => TCC (S)	0.43	X
720	14	CCC (P) => CTC (L)	0.67	O
	38	CCG (P) => CTG (L)	1.00	O
	83	TCC (S) => TTC (F)	0.71	X
	104	TCC (S) => TTC (F)	X	O
	125	CCC (P) => CTC (L)	0.57	O
	130	CCA (P) => TCA (S)	0.57	O
	152	TCA (S) => TTA (L)	0.86	O
	170	TCC (S) => TTC (F)	0.86	O
	171	TCC (S) => TTT (F)	X	O
	182	CCC (P) => CTC (L)	0.43	O
	209	TCC (S) => TTC (F)	0.71	O
	230	TCC (S) => TTC (F)	1.00	O
	254	GCG (A) => GTG (V)	X	O
	323	TCG (S) => TTG (L)	0.25	X
	346	CCG (P) => TCG (S)	0.75	X

matR	118	CCG (P) => TCG (S)	0.88	X
	283	CTC (L) => TTC (F)	0.62	X
	287	ACC (T) => ATC (I)	0.75	X
	340	CCT (P) => TCT (S)	1.00	X
	389	CCA (P) => CTA (L)	1.00	O
	400	CAT (H) => TAT (Y)	1.00	O
	404	CCA (P) => CTA (L)	1.00	O
	445	CAC (H) => TAC (Y)	0.75	X
	701	TCC (S) => TTC (F)	1.00	X
	743	CCC (P) => CTC (L)	1.00	O
	869	CCT (P) => CTT (L)	0.88	X
	1009	CAC (H) => TAC (Y)	1.00	O
	1034	CCA (P) => CTA (L)	1.00	O
	1109	CCG (P) => CTG (L)	1.00	O
	1169	TCC (S) => TTC (F)	1.00	O
	1172	CCC (P) => CTC (L)	1.00	O
	1250	GCA (A) => GTA (V)	1.00	X
	1745	ACC (T) => ATC (I)	1.00	X
	1792	CGG (R) => TGG (W)	1.00	O
	1817	CCC (P) => CTC (L)	1.00	O
	1825	CAC (H) => TAC (Y)	1.00	X
	1828	CAT (H) => TAT (Y)	1.00	O
	1846	CTC (L) => TTC (F)	X	O
	1885	CGG (R) => TGG (W)	1.00	O
	1898	TCC (S) => TTC (F)	1.00	O

rps2		421	CGT (R) => TGT (C)	1.00	O
		428	GCA (A) => GTA (V)	0.86	O
		584	CCA (P) => CTA (L)	1.00	O
		587	TCT (S) => TTT (F)	1.00	O
		589	CGT (R) => TGT (C)	1.00	O
		596	TCG (S) => TTG (L)	1.00	O
		614	CCT (P) => CTT (L)	X	O
		617	CCT (P) => CTT (L)	0.71	O
rps3		2	ACG (T) => ATG (M)	1.00	O
	1668	58	CCA (P) => TCA (S)	1.00	O
		64	CGG (R) => TGG (W)	1.00	O
		75	GAC (D) => GAT (D)	X	O
		79	CAT (H) => TAT (Y)	1.00	O
		114	CTC (L) => CTT (L)	X	O
		121	CAT (H) => TAT (Y)	1.00	O
		194	TCT (S) => TTT (F)	1.00	X
		221	TCT (S) => TTT (F)	1.00	O
		462	GAC (D) => GAT (D)	X	O
		497	TCA (S) => TTA (L)	0.71	O
		587	CCG (P) => CTG (L)	0.86	X
		604	CCC (P) => TCC (S)	0.83	X
		608	TCC (S) => TTC (F)	1.00	X
		609	TCC (S) => TCT (S)	X	O
		627	TTC (F) => TTT (F)	X	O
		634	CTT (L) => TTT (F)	0.83	X

matR	1904	CCT (P) => CTT (L)	1.00	O
	1943	CCA (P) => CTA (L)	0.88	O
	1961	TCA (S) => TTA (L)	0.88	O
	2098	CAG (Q) => TAG (X)	0.50	X
mttB	2	ACG (T) => ATG (M)	0.38	O
	14	TCG (S) => TTG (L)	0.88	O
	26	CCG (P) => CTG (L)	0.88	X
	35	GCT (A) => GTT (V)	0.75	X
	52	CGG (R) => TGG (W)	0.88	X
	74	TCG (S) => TTG (L)	0.62	O
	88	CGT (R) => TGT (C)	0.88	X
	91	CAT (H) => TAT (Y)	1.00	O
	100	CCG (P) => TCG (S)	1.00	X
	110	TCA (S) => TTA (L)	0.50	O
	119	CCA (P) => CTA (L)	1.00	O
	122	TCA (S) => TTA (L)	1.00	O
	134	TCT (S) => TTT (F)	0.88	O
	137	CCG (P) => CTG (L)	1.00	O
	154	CCG (P) => TCG (S)	0.62	O
	161	TCC (S) => TTC (F)	1.00	O
	162	TCC (S) => TTT (F)	X	O
	166	CGT (R) => TGT (C)	1.00	O
	176	TCG (S) => TTG (L)	0.75	O
	188	TCT (S) => TTT (F)	0.62	O
	191	TCA (S) => TTA (L)	X	O
rps3	637	CTC (L) => TTC (F)	1.00	X
	659	ACG (T) => ATG (M)	X	O
	725	TCG (S) => TTG (L)	0.86	O
	740	TCC (S) => TTC (F)	0.57	O
	767	TCT (S) => TTT (F)	1.00	O
	796	CAT (H) => TAT (Y)	X	O
	867	TCC (S) => TCT (S)	X	O
	1010	CCG (P) => CTG (L)	1.00	O
	1090	CCA (P) => TCA (S)	X	O
	1091	CCA (P) => CTA (L)	1.00	X
	1094	CCA (P) => TCA (S)	X	O
	1121	TCA (S) => TTA (L)	0.86	O
	1145	CCG (P) => CTG (L)	0.29	O
	1344	ATC (I) => ATT (I)	X	O
	1382	CCA (P) => CTA (L)	1.00	O
	1409	CCG (P) => CTG (L)	0.86	O
	1441	CTT (L) => TTT (F)	1.00	O
	1510	CCA (P) => TCA (S)	1.00	O
	1520	TCG (S) => TTG (L)	1.00	O
	1580	GCA (A) => GTA (V)	1.00	X
	1583	TCT (S) => TTT (F)	0.86	O
	1600	CAC (H) => TAC (Y)	1.00	O
	1607	TCT (S) => TTT (F)	X	O
	1651	CGG (R) => TGG (W)	1.00	O
rps4	2	ACG (T) => ATG (M)	X	O

mttB	212	CCA (P) => CTA (L)	0.50	O	1053	86	CCC (P) => CTC (L)	1.00	O
	224	TCC (S) => TTC (F)	X	O		95	TCG (S) => TTG (L)	0.83	O
	230	TCC (S) => TTC (F)	1.00	O		133	CCG (P) => TCG (S)	0.67	X
	242	TCC (S) => TTC (F)	0.50	O		134	CCG (P) => CTG (L)	X	O
	265	CCT (P) => TTT (F)	1.00	O		182	TCG (S) => TTG (L)	1.00	O
	266	CCT (P) => TTT (F)	1.00	O		184	CCC (P) => TCC (S)	0.83	O
	269	TCG (S) => TTG (L)	0.75	O		191	TCT (S) => TTT (F)	0.83	O
	274	ATC (I) => ATT (I)	X	O		193	CAT (H) => TAT (Y)	1.00	O
	319	CAC (H) => TAC (Y)	0.62	X		244	CAT (H) => TAT (Y)	1.00	O
	323	CCA (P) => CTA (L)	1.00	O		257	CCA (P) => CTA (L)	1.00	O
	334	CGC (R) => TGC (C)	0.88	O		266	CCA (P) => CTA (L)	0.83	O
	344	CCG (P) => CTG (L)	0.88	O		278	TCG (S) => TTG (L)	0.67	O
	361	CTT (L) => TTT (F)	X	O		290	CCG (P) => CTG (L)	0.83	O
	367	CGG (R) => TGG (W)	0.62	O		307	CGT (R) => TGT (C)	0.50	O
	385	CGG (R) => TGG (W)	1.00	O		335	CCG (P) => CTG (L)	1.00	O
	395	CCA (P) => CTA (L)	0.75	O		355	CGT (R) => TGT (C)	1.00	X
	404	GCG (A) => GTG (V)	0.62	X		359	GCG (A) => GTG (V)	0.83	O
	460	CAT (H) => TAT (Y)	0.88	O		389	TCT (S) => TTT (F)	0.50	O
	470	TCA (S) => TTA (L)	0.88	O		410	CCA (P) => CTA (L)	0.33	O
	485	TCG (S) => TTG (L)	0.88	O		435	GTC (V) => GTT (V)	X	O
	529	CGT (R) => TGT (C)	0.88	O		467	TCC (S) => TTC (F)	1.00	O
	536	CCA (P) => CTA (L)	0.62	O		469	CAT (H) => TAT (Y)	1.00	O
	563	TCC (S) => TTC (F)	0.75	O		482	TCA (S) => TTA (L)	1.00	O
	593	TCC (S) => TTC (F)	0.88	X		497	GCG (A) => GTG (V)	X	O
	594	TCC (S) => TCT (S)	X	O		509	CCA (P) => CTA (L)	0.83	O

mttB	595	CCG (P) => TCG (S)	1.00	O
	602	CCC (P) => CTC (L)	0.62	O
	605	ACA (T) => ATA (I)	X	O
	652	CGT (R) => TGT (C)	0.62	O
	659	CCT (P) => CTT (L)	0.50	O
	668	CCG (P) => CTG (L)	0.50	O
	687	ATC (I) => ATT (I)	X	O
	689	TCT (S) => TTT (F)	1.00	O
	698	TCG (S) => TTG (L)	0.75	O
nad1	2	ACG (T) => ATG (M)	0.80	O
981	29	CCT (P) => CTT (L)	1.00	O
	49	CTA (L) => TTA (L)	X	O
	97	CTC (L) => TTC (F)	0.70	O
	134	TCG (S) => TTG (L)	X	O
	215	TCT (S) => TTT (F)	0.90	O
	221	TCT (S) => TTT (F)	1.00	O
	245	TCT (S) => TTT (F)	1.00	O
	265	CGG (R) => TGG (W)	1.00	O
	286	CAT (H) => TAT (Y)	1.00	O
	299	TCG (S) => TTG (L)	0.90	O
	301	CCA (P) => TCA (S)	1.00	O
	308	TCG (S) => TTG (L)	1.00	O
	325	CAT (H) => TAT (Y)	1.00	O
	332	TCT (S) => TTT (F)	1.00	O
	381	TCC (S) => TCT (S)	X	O
rps4	517	CCG (P) => TCG (S)	X	O
	589	CTA (L) => TTA (L)	X	O
	593	CCA (P) => CTA (L)	1.00	O
	608	TCG (S) => TTG (L)	1.00	O
	658	CCC (P) => TCC (S)	X	O
	660	CCC (P) => TCT (S)	X	O
	662	TCT (S) => TTT (F)	1.00	X
	668	TCA (S) => TTA (L)	X	O
	725	TCG (S) => TTG (L)	0.67	O
	730	CAT (H) => TAT (Y)	X	O
	734	TCT (S) => TTT (F)	0.75	X
	782	CCT (P) => CTT (L)	0.80	X
	821	TCA (S) => TTA (L)	0.60	O
	845	TCT (S) => TTT (F)	X	O
	876	ATC (I) => ATT (I)	X	O
	911	TCG (S) => TTG (L)	0.83	O
	922	CAT (H) => TAT (Y)	0.83	O
	932	CCG (P) => CTG (L)	0.67	O
	947	TCT (S) => TTT (F)	1.00	O
	998	CCG (P) => CTG (L)	0.83	O
	1007	CCC (P) => CTC (L)	1.00	O
	1012	CGG (R) => TGG (W)	1.00	O
rps7	143	ACC (T) => ATC (I)	0.38	O
588	185	TCA (S) => TTA (L)	0.75	O
	200	ACG (T) => ATG (M)	1.00	O

nad1	388	CCG (P) => TCG (S)	1.00	O
	404	CCA (P) => CTA (L)	1.00	O
	476	CCA (P) => CTA (L)	0.90	O
	490	CCT (P) => TCT (S)	1.00	O
	493	CGT (R) => TGT (C)	1.00	O
	555	CCC (P) => CCT (P)	X	O
	580	CGT (R) => TGT (C)	1.00	O
	584	CCA (P) => CTA (L)	1.00	O
	607	CTT (L) => TTT (F)	1.00	O
	635	TCA (S) => TTA (L)	1.00	O
	653	GCA (A) => GTA (V)	1.00	O
	674	TCT (S) => TTT (F)	0.90	O
	680	CCT (P) => CTT (L)	1.00	O
	683	TCT (S) => TTT (F)	1.00	O
	734	TCG (S) => TTG (L)	0.90	O
	739	CCT (P) => TTT (F)	1.00	O
	740	CCT (P) => TTT (F)	1.00	O
	743	CCA (P) => CTA (L)	1.00	O
	751	CGG (R) => TGG (W)	1.00	O
	755	CCG (P) => CTG (L)	1.00	O
	764	CCA (P) => CTA (L)	0.80	O
	779	TCC (S) => TTC (F)	0.90	X
	827	TCC (S) => TTC (F)	1.00	O
	830	CCG (P) => CTG (L)	1.00	O
	834	TTC (F) => TTT (F)	X	O

rps7	224	GCT (A) => GTT (V)	0.62	X
	236	GCT (A) => GTT (V)	0.88	X
	247	CTC (L) => TTC (F)	0.75	X
	257	CCA (P) => CTA (L)	0.75	O
	281	ACC (T) => ATC (I)	1.00	X
	356	ACT (T) => ATT (I)	0.62	X
	358	CAT (H) => TAT (Y)	0.62	O
	461	TCA (S) => TTA (L)	0.88	O
	476	TCT (S) => TTT (F)	0.62	X
	488	CCG (P) => CTG (L)	0.88	O
	536	CCT (P) => CTT (L)	1.00	O
	545	CCG (P) => CTG (L)	1.00	O
	550	CCC (P) => TCC (S)	0.62	O
sdh3	26	ACG (T) => ATG (M)	1.00	X
564	56	CCT (P) => CTT (L)	0.75	O
	64	CAC (H) => TAC (Y)	1.00	O
	82	CCG (P) => TCG (S)	1.00	O
	91	CCA (P) => TCA (S)	1.00	O
	109	CCC (P) => TCC (S)	0.86	O
	137	TCG (S) => TTG (L)	0.86	O
	145	CCC (P) => TTC (F)	0.20	X
	146	CCC (P) => TTC (F)	0.20	O
	149	CCT (P) => CTT (L)	0.57	O
	157	CCG (P) => TTG (L)	X	O
	158	CCG (P) => TTG (L)	0.57	O

nad1	884	TCA (S) => TTA (L)	1.00	O
	893	CCT (P) => CTT (L)	1.00	O
	898	CGG (R) => TGG (W)	1.00	O
	928	CGG (R) => TGG (W)	1.00	O
	938	TCC (S) => TTC (F)	X	O
	939	TCC (S) => TTT (F)	X	O
	953	CCA (P) => CTA (L)	1.00	O
	976	CAA (Q) => TAA (X)	0.90	X
nad2	16	CCA (P) => TTA (L)	X	O
1473	17	CCA (P) => TTA (L)	0.89	O
	41	CCC (P) => CTC (L)	0.44	O
	116	TCA (S) => TTA (L)	0.89	O
	119	GCA (A) => GTA (V)	0.67	X
	146	CCC (P) => CTC (L)	1.00	O
	147	CCC (P) => CTT (L)	X	O
	214	CGG (R) => TGG (W)	0.67	O
	242	TCT (S) => TTT (F)	0.89	O
	266	CCA (P) => CTA (L)	1.00	O
	335	TCC (S) => TTC (F)	1.00	O
	336	TCC (S) => TTT (F)	X	O
	341	TCC (S) => TTC (F)	0.67	O
	345	ATC (I) => ATT (I)	X	O
	350	TCA (S) => TTA (L)	1.00	O
	359	CCT (P) => CTT (L)	0.89	O
	361	CCT (P) => TCT (S)	1.00	O
sdh3	178	CTC (L) => TTC (F)	0.57	O
	180	CTC (L) => TTT (F)	X	O
	193	CTC (L) => TTC (F)	1.00	O
	202	CTT (L) => TTT (F)	0.71	X
	228	TTC (F) => TTT (F)	X	O
	230	CCG (P) => CTG (L)	0.33	X
	239	ACT (T) => ATT (I)	0.86	X
	263	TCA (S) => TTA (L)	X	O
	272	CCG (P) => CTG (L)	0.86	O
	274	CGC (R) => TGC (C)	0.43	O
	326	TCC (S) => TTC (F)	0.83	X
	332	TCC (S) => TTC (F)	0.33	X
	337	CCA (P) => TCA (S)	0.67	X
	361	CCT (P) => TTT (F)	0.25	X
	362	CCT (P) => TTT (F)	0.25	O
	365	ACG (T) => ATG (M)	1.00	X
	383	TCA (S) => TTA (L)	X	O
	390	TTC (F) => TTT (F)	X	O
	418	CGA (R) => TGA (X)	X	O
sdh4	8	CCG (P) => CTG (L)	X	O
396	15	TTC (F) => TTT (F)	X	O
	29	TCG (S) => TTG (L)	X	O
	33	GTC (V) => GTT (V)	X	O
	39	CCC (P) => CCT (P)	X	O
	47	CCC (P) => CTC (L)	X	O

nad2	367	CGC (R) => TGC (C)	1.00	O	*sdh4*	83	ACA (T) => ATA (I)	0.43	X
	374	ACG (T) => ATG (M)	1.00	O		152	TCC (S) => TTC (F)	0.75	X
	388	CCG (P) => TCG (S)	1.00	O		155	CCA (P) => CTA (L)	0.88	O
	394	CAT (H) => TAT (Y)	1.00	O		189	TCC (S) => TCT (S)	X	O
	401	TCA (S) => TTA (L)	1.00	O		200	CCG (P) => CTG (L)	0.75	O
	428	CCT (P) => CTT (L)	1.00	O		217	CGG (R) => TGG (W)	1.00	O
	437	TCA (S) => TTA (L)	1.00	O		256	CAT (H) => TAT (Y)	0.88	O
	481	CCC (P) => TCC (S)	1.00	O		305	TCG (S) => TTG (L)	0.62	X
	497	TCG (S) => TTG (L)	1.00	O		310	CTT (L) => TTT (F)	X	O
	506	TCG (S) => TTG (L)	0.67	O		343	CCC (P) => TTC (F)	X	O
	523	CCC (P) => TCC (S)	1.00	O		344	CCC (P) => TTC (F)	0.50	O
	526	CCT (P) => TCT (S)	1.00	O		345	CCC (P) => TTT (F)	X	O
	536	TCA (S) => TTA (L)	0.89	O		347	GCT (A) => GTT (V)	1.00	O
	543	TTC (F) => TTT (F)	X	O		356	CCG (P) => CTG (L)	X	O
	609	ACC (T) => ACT (T)	X	O					
	626	TCG (S) => TTG (L)	0.50	O					
	683	TCT (S) => TTT (F)	1.00	O					
	696	ATC (I) => ATT (I)	X	O					
	774	ATC (I) => ATT (I)	X	O					
	815	TCT (S) => TTT (F)	1.00	O					
	820	CAT (H) => TAT (Y)	0.56	O					
	859	CTC (L) => TTC (F)	1.00	O					
	862	CGC (R) => TGC (C)	0.89	O					
	874	CCT (P) => TCT (S)	1.00	O					
	934	CAT (H) => TAT (Y)	1.00	O					

nad2	964	CGT (R) => TGT (C)	0.78	O
	968	ACC (T) => ATC (I)	1.00	O
	1027	CAT (H) => TAT (Y)	1.00	O
	1034	TCA (S) => TTA (L)	0.78	O
	1052	TCC (S) => TTC (F)	1.00	O
	1064	CCA (P) => CTA (L)	1.00	O
	1112	CCA (P) => CTA (L)	1.00	O
	1162	CCA (P) => TCA (S)	1.00	O
	1195	CGT (R) => TGT (C)	1.00	O
	1214	TCC (S) => TTC (F)	1.00	O
	1217	TCC (S) => TTC (F)	1.00	O
	1247	CCA (P) => CTA (L)	1.00	O
	1282	CGT (R) => TGT (C)	1.00	O
	1304	GCG (A) => GTG (V)	1.00	O
	1318	CCC (P) => TTC (F)	1.00	O
	1319	CCC (P) => TTC (F)	1.00	O
	1394	TCT (S) => TTT (F)	X	O
	1397	TCC (S) => TTC (F)	0.89	O
	1398	TCC (S) => TTT (F)	X	O
	1406	TCA (S) => TTA (L)	0.67	O
	1409	TCC (S) => TTC (F)	1.00	O
	1415	CCG (P) => CTG (L)	1.00	O
	1422	CCC (P) => CCT (P)	X	O
	1430	TCG (S) => TTG (L)	1.00	O
	1451	ACG (T) => ATG (M)	1.00	O

Table S8. Predicted repeat pairs in *Abies koreana* plastid genome.

Repeat	Length	Start	End	Direction
Repeat_1	1186	76335	77520	plus
Repeat_1	1186	121341	120156	minus
Repeat_2	151	67585	67735	plus
Repeat_2	151	67623	67773	plus
Repeat_3	139	13793	13931	plus
Repeat_3	139	68361	68223	minus
Repeat_4	113	67585	67697	plus
Repeat_4	113	67623	67735	plus
Repeat_4	113	67661	67773	plus
Repeat_5	102	30979	31080	plus
Repeat_5	102	98082	98183	plus
Repeat_6	80	43637	43716	plus
Repeat_6	80	43685	43764	plus
Repeat_7	75	67585	67659	plus
Repeat_7	75	67623	67697	plus
Repeat_7	75	67661	67735	plus
Repeat_7	75	67699	67773	plus
Repeat_8	73	55560	55632	plus
Repeat_8	73	67327	67399	plus
Repeat_9	53	47852	47904	plus
Repeat_9	53	47876	47928	plus
Repeat_10	52	31133	31184	plus
Repeat_10	52	98238	98289	plus
Repeat_11	46	119387	119432	plus
Repeat_11	46	119432	119387	minus
Repeat_12	44	90736	90779	plus
Repeat_12	44	90779	90736	minus
Repeat_13	42	13934	13975	plus
Repeat_13	42	68220	68179	minus
Repeat_14	41	43590	43630	plus
Repeat_14	41	43611	43651	plus
Repeat_15	39	96786	96824	plus
Repeat_15	39	96825	96863	plus
Repeat_16	37	67585	67621	plus
Repeat_16	37	67623	67659	plus
Repeat_16	37	67661	67697	plus
Repeat_16	37	67699	67735	plus
Repeat_16	37	67737	67773	plus
Repeat_17	37	47754	47790	plus
Repeat_17	37	47826	47862	plus
Repeat_18	34	100535	100568	plus
Repeat_18	34	100568	100535	minus
Repeat_19	32	43637	43668	plus
Repeat_19	32	43685	43716	plus
Repeat_19	32	43733	43764	plus

Table S9. Summary of *Abies* plastid genomes.

Taxon	Plastome size (bp)	LSC (bp)	SSC (bp)	IR (bp)	GC content (%)	Protein genes	tRNA genes	rRNA genes	GenBank number
A. kawakamii	121,803	76,339	43,104	1,180	38.3	72	35	4	MZ868721
A. nephrolepis	121,221	76,266	42,583	1,186	38.3	72	34	4	MZ504153
A. pindrow	119,981	76,270	41,343	1,184	38.3	72	34	4	MZ868720
A. sachalinensis	121,333	76,256	42,705	1,186	38.3	72	34	4	MZ504158
A. sibirica	121,256	76,274	42,622	1,180	38.3	72	34	4	MZ868722
A. spectabilis	119,914	76,310	41,260	1,172	38.3	72	34	4	MZ868723
A. veitchii	121,371	76,291	42,708	1,186	38.2	72	34	4	MZ504157
A. firma	121,282	76,322	42,588	1,186	38.2	72	34	4	SRR12710828
A. alba	121,243	75,783	43,108	1,176	38.3	72	34	4	NC_042410
A. balsamea	121,574	76,039	43,165	1,185	38.2	72	34	4	NC_042778
A. beshanzuensis	121,399	76,398	42641	1,180	38.3	72	34	4	NC_045884
A. beshanzuensis var. *ziyuanensis*	121,274	76,317	42,597	1,180	38.3	72	34	4	MH706705
A. chensiensis	121,329	76,291	42,678	1,180	38.3	72	34	4	NC_041464
A. concolor	120,427				38.3	72	34	4	NC_039581

A. delavayi	120,141	76,332	41,449	1,180	38.3	72	34	4	NC_057313
A. delavayi subsp. *fansipanensis*	120,094	76,386	43,360	1,174	38.3	72	34	4	MK607416
A. ernestii	121,841	76,359	43,122	1,180	38.3	72	34	4	MH706707
A. ernestii var. *salouenensis*	121,681	76,199	43,122	1,180	38.3	72	34	4	ON184013
A. fabri	120,027	76,311	41,356	1,180	38.3	72	34	4	NC_057314
A. fanjingshanensis	120,057	76,353	41,356	1,174	38.3	72	34	4	NC_042777
A. fargesii	121,799	76,334	43,113	1,176	38.3	72	34	4	NC_042775
A. ferreana	120,049	76,341	41,348	1,180	38.3	72	34	4	NC_062889
A. forrestii	120,022	76,309	43,353	1,180	38.3	72	34	4	MH706715
A. georgei var. *smithii*	121,191	76,274	42,557	1,180	38.3	72	34	4	NC_054152
A. nukiangensis	120,017	76,311	41,358	1,174	38.3	72	34	4	NC_057315
A. religiosa	119,423				38.4	72	34	4	NC_039582
A. yuanbaoshanensis	121,795	76,347	43,088	1,180	38.3	72	34	4	NC_050849

Large single-copy region; LSC, Small single-copy region; SSC, Inverted repeats; IR, Bolded species name indicate plastomes that were generated for this study.

Table S10. Summary of *Abies* mitochondrial genomes.

Taxon	Genome size (Mb)	Mitochondrial scaffolds	Scaffolds Len. (Mb)	GC content (%)	Protein genes	tRNA genes	rRNA genes	GenBank number or reference
A. koreana	1.17	1	-	45.9	41	9	3	ON897690
A. alba	1.43	11	1.43	45.98	41	9	3	ON378818-ON378828/ Kersten et al., 2022
A. firma	1.33	172	1.33	45.78	41			Kan et al, 2021
A. sibirica	1.49	237	1.49	45.7	41			Guo et al, 2020

Kan, S.-L., Shen, T.-T., Ran, J.-H. & Wang, X.-Q. Both Conifer II and Gnetales are characterized by a high frequency of ancient mitochondrial gene transfer to the nuclear genome. *BMC Biol.* **19**, 146, doi:10.1186/s12915-021-01096-z (2021).

Kersten, B. *et al.* The mitochondrial genome sequence of *Abies alba* Mill. reveals a high structural and combinatorial variation. *BMC Genomics* **23**, 776, doi:10.1186/s12864-022-08993-9 (2022).

Guo, W., Zhu, A., Fan, W., Adams, R. P. & Mower, J. P. Extensive Shifts from *Cis*- to *Trans*-splicing of Gymnosperm Mitochondrial Introns. *Mol. Biol. Evol.* **37**, 1615-1620, doi:10.1093/molbev/msaa029 (2020).
